# Ultra-large chemical libraries for the discovery of high-affinity peptide binders

**DOI:** 10.1038/s41467-020-16920-3

**Published:** 2020-06-23

**Authors:** Anthony J. Quartararo, Zachary P. Gates, Bente A. Somsen, Nina Hartrampf, Xiyun Ye, Arisa Shimada, Yasuhiro Kajihara, Christian Ottmann, Bradley L. Pentelute

**Affiliations:** 1 Department of Chemistry, Massachusetts Institute of Technology, Cambridge, MA 02139 USA; 2 Laboratory of Chemical Biology, Department of Biomedical Engineering and Institute for Complex Molecular Systems, Eindhoven University of Technology, PO Box 513, 5600 MB Eindhoven, Netherlands; 3 Department of Chemistry, Graduate School of Science, Osaka University, 1-1 Machikaneyama, Toyonaka, Osaka 560-0043 Japan; 4 The Koch Institute for Integrative Cancer Research, Massachusetts Institute of Technology, 500 Main Street, Cambridge, MA 02142 USA; 5 Center for Environmental Health Sciences, Massachusetts Institute of Technology, 77 Massachusetts Avenue, Cambridge, MA 02139 USA; 6 Broad Institute of MIT and Harvard, 415 Main Street, Cambridge, MA 02142 USA

**Keywords:** Mass spectrometry, Peptides, Combinatorial libraries

## Abstract

High-diversity genetically-encoded combinatorial libraries (10^8^−10^13^ members) are a rich source of peptide-based binding molecules, identified by affinity selection. Synthetic libraries can access broader chemical space, but typically examine only ~ 10^6^ compounds by screening. Here we show that in-solution affinity selection can be interfaced with nano-liquid chromatography-tandem mass spectrometry peptide sequencing to identify binders from fully randomized synthetic libraries of 10^8^ members—a 100-fold gain in diversity over standard practice. To validate this approach, we show that binders to a monoclonal antibody are identified in proportion to library diversity, as diversity is increased from 10^6^–10^8^. These results are then applied to the discovery of p53-like binders to MDM2, and to a family of 3–19 nM-affinity, α/β-peptide-based binders to 14-3-3. An X-ray structure of one of these binders in complex with 14-3-3σ is determined, illustrating the role of β-amino acids in facilitating a key binding contact.

## Abstract (web summary)

Synthetic peptide libraries can access broad chemical space, but generally examine only ~ 10^6^ compounds. Here, the authors show that in-solution affinity selection, interfaced with nLC-MS/MS sequencing, can identify binders from fully randomized synthetic libraries of 10^8^ members.

## Introduction

Drug discovery benefits from the ability to assay a large number of compounds for activity against a biomolecular target of interest. This target-based modality remains prominent in the pharmaceutical industry, and it is estimated that of all 113 first-in-class drugs approved by the FDA between 1999 and 2013, 71% were discovered using a target-based approach^1^. While a variety of techniques are used to this end, some of the most common strategies include high-throughput screening (HTS) and fragment-based drug discovery (FBDD)^2^ for identification of small molecule binders, and protein engineering strategies^3^ for discovery of novel biologics.

In the past decade, much research attention has been devoted to discovering and engineering peptide-based binders as an emerging therapeutic modality^4,5^. Given the unique niche of chemical space they occupy, possessing molecular weights in between those of small molecules (<500 Da) and biologics (up to ~150,000 Da), peptides offer a distinct profile of chemically attractive features. Peptides are synthetically accessible, amenable to chemical tailoring, and have the potential to bind the typically shallow surfaces seen in therapeutically relevant—and historically intractable—protein–protein interactions (PPIs)^6–8^. Importantly, chemical modifications, such as non-canonical amino acid incorporation, head-to-tail macrocyclization, and chemical stapling, can render peptides more proteolytically stable, more cell-penetrant, and even increase binding affinity relative to their natural, underivatized counterparts, which on their own tend to exhibit poor pharmacological properties^9–13^.

**Fig. 1:**
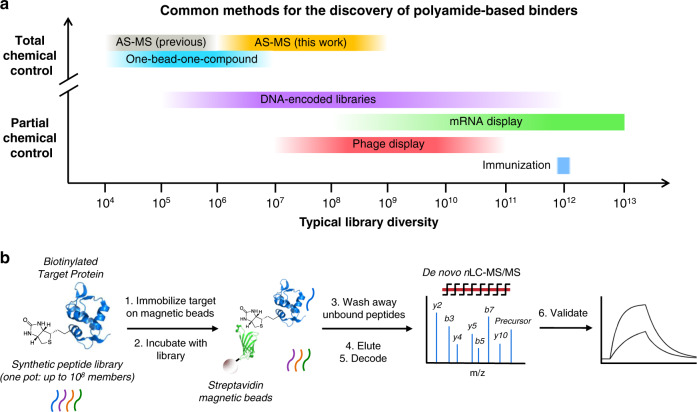
AS-MS enables discovery from randomized, high-diversity chemical libraries. **a** Among existing techniques for peptide binder discovery, increased chemical control over library synthesis and screening typically comes at the cost of limited library diversity. Affinity selection-mass spectrometry (AS-MS; this work), which relies on chemically accessed libraries and direct identification of active members, can investigate library diversities on the order of 10^8^–10^9^ while maintaining a high degree of chemical control over the synthesis and selection process. **b** A typical AS-MS workflow, which uses magnetic beads as a partitioning reagent to discriminate bound from unbound library members, and nano-liquid chromatography-tandem mass spectrometry to identify sequences of active peptides.

Molecular biology-based selection techniques, such as phage display^14,15^ and mRNA display^16^, are powerful tools for target-based discovery of novel peptide binders, thanks in part to their ability to examine enormous libraries (10^8^–10^13^ members)^17^. However, incorporation of a large number and variety of non-canonical amino acids by these methods remains challenging (Fig. 1a)^18,19^. Chemical combinatorial methods, such as DNA-encoded libraries (DELs)^20,21^ and one-bead-one-compound (OBOC) libraries^22,23^, can easily overcome this hurdle; however, each of these techniques faces its own limitations. DELs are typically limited to three or four varied positions, due to inefficiencies in the chemistry used for their assembly, and as such are more often categorized as libraries of small molecules. OBOC libraries can easily incorporate more varied positions, but OBOC screening is technically challenging and typically examines only ~10^6^ compounds^24^.

Affinity selection-mass spectrometry (AS-MS)^25–29^ represents an alternative strategy for target-based discovery of chemically accessed peptide binders. We recently leveraged LC-MS/MS for sequencing individual synthetic peptides present in complex mixtures^30^, and to increase the diversity of synthetic peptide libraries amenable to AS-MS^12^, from ~10 to ~10^6^. This advance was used to select improved variants of known binders from small focused libraries (10^3^ members)^12^. Discovery of binders from fully randomized libraries is a much greater challenge, which may require library diversities considerably higher than are typically examined by chemical screening. In principle, commercial mass spectrometers are sufficiently sensitive to detect and sequence peptides from mixtures as complex as ~10^9^ (Table 1)^31,32^. However, it is not obvious whether single-pass affinity selections from such libraries could provide sufficient enrichment (i.e., reduce the number of peptides sufficiently) to identify binders by LC-MS/MS-based sequencing, which is applicable to mixtures of up to several thousand peptides^30^.

**Table 1:** Mixtures of up to 10^9^ peptides contain sufficient material for MS-based sequencing.

Library size	Member concentration (pM)	Moles per member (fmol)
10^6^	1000	1000
10^7^	100	100
10^8^	10	10
10^9^	1	1

At a given scale (1 mL), maximum diversity is a tradeoff between the amount of individual peptide required for nLC-MS/MS sequencing, and the solubility limit of the total peptide mixture (1 mM). Even more peptides might be examined concurrently at a fixed total concentration by increasing the selection volume.

Here, we show that magnetic bead capture-based AS-MS^33,34^ is capable of identifying binders from libraries of up to ~10^8^ random synthetic peptides (Fig. 1b). Starting with an anti-hemagglutinin monoclonal antibody (anti-HA mAb) selection target, we demonstrate that high-affinity binders can be captured with near-quantitative recovery from relevant ligand concentrations. These results translate into a selection context, where high-affinity binders containing the HA epitope are identified in proportion to library diversity over the range of 10^6^–10^8^. Similarly conducted selections for MDM2 binding identify p53-like peptides for 10^8^-member libraries only, illustrating the utility of high-diversity synthetic libraries for identifying binders previously accessible only to molecular biology-based approaches. The power of the synthetic library approach for identifying binders not otherwise accessible is illustrated by the discovery of mixed α/β, phosphoserine-containing binders to the hub protein 14-3-3.

## Results

### AS-MS recovers high-affinity ligands from high dilution

Identification of binders from high-diversity libraries would necessitate their efficient recovery at high dilution. To assess the utility of magnetic bead-based affinity capture for this purpose, we investigated the recovery of a number of peptide binders with varying affinity for anti-HA mAb clone 12ca5 (12ca5), which recognizes the linear motif DXXDYA (Supplementary Figs. 1–6; Supplementary Table 1). In these experiments, streptavidin-coated magnetic beads (1 mg; 0.13 nmol IgG binding capacity) functionalized with biotinylated 12ca5 were incubated with mixtures of 12ca5-binding peptides (either 1 nM/peptide, 100 pM/peptide, or 10 pM/peptide; 1 mL scale). The magnetic beads were isolated, washed with buffer, and treated with chemical denaturant to elute bound peptides. A portion of this eluate (~2%) was subjected to nLC-MS analysis, and recovery was quantified using normalized MS response. To calculate recovery, a separate dose-response curve was generated for each peptide, since their MS responses varied by ~5-fold (Supplementary Fig. 7).

**Fig. 2:**
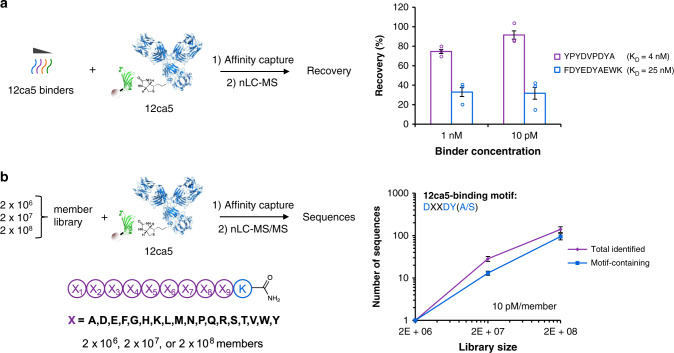
Magnetic bead capture recovers high-affinity binders from both simple mixtures and random libraries. **a** High-affinity 12ca5-binding peptides were recovered effectively from a concentration of 10 pM, which corresponds to the concentration of an individual library member in a 10^8^-member library at 1 mM total peptide. Recoveries were determined by MS detector response, normalizing to an internal standard. **b** The number of identified sequences bearing the 12ca5-binding motif—DXXDY(A/S)—increased in proportion to library diversity, from two million to two hundred million-member libraries. On average, motif-containing sequences represented 71% of all sequences identified. Error bars correspond to one standard deviation among three technical replicates.

Under the conditions examined, only the two highest affinity binders were significantly retained, with recoveries of 75% and 33% obtained for ~4 and ~25 nM-affinity binders, respectively (Fig. 2a; Supplementary Figs. 8 and 9). This result is consistent with the work of Sannino and coworkers, who observed low recoveries for micromolar affinity ligands in the context of DELs^35^. We tested the use of increased magnetic bead concentration to recover lower affinity ligands; however, this parameter had little effect on recovery, perhaps suggesting that these ligands are lost during the washing step (Supplementary Fig. 10). Consistent with this view, recovery of the 25 nM-affinity ligand (33%) is in quantitative agreement with that expected based on its measured dissociation rate, and the total wash time of ~6 min (34%, based on *k*_off_ = 3.0 × 10^−3^ s^−1^; Supplementary Fig. 6). Therefore, dissociation rate is an important factor in controlling the recovery of binders by AS-MS. For the retained binders, no loss in recovery was observed as their initial concentration was decreased from 1 nM to 10 pM, suggesting that selections from libraries as diverse as 10^8^ would be feasible (Supplementary Figs. 8 and 9; Table 1). Below 10 pM, low MS detector response precluded convenient quantitation.

We examined whether recovery of high-affinity binders could be maintained at concentrations below those conveniently quantified. Affinity captures were performed at 10 and 1 pM ligand concentration (10 or 1 fmol/peptide, respectively), and the eluates were concentrated by solid phase extraction such that the majority of the bound fraction could be analyzed (~80%). Although recoveries could be maintained down to 1 pM ligand, the resulting MS signals were observed near the intensity threshold that would be used for MS/MS precursor isolation during an actual selection, to facilitate collection of high quality spectra (5 × 10^4^ counts; Supplementary Fig. 11). The precursor selection threshold defines a limit of detection for sequenced peptides, which corresponds to ~50% or ~5% recovery of peptides initially present at 1 pM (1 fmol/mL) or 10 pM (10 fmol/mL), respectively. Therefore, 10^9^ is an upper bound on the library diversity amenable to this AS-MS approach at the examined scale (1 mL), to retain sufficient material for nLC-MS/MS sequencing (Table 1).

### AS-MS enriches 12ca5 binders from a 10^6^-member library

To assess whether magnetic bead-based pulldowns could recover binders from complex mixtures and reduce the mixture complexity sufficiently for nLC-MS/MS sequencing, we synthesized a library of design (X)_9_K, where X = all l-amino acids except for cysteine and isoleucine (theoretical diversity = 2 × 10^11^), by split-and-pool synthesis^22,36^ on 30 µm TentaGel resin (2.9 g; 2 × 10^8^ beads), for use in subsequent selections (Supplementary Fig. 12). Portions of this library comprising 2 × 10^7^ and 2 × 10^6^ members were taken prior to cleavage from resin to give three libraries of increasing size from the same batch of split-and-pool synthesis (Fig. 2b). Selections against 12ca5 (0.13 nmol) were then performed with the 2 × 10^6^-member library, at 10 pM/member concentration (1 mL scale; 10 fmol/peptide, 20 nmol total library), with the goal of identifying sequences similar to the HA epitope (selections were performed in triplicate; Supplementary Note 1). Selection eluates were concentrated by solid phase extraction, and analyzed by nLC-MS/MS (theoretical loading of a retained peptide: 8 fmol). From these selections, just one peptide that matched the library design was identified with a sequencing score (average local confidence (ALC) score^37^) ≥ 80 (MN**D**LV**DYA**DK; Supplementary Table 2). This sequence had five residues in common with the HA epitope, DXVDYA, including the hot spot residues Asp4, Asp7, Tyr8, and Ala9 required for high-affinity 12ca5-binding (Supplementary Figs. 13–21)^38^.

We endeavored to understand the enrichment achieved by the selection, where enrichment is defined as:

1 $$	\left( {{\mathrm{sequenced}}\;{\mathrm{binders/total}}\;{\mathrm{sequenced}}\;{\mathrm{peptides}}} \right)_{{\mathrm{isolated}}}{\mathrm{/}}\\ 	 \left( {{\mathrm{binders/total}}\;{\mathrm{peptides}}} \right)_{{\mathrm{assayed}}}$$

or equivalently

2 $$	\left( {{\mathrm{sequenced}}\;{\mathrm{binders}}} \right)_{{\mathrm{isolated}}}{\mathrm{/}}\left( {{\mathrm{binders}}} \right)_{{\mathrm{assayed}}}\, \ast\, \left( {{\mathrm{total}}\;{\mathrm{peptides}}} \right)_{{\mathrm{assayed}}}{\mathrm{/}}\\ 	\left( {{\mathrm{total}}\;{\mathrm{sequenced}}\;{\mathrm{peptides}}} \right)_{{\mathrm{isolated}}}$$

Achieving high enrichment in a single selection step would be essential for discovering rare binding molecules by AS-MS, since synthetic libraries cannot be propagated to facilitate sequential rounds of selection. Since only a single peptide was sequenced from the selection eluate, a maximum enrichment of 2 × 10^6^ was achieved. The actual enrichment may be significantly lower, if additional binders were present in the library, but not recovered and sequenced. For example, the expected number of DXXDY(A/S)-containing sequences in the 2 × 10^6^-member library is 152 (although not all of these are necessarily high-affinity binders), suggesting the actual enrichment could be as low as ~1.3 × 10^4^ (Supplementary Note 2).

To check whether additional peptides were recovered by the selection at lower abundance, we performed a selection at 200 pM/member, such that less-abundant peptides could cross the precursor selection threshold (Supplementary Fig. 22). Increasing amounts of the selection eluate were analyzed by nLC-MS/MS, with sample loadings varied from 8 fmol/member (selection threshold: ~5% recovery) to 75 fmol/member (selection threshold: ~0.5% recovery). At the lowest sample loading, just one peptide was identified (MN**D**LV**DYA**DK), identical to that observed in the original selection. As sample loading was increased to 75 fmol/member, two additional epitope-containing peptides were identified: P**D**VH**DY**TWGK and EN**D**WQ**DYS**HK. However, recovery of these sequences came at the cost of identifying additional non-motif-containing peptides: a total of 32 from 3 replicate selections. These results suggest that with respect to recovery of binders, little benefit is conferred by higher library concentration, and that 8 fmol/member sample loading avoids detection of peptides present in the selection eluate at lower abundance. Accounting for these additional sequences yields an enrichment of 1.1 × 10^3^, assuming 152 DXXDY(A/S)-containing peptides (Eq. (2)), which is on the order of enrichments reported for individual rounds of mRNA (~10^3^)^39^, phage (~10^3^)^14^, or cell surface display (~10^4^)^40,41^.

Taken together, these results suggest that magnetic bead affinity capture can facilitate selections from complex mixtures of synthetic peptides at a concentration of 10 pM/member. In conjunction with nLC-MS/MS peptide sequencing, very high enrichment for sequenced peptides is enabled by the use of a precursor selection threshold, to detect only those peptides that are significantly recovered by the selection (here, a DXXDY(A/S)-containing peptide—present in highest abundance). Peptides recovered in lower abundance (here, mostly non-motif-containing peptides) are present in the selection eluate, but not sequenced (Supplementary Table 4). As described below (‘Parallel selections distinguish non-specific binders’), the majority of these peptides are non-specific binders.

### Enrichment is maintained as diversity increases from 10^6^-10^8^

To investigate whether comparable selection performance could be achieved using higher-diversity libraries, we assayed 2 × 10^7^- and 2 × 10^8^-member libraries in selections for 12ca5 binding. In theory, these libraries should contain 10- and 100-fold more DXXDY(A/S) peptides compared to the 2 × 10^6^-member library. If selections from the higher-diversity libraries performed comparably, then they should recover all of these additional DXXDY(A/S)-containing peptides, with no increase in the proportion of non-binding peptides. For each library, selections were performed in triplicate, with library concentration maintained at 10 pM/member and using 0.13 nmol of selection target, as above.

From the 2 × 10^7^-member library, a representative selection identified 23 peptides that matched the library design (ALC ≥ 80), 14 of which (61%) contained DXXDYA or DXXDYS (Fig. 2b; Supplementary Table 2). An additional two sequences closely resembled 12ca5 binders—KVL**D**Y**DYA**WK and Y**D**DR**YAD**TFK—and may correspond to inaccurate sequence assignments. Selections from the 2 × 10^8^-member library identified 156 total peptides (ALC ≥ 80), 109 of which (70%) contained either DXXDYA or DXXDYS (Fig. 2b; Supplementary Tables 2-3). These results illustrate that selections identified binders from the higher-diversity libraries without loss in recovery, since the expected ~10 and ~100 DXXDY(A/S)-containing peptides were identified, and without loss in enrichment, since DXXDY(A/S)-containing peptides comprised the majority of selected peptides in each case (100%, 61%, and 70% for 2 × 10^6^, 2 × 10^7^, and 2 × 10^8^-member libraries, respectively). Therefore, we concluded that single-pass AS-MS could be applied to libraries of at least 10^8^ random peptides without loss in performance.

### Recovery of binders drops from libraries beyond 10^8^ members

We next set out to understand whether libraries of diversity beyond 10^8^ would also be amenable to AS-MS. To access 10^9^ random synthetic peptides on a convenient lab scale, we used 20 µm TentaGel resin beads (vs. 30 µm beads, above) to prepare a library of design (X)_9_K (X = all l-amino acids except for cysteine and isoleucine; 5.4 g; 1.3 × 10^9^ beads) (Supplementary Fig. 23). As above, a portion of the beads was set aside prior to cleavage from resin to give a 10^8^-member library for side-by-side comparison.

The 10^8^-member library performed comparably to the 10^8^-member library prepared on 30 µm TentaGel beads (above), yielding 257 total peptides (ALC ≥ 80), 183 of which (71%) contained either DXXDYA or DXXDYS. In contrast, selections from the 10^9^-member library identified only 34 peptides (ALC ≥ 80), 21 of which (62%) contained DXXDYA or DXXDYS (Supplementary Table 5). For the 10^9^-member library, selections were performed at 2 pM/member to maintain solubility; however, the lower sequence identification rate cannot be attributed to material losses alone, since selection from a 10^8^-member performed at 2 pM/member identified 131 DXXDY(A/S) sequences (vs. 183 from the selection at 10 pM/member; Supplementary Table 5). Analysis of pooled eluates from replicate selections from the 10^9^-member library yielded no additional sequences, providing further evidence that material losses were not responsible for the reduction in sequence identification rate (since similar populations of DXXDY(A/S)-containing sequences are recovered by replicate selections) (Supplementary Tables 3 and 6).

Since affinity selections involve a large number of potential binders competing for a limited number of binding sites, it is possible that many weaker binders present in the library reduce the individual recoveries of all ligands. Two experiments were performed to address whether competition was responsible for the lower recovery of DXXDY(A/S) peptides from the 10^9^-member library. First, we performed a selection using 10-fold higher stoichiometry of 12ca5 (1.3 nmol) relative to library (2 fmol/member). If competition were limiting recovery of binders, this experiment should have recovered additional DXXDY(A/S)-containing peptides. Instead, their recovery was abrogated entirely (Supplementary Table 7). Second, to determine the frequency of binders that would be required for competition to become significant, we studied the effect of exogenous competitors on the recovery of DXXDY(A/S) peptides from the 10^8^-member library. In all, 4 nM– or 3 µM–affinity competitors required concentrations of 100 nM or 100 µM, respectively, to attenuate the recovery of DXXDY(A/S) peptides (Supplementary Fig. 24; Supplementary Table 8**)**. These concentrations correspond to 5 × 10^4^ or 5 × 10^7^ peptides (present at 2 pM/peptide), suggesting that: (1) the expected ~10^3^ DXXDY(A/S) peptides in the 10^9^-member library would not compete for selection target; and (2) in order for µM–affinity binders to compete with DXXDY(A/S) peptides, they would need to comprise 5% of the 10^9^-member library.

The combined results are consistent with the interpretation that selections from the 10^9^-member library yield more peptides than are compatible with nLC-MS/MS sequencing. As the number of peptides in the selection eluate (the sample complexity) increases, the proportion of sequenced peptides decreases^30^. This drop in sequencing coverage was observed previously to be particularly significant beyond 10^3^ peptides^30^, which is consistent with the approximate number of total peptides (binders and non-binders) likely isolated from the 10^9^-member library (>3.5 × 10^3^, based on a maximum of 35 peptides isolated from the 10^6^-member library). Additionally, the competition experiments provide strong evidence that selections took place under conditions of equilibrium binding, since the relative strength of the two competitors was in proportion to their binding constants. Therefore, predictable competition effects should be expected when AS-MS is conducted in the presence of added competitor, or from focused libraries containing a higher proportion of binders.

### Parallel selections distinguish non-specific binders

**Fig. 3:**
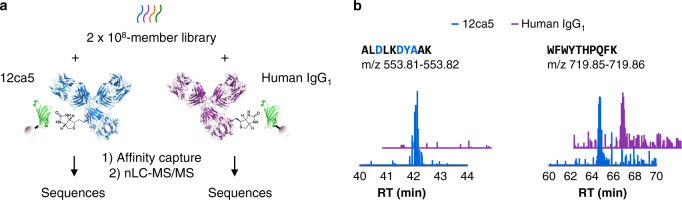
Parallel selections enable differentiation of specific and non-specific binders. **a** Using AS-MS, the same population of compounds can be used in selections against more than one target in parallel, enabling assessment of target-dependent enrichment of peptides. Here, selections using a 2 × 10^8^-member library were performed against 12ca5 and a polyclonal human IgG_1_ in parallel, followed by sequential nLC-MS/MS analysis. **b** Extracted ion chromatograms of a DXXDY(A/S)-containing peptide (left) and a non-motif-containing peptide (right) from a selection against 12ca5 (blue) and polyclonal human IgG_1_ (purple). The peptide containing the 12ca5-binding motif is selectively enriched towards 12ca5, while the non-motif-containing peptide is promiscuously pulled down in both conditions.

Selections from identical portions of synthetic peptide mixtures obtained by split-and-pool synthesis are readily conducted in parallel. For example, a 2 × 10^8^-member library synthesized on 30 μm TentaGel (~3.7 pmol/bead) provides sufficient material for ~370 selections performed at 10 fmol/member scale. We leveraged this capability to understand what proportion of non-HA epitope-containing peptides were common to an unrelated selection target, and therefore attributable to non-specific binding^42^. Side-by-side selections from a 2 × 10^8^-member library were conducted in triplicate against both 12ca5 (mouse IgG_2b_κ) and a polyclonal human IgG_1_, with the goal of quantifying the degree of overlap among selected peptides (Fig. 3a).

Three replicate 12ca5 selections yielded a total of 133 DXXDY(A/S)-containing peptides, all of which were specific for 12ca5, along with 65 non-motif containing peptides (Fig. 3b; Supplementary Table 9). Of the non-motif-containing peptides, 18 contained motifs that differed from the HA epitope by one position (for example, EXXDYA). Of the remaining 47 peptides, closer inspection revealed that 13 may contain mis-sequenced HA epitopes. For example, VFQDWEDFSK and YMDTVDFSEK contain the FS dipeptide fragment, which is isobaric to YA (Supplementary Fig. 25). A total of 34 peptides had no obvious sequence similarity to the DXXDY(A/S) motif. 17 of these were also sequenced from the IgG_1_ selections. Examination of the LC-MS data revealed that many or all of the remaining 17 peptides were also present in the IgG_1_ selection eluates, but not sequenced (9 of 9 selected peptides examined; Supplementary Fig. 26). Therefore, essentially all of the non-motif-containing peptides sequenced from 12ca5 selection can be attributed to non-specific binding. Presumably, many additional non-motif-containing peptides were recovered at lower abundance and not sequenced, as for the 10^6^-member library.

### High diversity enables discovery of MDM2-binding peptides

Having established magnetic bead-based AS-MS as a selection protocol applicable to high-diversity libraries of random synthetic peptides, we sought to benchmark its performance relative to affinity selection from genetically-encoded libraries. As a model target for this purpose, we selected MDM2, an oncogenic ubiquitin ligase that binds its substrate p53 through a FXXXWXXL motif. Phage display has identified a number of well-characterized, high-affinity MDM2 binders based on this motif^15,43–45^, and we sought to determine whether AS-MS could recapitulate these results by identifying similar sequences from synthetic libraries of comparable size and design.

**Fig. 4:**
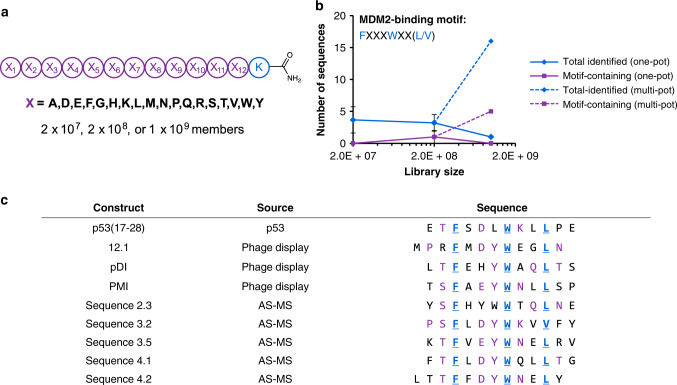
High-diversity libraries facilitate discovery of p53-like peptides. **a** Design of libraries used in selections against MDM2. Each library was derived from the same batch of split-and-pool SPPS. The readout for selections was the number of sequences bearing the MDM2-binding motif, FXXXWXX(L/V). **b** Motif-containing sequences could be identified by selection from individual 2 × 10^8^-member libraries (one-pot), but not from 2 × 10^7^-member or 1 × 10^9^-member libraries (obtained by pooling the five individual 2 × 10^8^-member libraries; one-pot). Selections from the five individual 2 × 10^8^-member libraries (multi-pot; 1 × 10^9^ members total) identified five motif-containing sequences, suggesting that library size was enabling for identification of p53-like peptides as long as the mixture was not overly complex. Error bars correspond to one standard deviation among three technical replicates. **c** Sequences of known MDM2-binding peptides, including the native MDM2-binding epitope of p53 (residues 17–28) and three peptides identified from phage display, aligned with sequences identified from **b**. Known hot spot residues (F, W, L/V) are indicated in blue; aligned hot spot positions are indicated in bold and underline; and additional conserved residues are indicated in purple.

To mimic previous phage display libraries, a library of design (X)_12_K, where X = all l-amino acids except cysteine and isoleucine (theoretical diversity = 1.2 × 10^15^), was synthesized on 20 µm TentaGel resin (4.2 g; 1.3 × 10^9^ beads). Prior to cleavage from resin, this library was split to yield five distinct 2 × 10^8^-member libraries, as well as a 2 × 10^7^-member library, to investigate the importance of library size in the context of selections for MDM2 binding (Fig. 4a; Supplementary Fig. 27). The N-terminal domain of MDM2 (residues 25–109) was accessed synthetically in biotinylated form, to enable its use as a selection target in conjunction with streptavidin-coated magnetic beads (Supplementary Fig. 28).

Selections from three of the five 2 × 10^8^-member libraries against (25–109)MDM2 (0.13 nmol) yielded sequences containing the FXXXWXX(L/V) motif characteristic of MDM2-binding (Fig. 4b). In total, 16 sequences from these selections were identified (ALC ≥ 80), five of which (31%) contained FXXXWXX(L/V) (Supplementary Table 10). An additional two sequences appeared to be MDM2-binding peptides, containing the minimal FXXXW motif, but were potentially mis-sequenced due to poor-quality fragmentation spectra (Supplementary Fig. 29). Selections from the 2 × 10^7^-member library did not yield motif-containing sequences, consistent with the frequency of binders identified from 2 × 10^8^-member libraries. Selections were also performed from a 10^9^-member library, obtained by pooling the individual 2 × 10^8^-member libraries. These selections failed to identify motif-containing sequences, consistent with the poor recovery of motif-containing sequences from 10^9^-member libraries in selections against 12ca5. In summary, library diversity correlates with number of binders identified, provided the diversity is within the technical limits of AS-MS for decoding single-pass selections.

A closer examination of the FXXXWXX(L/V)-containing sequences identified here alongside known MDM2-binding peptides revealed sequence similarity outside of the FWL triad (Fig. 4c). Specifically, a 6-residue motif was observed among the majority of sequences: (S/T)FX(D/E)YWXXL. Each of the conserved positions corresponds to a hot spot of binding energy, as determined by mutational analysis^46^. The ability to identify not only the FWL triad but also other significant determinants of binding affinity supports our interpretation that AS-MS is capable of matching the performance of phage display, in the context of selections against MDM2. While others have demonstrated the utility of synthetic libraries for identifying MDM2 binders^42,47^, our results illustrate their utility for mapping the determinants of a protein-protein interaction.

### AS-MS identifies non-canonical 14-3-3γ-binding peptides

To illustrate a key advantage of the synthetic library approach, we investigated whether AS-MS could achieve comparable selection performance from a library based on non-canonical amino acids. As a selection target, we chose the hub protein 14-3-3, which interacts with a range of disease-relevant proteins including p53, Raf kinases, and estrogen receptor α. Considerable effort has been devoted to developing modulators of these interactions^48^, which are generally mediated by phosphorylation^49^.

**Fig. 5:**
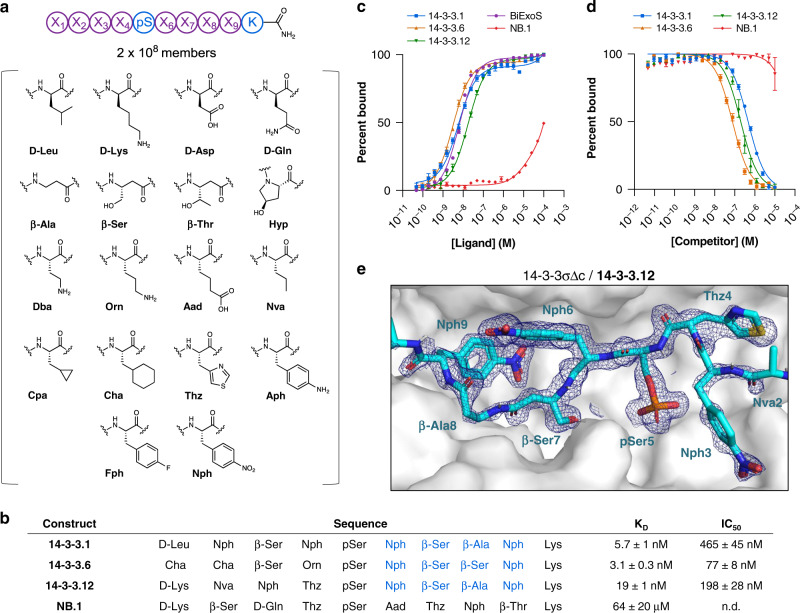
Synthetic libraries identify a 14-3-3γ-binding consensus based on β-amino acids. **a** Design of a non-canonical library used in selections against 14-3-3γ. A phosphoserine was fixed in the middle, flanked by four varied positions on either side. A suite of non-canonical amino acids was incorporated at each varied position. **b** Sequences of putative 14-3-3γ-binding peptides (**14-3-3.1**, **14-3-3.6**, and **14-3-3.12**) identified from affinity selections, as well as a negative control peptide (non-binder or **NB.1**, identified as an artifact in selections against 12ca5) chosen for resynthesis and binding validation studies. Residues comprising the conserved C-term motif among 14-3-3γ binders—Nph-β-Ser-(β-Ala/β-Ser)-Nph—are indicated in blue. **c** Identified binders exhibited nanomolar affinities for 14-3-3γ, as determined by fluorescence anisotropy of FITC-labeled 14-3-3γ-binding peptides, NB.1, and a known 14-3-3γ-binding peptide (BiExoS; positive control). These affinities were approximately 10,000-fold higher relative to the negative control. K_D_ values are given in **b**. Uncertainties correspond to 95% confidence intervals derived from nonlinear regression. **d** Identified 14-3-3γ-binders could compete off bound BiExoS in a fluorescence anisotropy competition assay, suggesting they bind 14-3-3γ in the canonical, amphipathic binding groove. IC_50_ values are given in **b**. **e** Molecular structure of 14-3-3σΔc (white surface) in complex with **14-3-3.12** (cyan sticks), based on a 1.80 Å crystal structure. The 2Fo-Fc electron density map corresponding to **14-3-3.12** is shown (blue mesh), contoured at 1σ. Abbreviations: β-Ala β-alanine; β-Ser β-homoserine; β-Thr β-homothreonine; Aad aminoadipic acid; Aph 4-aminophenylalanine; Cha cyclohexylalanine; Cpa cyclopropylalanine; Dba diaminobutyric acid; Fph 4-fluorophenylalanine; Hyp hydroxyproline; Nph 4-nitrophenylalanine; Nva norvaline; Orn ornithine; pSer phosphoserine; Thz thiazolylalanine. Error bars correspond to standard error among three technical replicates.

For use in selections against 14-3-3, we designed a library based on a fixed phosphoserine, flanked by eight varied positions (Fig. 5a). At each varied position, we incorporated one of 18 non-canonical amino acids—including β- and D-amino acids—encompassing a variety of polar, non-polar, charged, and aromatic side-chain functionalities (Fig. 5a) (theoretical diversity = 1.1 × 10^10^). This library was synthesized on 30 µm TentaGel resin (2.9 g; 2 × 10^8^ beads), yielding 2 × 10^8^ members (Supplementary Fig. 30).

Side-by-side selections were performed against the 14-3-3γ isoform, and 12ca5 (negative control, to identify non-specific binders; 0.13 nmol each). These selections yielded a total of 19 sequences that matched the library design (ALC ≥ 80), 17 of which were unique to 14-3-3γ, and 2 of which were unique to 12ca5 (Supplementary Table 11; Supplementary Fig. 31). Extracted ion chromatograms were used to verify the absence of 14-3-3γ-unique sequences from the 12ca5 selections (Supplementary Figs. 32 and 33). Among the 14-3-3γ-unique sequences, seven contained a C-terminal motif: (β-homoserine)-(β-alanine/β-homoserine)-(4-nitrophenylalanine). In general, d-amino acids were not present within the sequences identified. β-amino acids were enriched at positions 3, 7, and 8, but were otherwise largely absent. A positional frequency analysis revealed additional preferences for cyclohexylalanine at the N-terminus, and a β-homoserine at position three (Supplementary Fig. 31).

### Discovered peptides bind 14-3-3γ with low nanomolar affinity

To test the binding affinity of putative 14-3-3γ-binding peptides that contained the C-terminal (β-homoserine)-(β-alanine/β-homoserine)-(4-nitrophenylalanine) motif, we synthesized fluorophore-labeled forms of three selected peptides, for use in a fluorescence anisotropy binding assay (Fig. 5b; Supplementary Figs. 34–37). As a negative control, we employed a sequence identified from a 12ca5 selection. Each of the putative 14-3-3γ-binding peptides examined exhibited low nanomolar affinity for 14-3-3γ, with K_D_ values ranging from 3 to 19 nM (Fig. 5c). By contrast, the negative control peptide exhibited ~10,000-fold weaker binding, suggesting that the specific amino acid sequences of the 14-3-3γ-binding peptides—not the phosphoserine alone—were required for their identification.

Unlabeled forms of the non-canonical 14-3-3γ binders were assayed for their ability to compete with BiExoS, a peptide ligand derived from the *Pseudomonas aeruginosa* cytotoxin Exoenzyme S^50^, in a fluorescence anisotropy competition assay (Supplementary Figs. 38–41). This experiment would test whether the peptides bind to the amphipathic 14-3-3γ binding groove, or elsewhere on the protein. The non-canonical 14-3-3γ binders were found to compete off BiExoS with IC_50_ values ranging from 78 to 530 nM, suggesting that they indeed bind in the canonical, phosphopeptide-accepting binding channel on 14-3-3γ (Fig. 5d)^48^. By contrast, the negative control peptide showed no inhibitory activity.

### β-amino acids facilitate a key binding contact with 14-3-3

As an additional means of characterizing the binding interaction of non-canonical peptides with 14-3-3, we crystallized **14-3-3.12** in complex with 14-3-3σ. 14-3-3σ was used in place of 14-3-3γ to facilitate crystallization, and retained most of the binding activity for **14-3-3.12** (Supplementary Fig. 42). Diffraction data were collected to a resolution of 1.8 Å, and the structure was solved by molecular replacement (Supplementary Table 12).

The **14-3-3.12** backbone adopts an extended conformation in the 14-3-3γ binding groove, flanked by two half-turns^51^ defined by thiazolylalanine4 and β-alanine8 (Fig. 5e). 4-Nitrophenylalanine6, which was selected along with thiazolylalanine and 4-fluorophenylalanine at this position, makes hydrophobic contacts with Leu218, Ile219, and Leu222 of 14-3-3σ (Supplementary Fig. 43). 4-Nitrophenylalanine9—the residue most conserved by the selection—participates in an electrostatic interaction and/or H-bond with the ^ε^NH_3_ group of Lys122 (N–O distance=3.2 Å), and makes a hydrophobic contact with Ile168 (Supplementary Fig. 43). We speculate that the β-residues conserved at positions 7 and 8 of **14-3-3.12** provide the backbone flexibility necessary to accommodate these energetically-important interactions, which were not identified by selection from peptide libraries based on canonical amino acids^49^.

## Discussion

In this work, we demonstrate that affinity selection-mass spectrometry, using magnetic bead reagents, provides sufficient enrichment to identify high-affinity binders from randomized libraries of 10^8^ synthetic peptides. With respect to accessible diversity, this advance brings synthetic libraries up to the level of molecular biology-based combinatorial libraries. Diversity is a key determinant of selection outcome, as illustrated here for the discovery of p53-like binders to MDM2, and in the field of antibody engineering^41,52^. Therefore, the results described here can be expected to considerably extend the utility of synthetic libraries for discovering novel binding molecules.

The practical limit to library diversity amenable to single-pass AS-MS is 10^8^, beyond which the number of binders identified decreases. Our combined results are consistent with non-specific binding as the origin of this limit, which results in the recovery of more peptides from >10^8^-member libraries than can be sequenced by nLC-MS/MS with high coverage. Since diversity is limited by selection performance and nLC-MS/MS sequencing coverage, rather than peptide solubility, future work should focus on these areas. For example, multi-stage selections might be employed to improve enrichment further, and to reduce the number of peptides in a >10^8^-member library sufficiently for nLC-MS/MS sequencing. Sequencing coverage might also be improved by the use of specialized nLC columns and extended analysis times^53^.

The primary benefit of synthetic peptide libraries is the chemical control gained over the library design. Here, the combination of large library diversity and non-canonical amino acids led to the discovery of high-affinity 14-3-3γ-binding peptides that utilize β-amino acids to facilitate a binding contact. Given the comparatively low diversities examined by AS-MS relative to the upper bounds of genetically-encoded techniques (10^8^ vs. 10^13^), we anticipate that taking advantage of the chemical capabilities AS-MS affords—such as straight-forward non-canonical amino acid incorporation—may prove critical for more intractable targets. For example, AS-MS may be particularly suited to engineering peptide and peptoid foldamers^54,55^. Interfacing non-canonical amino acid incorporation with the macrocyclic architectures that have been rendered accessible to phage^56^ and mRNA display^57^ would further expand the breadth of chemical space amenable to exploration by AS-MS. In our case, performing selections on libraries of macrocycles would require an additional, post-enrichment linearization step for reliable MS/MS-based sequencing^58,59^. We envision that progress in these areas, along with improved mass spectral methods to enable investigation of libraries of even greater diversities, may ultimately facilitate discovery of fully non-natural peptide binders to historically undruggable targets.

## Methods

### Materials

H-Rink Amide-ChemMatrix resin was purchased from PCAS BioMatrix Inc. (St-Jean-sur-Richelieu, Quebec, Canada). In all, 30 μm TentaGel M NH2 microspheres (M30352; 0.20–0.25 mmol/g amine loading) were purchased from Rapp Polymere (Tübingen, Germany). In all, 20 μm TentaGel S NH2 microspheres (TMN-9909-PI; 0.2–0.3 mmol/g amine loading) was purchased from Peptides International (Louisville, KY). Fmoc-Ala-OH, Fmoc-Arg(Pbf)-OH, Fmoc-Asn(Trt)-OH, Fmoc-Asp(tBu)-OH, Fmoc-Gln(Trt)-OH, Fmoc-Glu(tBu)-OH, Fmoc-Gly-OH, Fmoc-His(Trt)-OH, Fmoc-Leu-OH, Fmoc-Lys(Boc)-OH, Fmoc-Met-OH, Fmoc-Phe-OH, Fmoc-Pro-OH, Fmoc-Ser(tBu)-OH, Fmoc-Thr(tBu)-OH, Fmoc-Trp(Boc)-OH, Fmoc-Tyr(tBu)-OH, and Fmoc-Val-OH were purchased from Advanced ChemTech (Louisville, KY). Fmoc-D-Asp(tBu)-OH, Fmoc-D-Gln(Trt)-OH, Fmoc-D-Leu-OH, and Fmoc-D-Lys(Boc)-OH were also purchased from Advanced ChemTech (Louisville, KY). 1-[Bis(dimethylamino)methylene]-1H-1,2,3-triazolo[4,5-b]pyridinium-3-oxid-hexafluorophosphate (HATU) was purchased from P3 BioSystems (Louisville, KY). 4-[(*R,S*)-α-[1-(9*H*-Fluoren-9-yl)-methoxyformamido]-2,4-dimethoxybenzyl]-phenoxyacetic acid (Fmoc-Rink amide linker), Fmoc-L-His(Boc)-OH, Fmoc-β-Ala-OH, and Fmoc-l-Lys(Alloc)-OH, and di-tert-butyl dicarbonate were purchased from Chem-Impex International (Wood Dale, IL). Fmoc-ß-cyclopropyl-l-alanine, Fmoc-ß-cyclohexyl-l-alanine, Fmoc-l-norvaline, Fmoc-*O*-tert-butyl-l-β-homoserine, Fmoc-*O*-tert-butyl-l-β-homothreonine, Fmoc-L-α-aminoadipic acid δ-tert-butyl ester, Nα-Fmoc-Nγ-Boc-l-2,4-diaminobutyric acid, Nα-Fmoc-Nδ-Boc-l-ornithine, Fmoc-3-(4-thiazolyl)-l-alanine, Fmoc-O-tert-butyl-l-trans-4-hydroxyproline, Fmoc-4-(Boc-amino)-L-phenylalanine, Fmoc-4-fluoro-l-phenylalanine, Fmoc-4-nitro-l-phenylalanine, and fluorescein isothiocyanate isomer I were also purchased from Chem-Impex International (Wood Dale, IL). Biotin-(PEG)4-NHS ester and biotin-(PEG)4-propionic acid were purchased from ChemPep Inc. (Wellington, FL). Peptide synthesis-grade *N*,*N*-dimethylformamide (DMF), dichloromethane (DCM), diethyl ether, HPLC-grade acetonitrile (MeCN), and HPLC-grade methanol (MeOH) were purchased from VWR International (Philadelphia, PA). Trifluoroacetic acid (TFA; for HPLC, ≥99%), piperidine (ReagentPlus; 99%), triisopropylsilane (98%), 1,2-ethanedithiol (≥98%), phenylsilane (97%), tetrakis(triphenylphosphine)palladium(0) (99%), and N-α-Fmoc-O-benzyl-L-phosphoserine were purchased from MilliporeSigma (St. Louis, MO). Diisopropylethylamine (99.5%; biotech. grade; DIEA) was also purchased from MilliporeSigma, and purified by passage through an activated alumina column (Pure Process Technology solvent purification system; Nashua, NH). Water was deionized using a Milli-Q Reference water purification system (Millipore).

Peptone from casein, granulated yeast extract, glycerol, and imidazole were purchased from Merck. LB Broth (Miller) powder, ampicillin sodium salt, potassium phosphate dibasic (≥99.0%), potassium phosphate monobasic, magnesium chloride hexahydrate (≥99.0%), β-mercaptoethanol (BME; ≥99.0%), and Trizma base (≥99.0%) were purchased from MilliporeSigma. Isopropyl β-D-Thiogalactopyranoside (IPTG) was purchased from PanReac AppliChem.

Mouse anti-hemagglutinin (HA) monoclonal antibody clone 12ca5 (anti-HA mAb 12ca5) and polyclonal human IgG_1_ were purchased from Columbia Biosciences (Frederick, MD). HyClone™ Fetal Bovine Serum (SH30071.03HI, heat inactivated) was purchased from GE Healthcare Life Sciences (Logan, UT). Bovine serum albumin (BSA; RIA grade) and Tween 20 (reagent grade) were purchased from Amresco (Solon, OH). Dynabeads MyOne Streptavidin T1 magnetic microparticles were purchased from Invitrogen (Carlsbad, CA).

Phosphate buffered saline (10x, Molecular biology grade) was purchased from Corning. Tris(hydroxymethyl)aminomethane (Tris) was purchased from J.T. Baker. 4-(2-hydroxyethyl)-1-piperazineethanesulfonic acid (HEPES; ≥99.5%), sodium bicarbonate (ACS grade, ≥99.7%), and magnesium chloride (≥98%) were purchased from MilliporeSigma. Tris(2-carboxyethyl)phosphine hydrochloride (TCEP) was purchased from Hampton Research (Aliso Viejo, CA). Sodium chloride (ACS grade) was purchased from Avantor. Guanidine hydrochloride (Technical grade) and sodium phosphate monobasic monohydrate (ACS grade) were purchased from Amresco.

### Manual fast-flow peptide synthesis

Manual fast-flow synthesis^60^ of peptide-^α^carboxamides was carried out on a 0.1 mmol scale, using H-Rink amide-ChemMatrix resin (0.45 mmol/g). Reagents and solvents were delivered to a stainless steel reactor containing the resin by either an HPLC pump (DMF or 20% (v/v) piperidine in DMF) or a syringe pump (active esters of Fmoc-amino acids). The reactor was submerged in a water bath for the duration of the synthesis and the temperature was maintained at 70 °C. The procedure for each coupling cycle included: a 30 s coupling with a mixture of Fmoc-protected amino acid (1 mmol), HBTU (0.95 mmol), and diisopropylethyl amine (DIEA; 2.9 mmol, 500 μL) in 2.5 mL of DMF, at a flow rate of 6 mL/min (for the coupling of tryptophan and histidine, 190 μL of DIEA was used to minimize racemization); 1 min DMF wash, at a flow rate of 20 mL/min; 20 sec deprotection with 20% (v/v) piperidine in DMF, at a flow rate of 20 mL/min; and 1 min DMF wash, at a flow rate of 20 mL/min. After each synthesis was complete, resins were washed with DCM (5x) and dried under reduced pressure.

### Automated fast-flow peptide synthesis

Automated fast-flow synthesis^61^ of peptide-^α^carboxamides was carried out using H-Rink amide-ChemMatrix resin (0.18 mmol/g, 0.03 mmol scale for N-terminal domain of MDM2; 0.45 mmol/g, 0.1 mmol scale for all other peptides). Syntheses were carried out at 90 °C; amide bond formation was effected in 8 s; and Fmoc removal was carried out in 8 s with 20% (v/v) piperidine in DMF. Individual cycle times were each ~40 s. After each synthesis was complete, resins were washed with DCM (5x) and dried under reduced pressure.

### Manual solid-phase synthesis of non-canonical peptides

Manual, batch synthesis of peptide-^α^carboxamides was carried out on a 0.05 mmol scale, using H-Rink amide-ChemMatrix resin (0.45 mmol/g). Resin was weighed into disposable fritted plastic syringes (Torviq), washed 3x with DMF, then swollen in DMF for ~1 h. Fmoc-protected amino acids (5 eq) in HATU solution (0.38M, 4.5 eq) were activated with DIEA (15 eq) and added to the resin bed. Reaction scales were variable and are described in the Supplementary Information. Couplings were allowed to proceed for 20 min At this time, reaction mixtures were drained and resins were washed 3x with DMF. Fmoc removal was carried out by treatment of the resin with 20% piperidine in DMF (1x flow wash; 2 × 5 min batch treatments). Following removal of N-terminal Fmoc group, resins were washed 3 × 5 mL with DMF, then 3 × 5 mL with DCM.

### Split-and-pool synthesis of peptide libraries

Split-and-pool synthesis was carried out on 30 μm TentaGel resin (0.26 mmol/g) for 10^8^-member libraries, and 20 μm TentaGel resin (0.26 mmol/g) for 10^9^-member libraries. Splits were performed by suspending the resin in DMF and dividing it evenly (via pipet) among 18 plastic fritted syringes on a manifold. Couplings were carried out as follows: solutions of Fmoc-protected amino acids, HATU (0.38M in DMF; 0.9 eq relative to amino acid), and DIEA (1.1 eq for histidine; 3 eq for all other amino acids) were each added to individual portions of resin. Couplings were allowed to proceed for 20 min Resin portions were recombined, and washed with DMF. Fmoc removal was carried out by treatment of the resin with 20% piperidine in DMF (1x flow wash; 2x 5 min batch treatments). Resin was washed again with DMF.

### Peptide cleavage and global deprotection

Global side chain deprotection and cleavage from solid support were carried out by treatment of dry resin with a solution of 94% (v/v) TFA, 2.5% (v/v) ethanedithiol, 2.5% (v/v) water, and 1.0% (v/v) triisopropylsilane, for 2 h at ambient temperature (~1.5 mL of deprotection solution/50 mg of resin). TFA was then evaporated under a stream of nitrogen, and crude peptide was precipitated by addition of cold diethyl ether. Precipitated peptide was triturated (3x) with cold diethyl ether, dissolved in 50/50 water/acetonitrile (0.1% TFA), passed through a 0.2 μm PTFE syringe filter, and lyophilized.

### Magnetic bead preparation

In all, 100 μL portions of MyOne Streptavidin T1 Dynabeads (10 mg/mL; 1 mg; 0.13 nmol IgG binding capacity) were transferred to 1.7 mL plastic centrifuge tubes, and placed in a magnetic separation rack (New England Biolabs, cat# S1506S). The beads were washed three times with blocking buffer (1 mg/mL BSA or 10% FBS, 0.02% Tween 20, 1x PBS), and then treated with portions of biotinylated target protein (~ 1.2 eq). The resulting suspensions were transferred to a rotating vertical mixer, and kept for 15 min at ambient temperature or 1 h at 4 °C. After this time, the beads were returned to the separating rack, the supernatant was removed, and the beads were washed 3x with blocking buffer.

### Magnetic bead capture of control binders

In all, 1 mL solutions containing blocking buffer (1 mg/mL BSA, 0.02% Tween 20, 1x PBS), and varying concentrations and amounts of 12ca5 control binders were prepared in 1.7 mL plastic centrifuge tubes, and chilled on ice for 10 min (the 12ca5 binders were added from mixtures containing 1 µM/peptide or 10 nM/peptide in 6 M guanidine hydrochloride, 200 mM phosphate, pH 7 buffer). The resulting chilled solutions were then added to 1 mg portions of 12ca5-functionalized magnetic beads, and the resulting suspensions were kept on a rotating vertical mixer (1 h, in 4 °C cold room). The centrifuge tubes containing the bead suspensions were then transferred to the magnetic separation rack. The beads were isolated, and washed three times with 1 mL each of chilled 1x PBS (beads were exposed to buffer for a total of ~6 min). Then, each drained bead aliquot was treated with 2 × 150 µL of elution buffer (6 M guanidine hydrochloride, 200 mM phosphate, pH 7.0 buffer containing 1 fmol/µL Peptide Retention Time Calibration Standard (PRTC; Pierce, cat# 88320, for use as an internal reference in MS-based quantitation)). For some conditions featuring lower binder concentration, eluates were concentrated via C18 ZipTip® pipette tips prior to nLC-MS analysis.

### Affinity selection from random libraries

Library (typically 10 fmol/member) was incubated with 100 μL (1 mg) portions of protein-immobilized magnetic beads in the presence of 10% FBS, 1x PBS (final volume: 1 mL) on a rotating mixer for 1 h at 4 °C. Typical final conditions: 1 mg/mL magnetic beads, 10 pM/member library. The centrifuge tubes containing the bead suspensions were transferred to the magnetic separation rack. The beads were washed 3 × 1 mL w/ 1x PBS. Bound peptides were eluted with 2 × 100 μL 6M guanidine hydrochloride, 200 mM phosphate, pH 6.8. Eluates were concentrated via C18 ZipTip® pipette tips and lyophilized. Powders were typically resuspended in 6 μL water (0.1% formic acid), and 5 μL were submitted for nLC-MS/MS analysis.

### nLC-MS/MS analysis

Analysis was performed on an EASY-nLC 1200 (Thermo Fisher Scientific) nano-liquid chromatography handling system connected to an Orbitrap Fusion Lumos Tribrid Mass Spectrometer (Thermo Fisher Scientific). Samples were run on a PepMap RSLC C18 column (2 μm particle size, 15 cm × 50 μm ID; Thermo Fisher Scientific, P/N ES801). A nanoViper Trap Column (C18, 3 μm particle size, 100 Å pore size, 20 mm × 75 μm ID; Thermo Fisher Scientific, P/N 164946) was used for desalting. The standard nano-LC method was run at 40 °C and a flow rate of 300 nL/min with the following gradient: 1% solvent B in solvent A ramping linearly to 61% B in A over 40 or 60 min, where solvent A = water (0.1% FA), and solvent B = 80% acetonitrile, 20% water (0.1% FA). Positive ion spray voltage was set to 2200 V. Orbitrap detection was used for primary MS, with the following parameters: resolution = 120,000; quadrupole isolation; scan range = 200–1400 m/z; RF lens = 30%; AGC target = 1 × 10^6^; maximum injection time = 100 ms; 1 microsan. Acquisition of secondary MS spectra was done in a data-dependent manner: dynamic exclusion was employed such that a precursor was excluded for 30 s if it was detected four or more times within 30 s (mass tolerance: 10.00 ppm); monoisotopic precursor selection used to select for peptides; intensity threshold was set to 5 × 10^4^; charge states 2–10 were selected; and precursor selection range was set to 200–1400 m/z. The top 15 most intense precursors that met the preceding criteria were subjected to subsequent fragmentation. Three fragmentation modes—collision-induced dissociation (CID), higher-energy collisional dissociation (HCD), and electron-transfer/higher-energy collisional dissociation (EThcD)—were used for acquisition of secondary MS spectra. Only precursors with charge states 3 and above were subjected to all three fragmentation modes; precursors with charge states of 2 were subjected to CID and HCD only. For all three modes, detection was performed in the Orbitrap (resolution = 30,000; quadrupole isolation; isolation window = 1.3 m/z; AGC target = 2 × 10^4^; maximum injection time = 100 ms; 1 microscan). For CID, a collision energy of 30% was used. For HCD, a collision energy of 25% was used. For EthcD, a supplemental activation collision energy of 25% was used.

### Direct fluorescence anisotropy

All direct fluorescence anisotropy affinity measurements were conducted in FA buffer (10 mM HEPES pH 7.4, 150 mM NaCl, 0.1% (v/v) Tween 20, 0.1% (w/v) BSA). A 1:1 dilution series of 14-3-3γ (starting at 100 μM) was performed in polystyrene non-binding low-volume Corning Black Round Bottom 384-well plates (Corning 4514) containing a fixed concentration FITC-labeled peptide (10 nM or 50 nM). Measurements were performed at ambient temperature using a Tecan Infinite F500 plate reader with the following parameters: *λ*_ex_: 485 (20) nm; *λ*_em_: 535 (25) nm; mirror: Dichroic 510; flashes: 20; integration time: 50 μs; settle time; 0 μs; gain: manual 90; Z-position: calculated from well. The G-factor was set at 35 mP based on wells containing only the FITC-labeled peptide.

### Fluorescence anisotropy competition

All fluorescence anisotropy competition experiments were conducted in FA buffer (10 mM HEPES pH 7.4, 150 mM NaCl, 0.1% (v/v) Tween-20, 0.1% (w/v) BSA). A 1:1 dilution series of unlabeled peptides (starting at 10 μM) were performed in wells containing a fixed concentration FITC-labeled biExoS (10 nM) and 14-3-3γ (20 nM). Measurements were performed at ambient temperature using a Tecan Infinite F500 plate reader with the following parameters: *λ*_ex_ = 485 (20) nm; *λ*_em_ = 535 (25) nm, mirror: Dichroic = 510, flashes: 20; integration time: 50 μs; settle time: 0 μs; gain: manual 90; and Z-position: calculated from well. The G-factor was set at 35 mP based on wells containing only the FITC-labeled peptide.

### X-ray structure determination

Unlabeled **14-3-3.12** was soaked into preformed crystals of 14-3-3σΔc, which grew in 25% PEG400, 5% Glycerol, 0.2 M CaCl2, 0.1 M HEPES pH 7.5 plus 2 mM BME within two weeks. The soaked crystal was fished after 15 days of incubation and flash-frozen in liquid nitrogen. Diffraction data was collected at 100K on an in-house Rigaku Micromax-003 (Rigaku Europe, Kemsing Sevenoaks, UK) sealed tube X-ray source and Dectris Pilatus 200K detector (DECTRIS Ltd Baden-Daettwil, Switzerland). Integration, scaling and merging of data was done using DIALS (CCP4i2) after which molecular replacement is done with MOLREP (CCP4i2) using PDB 4JC3 as search model. A three-dimensional structure of **14-3-3.12** was generated using eLBOW (Phenix) after which it was built within this structure based on visual inspection of Fo-Fc and 2FoFc electron density maps in Coot. Several rounds of model building and refinement (based on isotropic b-factors and standard set of stereo-chemical restraints: covalent bonds, angles, dihedrals, planarities, chiralities, non-bonded) were performed using Coot and Phenix.refine. See Supplementary Table 12 for data collection and refinement statistics. Structural coordinates have been deposited in the PDB (6TCH).

## Supplementary information

Supplementary Information

## Data Availability

The datasets generated during and/or analyzed during the current study are available from the corresponding author on reasonable request. The source data underlying Figs. 2b, 3b, and 4b, and Supplementary Figs. 12, 22–24, 27, and 30 are provided as Source data file. Source data are provided with this paper.

## Source data

Source data
