# Supplementary material for: Ultra-large chemical libraries for the discovery of high-affinity peptide binders
Source: Nat Commun. 2020 Jun 23;11:3183. doi: 10.1038/s41467-020-16920-3 (PMC7311396; doi:10.1038/s41467-020-16920-3)
Supplement: Supplementary file 1 — Supplementary Information [file 41467_2020_16920_MOESM1_ESM.pdf]

Ultra-large chemical libraries for the discovery of high-affinity peptide binders

Quartararo et al.

Supplementary Information

Supplementary Notes

Supplementary Note 1: Theoretical diversity vs. sampling diversity

Given the large theoretical diversity of this library relative to the diversity sampled (limited by the number of beads used in split-and-pool synthesis), it is unlikely the full HA epitope would be rediscovered. There is a 0.001% chance of finding a given 9-mer sequence in a library comprising 2×10^6 random 9-mers, with 18 possible monomers at each position (theoretical diversity = 2×10^{11}).

Supplementary Note 2: Probability of identifying a DXXDY(A/S)-containing peptide

The probability of any given sequence exhibiting the DXXDY(A/S) motif in a particular frame is equal to $(1/18)^3 \times (2/18)$, or 1.9×10^{-5}. With 9 varied positions, this motif can exist in four distinct frames; therefore the probability of finding this motif in any frame is equal to 7.6×10^{-5}. In a library of 2×10^6 members, there should then exist approximately 152 sequences containing DXXDY(A/S).

Supplementary Methods

Solid phase peptide synthesis (SPPS) of anti-hemagglutinin (HA) epitope and analogues:

Peptide-$^{\alpha}$carboxamides were synthesized on a 0.1 mmol scale, using H-Rink amide-ChemMatrix resin (0.45 mmol/g), using either fully automated[1] or manual[2] "fast flow" Fmoc-SPPS.

For automated syntheses: syntheses were carried out at 90 °C. Amide bond formation was effected in 8 s, and Fmoc removal was carried out in 8 s with 20% (v/v) piperidine in DMF. Individual cycle times were each about 40 s.

For manual flow-based syntheses: reagents and solvents were delivered to a stainless steel reactor, which contained the resin, by either an HPLC pump (DMF or 20% (v/v) piperidine in DMF) or a syringe pump (active esters of Fmoc-amino acids). The reactor was submerged in a water bath for the duration of the synthesis and the temperature was maintained at 70 °C. The procedure for each coupling cycle included: a 30 second coupling with a mixture of Fmoc-protected amino acid (1 mmol), HBTU (0.95 mmol), and diisopropylethyl amine (DIEA; 2.9 mmol, 500 μL) in 2.5 mL of DMF, at a flow rate of 6 mL/min (for the coupling of tryptophan and histidine, 190 μL of DIEA was used to minimize racemization); 1 min DMF wash, at a flow rate of 20 mL/min; 20 second deprotection with 20% (v/v) piperidine in DMF, at a flow rate of 20 mL/min; and 1 minute DMF wash, at a flow rate of 20 mL/min.

After each synthesis was complete, resins were washed with DCM (5x) and dried under reduced pressure.

Global side chain deprotection and cleavage from solid support were carried out by treatment of dry resin with a solution of 94% (v/v) TFA, 2.5% (v/v) ethanedithiol, 2.5% (v/v) water, and 1.0% (v/v) triisopropylsilane, for 2 h at ambient temperature (~1.5 mL of deprotection solution/50 mg of resin). TFA was then evaporated under a stream of nitrogen, and crude peptide was precipitated by addition of cold diethyl ether. Precipitated peptide was triturated (3x) with cold diethyl ether, dissolved in 50/50 water/acetonitrile (0.1% TFA), passed through a 0.2 μm PTFE syringe filter, and lyophilized.

Crude peptides were purified by semipreparative reverse phase HPLC, using an Agilent mass directed purification system (1260 infinity LC and 6130 single quad MS). For a typical purification, peptides were dissolved in 95/5 water/acetonitrile (0.1% TFA) and passed through a 0.2 μm PTFE syringe filter. The resulting peptide solution was then loaded onto a 9.4 x 250 mm column (Agilent Zorbax 300SB-C3; 5 μm particle size; 300 Å pore size) and purified using a linear gradient of 1 to 61% acetonitrile (0.1% TFA) over 60 min (4 mL/min flow rate). Fractions containing the desired product were pooled and an aliquot taken for LC-MS analysis. The remainder was lyophilized.

For LC-MS characterization: LC-MS data were acquired using an Agilent 6550 quadrupole time-of-flight LC-MS. Samples were run on an Agilent Zorbac 200SB-C3 column (2.1 x 150 mm, 5 μm particle size, 300 Å pore size). Total ion current (TIC) chromatograms were plotted, and mass spectra were integrated over the principal TIC peak, shown below.

Competition fluorescence polarization of HA epitope and analogues:

Solutions of unlabeled peptides (~1 mg/mL each; **Supplementary Table 1**) in 1x PBS was prepared in the presence of 100 nM 12ca5, 1 mg/mL BSA, 0.02% Tween 20 (120 μL). Fluorescent competitor was added (YPYDVPDYAK(FITC)α-CONH2; 28 nM). The resulting solution was diluted serially (20 μL) into 100 nM 12ca5, 28 nM YPYDVPDYAK(FITC)α-CONH2, 1 mg/mL BSA, 0.02% Tween 20 in 1x PBS (80 μL; 5-fold dilutions). The resulting solutions were transferred to a 96 well plate (Greiner Bio-One; Kremsmünster, Austria; polypropylene, flat-bottom, chimney well), kept under foil (RT), and read after 1 h on a Tecan Infinite M1000 plate reader (470 nm excitation; 517 nm detection; 5 nm bandwidth). The concentration of fluorescent 12CA5 was determined based on absorbance at 490 nm, using $\varepsilon = 76{,}900\ M^{-1}cm^{-1}$.

BioLayer Interferometry of 25 nM-affinity 12ca5 ligand:

Lyophilized peptide FDYEDYAEWKK(biotin) (biotinylated on C-term lysine) was dissolved to 1 mg/mL in 1x PBS and diluted 50-fold into 1 mg/mL BSA, 0.02% Tween-20, 1x PBS ('kinetic buffer') for immobilization onto streptavidin Octet biosensors (ForteBio; Menlo Park, CA). Biolayer interferometry (BLI) assays were performed in 96 well plates (GreinerBio-One; Kremsmünster, Austria; polypropylene, flat-bottom, chimney well) using an Octet Red96 System (ForteBio; Menlo Park, CA). Wells were filled with 200 μL of kinetic buffer, peptide solution, or 12ca5 solution (prepared in kinetic buffer). Biotinylated peptide was immobilized onto the streptavidin tip for 120 s. Sensors were then dipped into kinetic buffer for 60 s, 12ca5 solution (1.5 μM, 370 nM, or 90 nM) for 300 s, and finally into kinetic buffer for 600 s. Measurements were carried out at 30 °C.

Preparation of biotinylated 12ca5:

Biotin-$(PEG)_4$-NHS ester (2.0 mg, 3.3 µmol) was weighed into a plastic tube and dissolved in 1.06 mL of DMF ([Biotin-$(PEG)_4$-NHS] = 3.3 mM). Anti-HA antibody (4.71 mg/mL in 1x PBS, 1.06 mL, 33 nmol) was transferred to a plastic tube and to this was added 123 µL of 1M sodium bicarbonate, pH = 8.0. Biotin-$(PEG)_4$-NHS ester (3.3 mM in DMF, 53 µL, 170 nmol) was added dropwise to solution of anti-HA antibody, and reaction was placed on a nutating mixer for 2 h at ambient temperature. Reaction was quenched with addition of 20 mM Tris, 150 mM NaCl, pH = 7.5 (4 mL). Mixture was then filtered through a 0.2 µm PTFE syringe filter, and purified by FPLC (ÄKTA Prime Plus Liquid Chromatography System, GE Healthcare). Concentration of biotinylated 12ca5 was measured by absorption at 280 nm, using a determined extinction coefficient of 2.0×10^5 $M^{-1}cm^{-1}$. Protein was stored at 4 °C and not subjected to freeze-thaw cycles.

Affinity capture of 12ca5-binding peptides—effect of ligand concentration:

Preparation of 12ca5-functionalized magnetic beads:

100 µL portions of MyOne Streptavidin T1 Dynabeads (10 mg/mL; 1 mg; 0.13 nmol IgG binding capacity) were transferred to 1.7 mL plastic centrifuge tubes, and placed in a magnetic separation rack (New England Biolabs, cat# S1506S). The beads were washed 3 times with 1 mg/mL BSA, 0.02% Tween 20, 1x PBS, and then treated with 100 µL portions of biotinylated 12ca5 (1.5 µM; 0.15 nmol). The resulting suspensions were transferred to a rotating vertical mixer, and kept for 15 min at ambient temperature. After this time, the beads were returned to the separating rack, the supernatant was removed, and the beads were washed 4 times with 1 mL each of 1 mg/mL BSA, 0.02% Tween 20, 1x PBS.

Affinity capture:

1 mL solutions containing 1 mg/mL BSA, 0.02% Tween 20, 1x PBS, and either 1 nM/peptide (1 pmol) or 10 pM/peptide (10 fmol) 12ca5 control binders (**Supplementary Table 1**) were prepared in 1.7 mL plastic centrifuge tubes, and chilled on ice for 10 min (the 12ca5 binders were added from mixtures containing 1 µM/peptide or 10 nM/peptide in 6 M guanidine hydrochloride, 200 mM phosphate, pH 7 buffer). The resulting chilled solutions were then added to 1 mg portions of 12ca5-functionalized magnetic beads, and the resulting suspensions were kept on a rotating vertical mixer (1 h, in 4 °C cold room).

Elution:

The centrifuge tubes containing the bead suspensions were transferred to the magnetic separation rack. The beads were isolated, and washed 3 times with 1 mL each of chilled 1x PBS (beads were exposed to buffer for a total of ~6 min). Then, each drained bead aliquot was treated with 2 x 150 µL of 'elution buffer' (6 M guanidine hydrochloride, 200 mM phosphate, pH 7.0 buffer containing 1 fmol/µL Peptide Retention Time Calibration Standard (PRTC; Pierce, cat# 88320; for use as an internal reference in MS-based quantitation)).

Preparation of 'reference' samples:

1 pmol and 10 fmol/peptide 'reference' samples were prepared by dilution of 1 µM/peptide or 10 nM/peptide 12ca5 binder mixture stock solutions (in 6 M guanidine hydrochloride, 200 mM phosphate, pH 7 buffer) into 300 µL of 'elution buffer'. These samples contained the amount of peptide that would be present in elution, if 100% of the peptide were retained by affinity capture.

NanoLC-MS:

5 µL portions of the combined eluates were analyzed by nanoLC-MS, alongside 5 µL portions of 'reference' samples (16.7 fmol/peptide or 167 amol/peptide loading for '1

nM' or '10 pM' conditions, respectively). Analysis was performed on an EASY-nLC 1200 (Thermo Fisher Scientific) nano-liquid chromatography handling system connected to an Orbitrap Fusion Lumos Tribrid Mass Spectrometer (Thermo Fisher Scientific). Samples were run on a PepMap RSLC C18 column (2 µm particle size, 15 cm x 50 µm ID; Thermo Fisher Scientific, P/N ES801). A nanoViper Trap Column (C18, 3 µm particle size, 100 Å pore size, 20 mm x 75 µm ID; Thermo Fisher Scientific, P/N 164946) was used for desalting. The standard nano-LC method was run at 40 °C and a flow rate of 300 nL/min with the following gradient: 1% solvent B in solvent A ramping linearly to 61% B in A over 40 or 60 min, where solvent A = water (0.1% FA), and solvent B = 80% acetonitrile, 20% water (0.1% FA). Positive ion spray voltage was set to 2200 V. Orbitrap detection was used for primary MS, with the following parameters: resolution = 120,000; quadrupole isolation; scan range = 200-1400 m/z; RF lens = 30%; AGC target = 1 x 10^6; maximum injection time = 100 ms; 1 microsan.

Generation of dose-response curve:

A dose-response curve was generated by analyzing 5 µL portions of the 'reference samples', containing 16.7 fmol/peptide, 1.67 fmol/peptide, or 167 amol/peptide. MS detector counts for each peptide were determined from the apex of extracted ion current chromatograms, and plotted vs. sample loading to verify the linearity of response over the sample loading range of interest.

Quantitation of sample recovery:

MS detector counts for each peptide were determined from the apex of extracted ion current chromatograms. Recoveries were taken as the ratio of counts for samples obtained by affinity selection vs. the 'reference' samples. To account for run-to-run variability, these ratios were adjusted based on the counts obtained for internal standard (Peptide Retention Time Calibration Standard).

Affinity capture of 12ca5-binding peptides—effect of capture protocol:

'Direct capture' with 12ca5-functionalized magnetic beads:

Preparation of 12ca5-functionalized magnetic beads was carried out as in **Affinity capture of 12ca5-binding peptides—effect of ligand concentration**. Affinity capture treatments were performed as in **Affinity capture of 12ca5-binding peptides—effect of ligand concentration**, from 1 mL volumes of 1 nM, 100 pM, or 10 pM/peptide mixtures of 12ca5-binding peptides (**Supplementary Table 1**).

'Indirect capture' by treatment with 12ca5-biotin:

1 mL solutions containing 1 mg/mL BSA, 0.02% Tween 20, 1x PBS, and either 1 nM, 100 pM, or 10 pM/peptide 12ca5 control binders (**Supplementary Table 1**) were prepared in 1.7 mL plastic centrifuge tubes. The solutions were chilled on ice for 10 min, 12ca5-biotin was added (100 nM; 100 pmol), and the resulting solutions were kept on a rotating vertical mixer (in 4 °C cold room). After 1 h, 1 mg portions of MyOne Streptavidin T1 Dynabeads (0.13 nmol IgG binding capacity) were added in 100 µL each of 1 mg/mL BSA, 0.02% Tween 20, 1x PBS. The resulting solutions were kept for 15 min (rotating vertical mixer, in 4 °C cold room).

Elution, nanoLC-MS analyses of elution and 'reference' samples, and quantitation were performed as in **Affinity capture of 12ca5-binding peptides—effect of ligand concentration**.

Affinity capture of 12ca5-binding peptides—effect of magnetic bead concentration:

'Direct capture' with 12ca5-functionalized magnetic beads:

100 μL (1 mg; 0.13 nmol IgG binding capacity) or 1 mL (10 mg; 1.3 nmol IgG binding capacity) portions of MyOne Streptavidin T1 Dynabeads (10 mg/mL); were transferred to 1.7 mL plastic centrifuge tubes, and placed in a magnetic separation rack. The beads were washed 3 times with 1 mg/mL BSA, 0.02% Tween 20, 1x PBS, and then treated with 100 μL or 1 mL portions of biotinylated 12ca5 (1.5 μM; 0.15 nmol or 1.5 nmol). The resulting suspensions were transferred to a rotating vertical mixer, and kept for 15 min at ambient temperature. After this time, the beads were returned to the separating rack, the supernatant was removed, and the beads were washed 4 x 1 mL each with 1 mg/mL BSA, 0.02% Tween 20, 1x PBS.

Affinity capture was performed from 1 mL solutions containing 1 mg/mL BSA, 0.02% Tween 20, 1x PBS, and 1 nM/peptide (1 pmol) 12ca5 control binders (**Supplementary Table 1**). Elution, nanoLC-MS analyses of elution and 'reference' samples, and quantitation were performed as in **Affinity capture of 12ca5-binding peptides—effect of ligand concentration**. (Note: raw ion counts were not normalized, as PRTC was absent from the 'reference' samples. Counts for a PRTC ion are shown in **Supplementary Fig. 10** to illustrate the degree of run-to-run variability in MS response.)

'Indirect capture':

1 mL solutions containing 1 mg/mL BSA, 0.02% Tween 20, 1x PBS, and 1 nM/peptide 12ca5 control binders (**Supplementary Table 1**) were prepared in 1.7 mL plastic centrifuge tubes. The solutions were chilled on ice for 10 min, 12ca5-biotin was added to either 100 (100 pmol) or 1 μM (1 nmol), and the resulting solutions were kept on a rotating vertical mixer (in 4 °C cold room). After 1 h, 1 mg (0.13 nmol IgG binding capacity) or 10 mg (1.3 nmol IgG binding capacity) portions of MyOne Streptavidin T1 Dynabeads were added in 100 μL each of 1 mg/mL BSA, 0.02% Tween 20, 1x PBS. The resulting solutions were kept for 15 min (rotating vertical mixer, in 4 °C cold room).

Elution, nanoLC-MS analyses of elution and 'reference' samples, and quantitation were performed as in **Affinity capture of 12ca5-binding peptides—effect of ligand concentration**. (Note the caveat above, under 'direct capture'.)

Affinity capture of 12ca5-binding peptides—effect of ligand concentration, with concentration of eluate:

'Direct' affinity capture was performed as in **Affinity capture of 12ca5-binding peptides—effect of ligand concentration**, from 1 mL solutions containing 1 mg/mL BSA, 0.02% Tween 20, 1x PBS, and either 10 pM/peptide (10 fmol) or 1 pM/peptide (1 fmol) of 12ca5 control binders (**Supplementary Table 1**). The resulting elutions (300 µL each) were concentrated by solid phase extraction using C18 ZipTip® cartridges (0.6 µL, MilliporeSigma, P/N ZTC18S096). This involved: 1) wetting the bonded phase with 80 µL of acetonitrile (0.08% trifluoroacetic acid); 2) equilibration with 3 x 80 µL of water (0.1% trifluoroacetic acid); 3) loading the affinity capture eluate (300 µL); 4) de-salting with 3 x 80 µL of water (0.1% trifluoroacetic acid); and 5) elution with 50 µL of 50/50 water (0.1 % trifluoroacetic acid)/acetonitrile (0.08% trifluoroacetic acid) containing 100 mM guanidine hydrochloride, 3.3 mM phosphate). Elutions were collected in 1.7 mL plastic tubes, and lyophilized to give white pellets that were reconstituted in 6 µL each of water (0.1% formic acid). nLC-MS analysis was performed using 5 µL injections of each sample.

Preparation of 2 x 10^6, 2 x 10^7, and 2 x 10^8-member $(X)_9K$-$CONH_2$ libraries:

Library design: $(X)_9K$-$CONH_2$

SPPS:

2.9 g of 30 μm TentaGel resin (0.26 mmol/g, 0.74 mmol, 2 x 10^8 beads) was transferred to a 100 mL peptide synthesis vessel, swollen in DMF, and then washed with DMF (3x). Fmoc-Rink amide linker (2.0 g, 3.71 mmol, 5 eq) was dissolved in HATU solution (0.38 M in DMF, 8.8 mL, 3.4 mmol), activated with DIEA (1.86 mL, 10.7 mmol) immediately prior to coupling, and added to resin bed. Coupling was performed for 20 min; after this time, resin was washed with DMF (100 mL). Fmoc removal was carried out by treatment of resin with 20% piperidine in DMF (1 x 50 mL flow wash; 2 x 50 mL, 5 min batch treatments). Resin was then washed with DMF (150 mL). Coupling of Fmoc-Lys(Boc)-OH, subsequent Fmoc removal, and DMF washes were performed in the same manner.

At this stage, resin was suspended in DMF (50 mL), and divided evenly among 18 x 10 mL fritted plastic syringes using a 5 mL Eppendorf pipette. Couplings were performed as follows: Fmoc-protected amino acids (0.4 mmol) in HATU solution (0.38M, 980 μL, 0.37 mmol) were activated with DIEA (206 μL, 1.2 mmol). Each of the following amino acid derivatives was added to a single portion of resin (theory: 180 mg resin, 40 μmol): Fmoc-Ala-OH, Fmoc-Asp(OtBu)-OH, Fmoc-Glu(OtBu)-OH, Fmoc-Phe-OH, Fmoc-Gly-OH, Fmoc-His(Boc)-OH, Fmoc-Lys(Boc)-OH, Fmoc-Leu-OH, Fmoc-Met-OH, Fmoc-Asn(Trt)-OH, Fmoc-Pro-OH, Fmoc-Gln(Trt)-OH, Fmoc-Arg(Pbf)-OH, Fmoc-Ser(tBu)-OH, Fmoc-Thr(tBu)-OH, Fmoc-Val-OH, Fmoc-Trp(Boc)-OH, and Fmoc-Tyr(tBu)-OH. After coupling for 20 min, resins were washed with DMF (~10 mL ea.), poured back into a 100 mL synthesis vessel, and washed with DMF (100 mL). Fmoc removal was carried out by treatment of resin with 20% piperidine in DMF (1 x 50 mL flow wash; 2 x 50 mL, 5 min batch treatments), and resin was washed with DMF (150 mL). Nine cycles of split-and-pool synthesis were performed using this procedure.

Following removal of the N-terminal Fmoc group, the resin was washed with DMF (150 mL), and a small portion was transferred to a plastic fritted syringe, washed with DCM (~ 10 mL), and dried under reduced pressure. 1.0 mg of resin was weighed into a plastic tube (theory: 4.7 x 10^4 beads) and set aside for later characterization (described in **Characterization of 2 x 10^6, 2 x 10^7, and 2 x 10^8-member $(X)_9K$-$CONH_2$ libraries).** The remainder was resuspended in DMF and pooled back with the bulk of the library.

Portioning:

Resin was suspended in DMF (~50 mL) and divided evenly among 11 x 10 mL fritted plastic syringes. One of these portions of resin was held aside, and the remainder pooled back together (theory: 1.8 x 10^8 beads). The portion that was held aside was further divided evenly among 11 x 10 mL fritted plastic syringes. One of these portions was in turn held aside (theory: 1.7 x 10^6 beads), and the remainder was pooled back together (theory: 1.7 x 10^7 beads). These three portions of resin represent approximate 2 x

10^8, 2 x 10^7, and 2 x 10^6-member libraries. Resins were each washed with DCM (~50 mL) in fritted plastic syringes and dried under reduced pressure.

Cleavage from resin:

Libraries were globally deprotected and cleaved from resin by treatment of dry resin with a solution of 94% (v/v) TFA, 2.5% (v/v) ethanedithiol, 2.5% (v/v) water, and 1.0% (v/v) triisopropylsilane, for 3 h at ambient temperature. TFA was then evaporated under a stream of nitrogen, and crude peptide was precipitated by addition of cold diethyl ether. Precipitated peptide was triturated (3x) with cold diethyl ether, dissolved in 30/70 water/acetonitrile (0.1% TFA), passed through a 0.2 μm PTFE syringe filter, and lyophilized.

Solid phase extraction:

The 2 x 10^8-member library (200 mg) was dissolved in 20 mL of 95/5 water/acetonitrile (0.1% TFA); the entirety of the isolated 2 x 10^7 and 2 x 10^6-member libraries were each dissolved in 5 mL of 95/5 water/acetonitrile (0.1% TFA). Libraries were purified over Bond Elut C18 cartridges (1 g bed mass, 40 μm particle size, 6 mL volume; Agilent, P/N 12256130) as follows: cartridges were first conditioned with methanol (~20 mL), and then equilibrated with 99/1 water/acetonitrile (0.1% TFA) (~20 mL). Sample was then loaded, and cartridges were washed with 99/1 water/acetonitrile (0.1% TFA) (~20 mL). Sample was eluted with 30/70 water/acetonitrile (0.1% TFA) (~20 mL). Elutions from each library were collected and lyophilized.

Preparation of stock solutions:

Lyophilized powder of 2 x 10^8-member library (106 mg) was dissolved in DMF (1.08 mL) and then diluted with 1x PBS (9.76 mL) to bring the final concentration to ~8 mM total peptide (~40 pM/member). Lyophilized powder of 2 x 10^7-member library (39 mg) was dissolved in DMF (3.96 mL) and diluted with 1x PBS (35.6 mL) to bring the final concentration to ~0.8 mM total peptide (~40 pM/member). Lyophilized powder of 2 x 10^6-member library was dissolved in DMF (1.09 mL) and diluted with 1x PBS (9.83 mL) to bring the final concentration to ~0.8 mM total peptide (~400 pM/member). Stock solutions were aliquotted out and stored at -80 °C. Aliquots were thawed on ice prior to use.

Characterization of 2 x 10^6, 2 x 10^7, and 2 x 10^8-member $(X)_9K-CONH_2$ libraries:

Sample preparation:

A 1.0 mg aliquot of library resin (from **Preparation of 2 x 10^6, 2 x 10^7, and 2 x 10^8-member $(X)_9K-CONH_2$ libraries**) was suspended in 1.0 mL of Milli-Q water and sonicated to achieve a homogenous suspension (theory: 4.7 x 10^4 beads/mL). A 20 µL aliquot (theory: 940 beads; 4 pmol/peptide) was transferred to a plastic tube, spun down, and supernatant removed. Beads were then subjected to treatment with 100 µL of 94% (v/v) TFA, 2.5% (v/v) ethanedithiol, 2.5% (v/v) water, and 1.0% (v/v) triisopropylsilane, for 10 min in a 60 °C water bath. TFA was then evaporated under a stream of nitrogen, and cleaved peptide was resuspended in Milli-Q water (0.1% TFA). Sample was purified over a C18 ZipTip® (0.6 µL, MilliporeSigma, P/N ZTC18S096), eluted in 30/70 water/acetonitrile (0.1% TFA), and lyophilized. Powder was resuspended in 20 µL of Milli-Q water (0.1% FA), and 0.5 µL (~100 fmol/peptide) was submitted for nLC-MS/MS analysis.

NanoLC-MS/MS analysis:

Details of the columns and instruments used for analysis are provided in **Affinity capture of 12ca5-binding peptides—effect of ligand concentration**. The standard nano-LC method was run at 40 °C and a flow rate of 300 nL/min with the following gradient: 1% solvent B in solvent A ramping linearly to 41% B in A over 120 min, where solvent A = water (0.1% FA), and solvent B = 80% acetonitrile, 20% water (0.1% FA). Positive ion spray voltage was set to 2200 V. Orbitrap detection was used for primary MS with the following parameters: resolution = 120,000; quadrupole isolation; scan range = 200-1400 m/z; RF lens = 30%; AGC target = 1 x 10^6; maximum injection time = 100 ms; 1 microsan.

Acquisition of secondary MS spectra was done in a data-dependent manner: dynamic exclusion was employed such that a precursor was excluded for 30 s if it was detected four or more times within 30 s (mass tolerance: 10.00 ppm); monoisotopic precursor selection used to select for peptides; intensity threshold was set to 5 x 10^4; charge states 2-10 were selected; and precursor selection range was set to 200-1400 m/z. The top 15 most intense precursors that met the preceding criteria were subjected to subsequent fragmentation.

Three fragmentation modes – collision-induced dissociation (CID), higher-energy collisional dissociation (HCD), and electron-transfer/higher-energy collisional dissociation (EThcD) – were used for acquisition of secondary MS spectra. Only precursors with charge states 3 and above were subjected to all three fragmentation modes; precursors with charge states of 2 were subjected to CID and HCD only. For all three modes, detection was performed in the Orbitrap (resolution = 30,000; quadrupole isolation; isolation window = 1.3 m/z; AGC target = 2 x 10^4; maximum injection time = 100 ms; 1 microscan). For CID, a collision energy of 30% was used. For HCD, a collision energy of 25% was used. For EthcD, a supplemental activation collision energy of 25% was used.

De novo peptide sequencing:

De novo peptide sequencing was performed by processing .raw files obtained from Orbitrap analysis using PEAKS Studio (version 8.5) from Bioinformatics Solutions Inc. (ON, Canada). HCD and CID scans were merged within a 0.2 minute and 0.02 Da window, mass precursor correction was used, and primary mass filtration was employed as appropriate. Auto *de novo* sequencing was performed using a 15 ppm precursor mass error and 0.02 Da fragment mass error, and with the following modifications: fixed C-term amidation (-0.98 Da) on lysine, and variable oxidation on methionine (+15.99 Da). 15 candidate sequences were obtained for each preprocessed scan. Post-*de novo* data analysis was performed as described in Vinogradov, A. V. *et. al*. Library design-facilitated high-throughput sequencing of synthetic peptide libraries. *ACS Comb. Sci.* **19** 694-701 (2017).[3]

Affinity selections of 2 x 10^6, 2 x 10^7, and 2 x 10^8-member libraries against 12ca5:

Preparation of 12ca5-functionalized magnetic beads:

MyOne Streptavidin T1 Dynabeads (300 µL of 10 mg/mL stock) were transferred to 1.7 mL plastic centrifuge tubes, and placed in a magnetic separation rack. Beads were washed 3 x 1 mL w/ 10% FBS, 0.02% Tween 20, 1x PBS, and then treated with 300 µL of biotinylated 12ca5 (1.5 µM; 0.45 nmol). The resulting suspensions were transferred to a rotating vertical mixer and allowed to incubate for 1 h at 4°C. After this time, the beads were returned to the separating rack, the supernatant was removed, and the beads were washed 3 x 1 mL w/ 10% FBS, 0.02% Tween 20, 1x PBS. Beads were resuspended in 300 µL of 10% FBS, 0.02% Tween 20, 1x PBS.

Affinity capture:

Library (10 fmol/member) was incubated with 100 µL (1 mg) portions of protein-immobilized magnetic beads (prepared above) in the presence of 10% FBS, 1x PBS (final volume: 1 mL) on a rotating mixer for 1 h at 4 °C. Final conditions: 1 mg/mL magnetic beads, 10 pM/member library.

Elution:

The centrifuge tubes containing the bead suspensions were transferred to the magnetic separation rack. The beads were washed 3 x 1 mL w/ 1x PBS. Bound peptides were eluted with 2 x 100 µL 6M guanidine hydrochloride, 200 mM phosphate, pH 6.8. Eluates were concentrated via C18 ZipTip® pipette tips (as described in **Affinity capture of 12ca5-binding peptides—effect of ligand concentration, with concentration of eluate**) and lyophilized.

NanoLC-MS/MS:

Powders were resuspended in 6 µL water (0.1% formic acid), and 5 µL submitted for nLC-MS/MS analysis. Analysis was performed as described in **Characterization of 2 x 10^6, 2 x 10^7, and 2 x 10^8-member $(X)_9K$-$CONH_2$ libraries**.

SPPS of HA epitope Ala scan mutants:

Peptides were synthesized on a fully automated fast-flow peptide synthesizer as described in **Solid phase peptide synthesis (SPPS) of anti-hemagglutinin (HA) epitope and analogues**. Concomitant side chain deprotection and cleavage from resin, as well as HPLC purification and LC-MS analysis, were also carried out as described in **Solid phase peptide synthesis (SPPS) of anti-hemagglutinin (HA) epitope and analogues**.

Competition fluorescence polarization of HA epitope Ala scan mutants:

Competition fluorescence polarization experiments were carried out as described in **Competition fluorescence polarization of HA epitope and analogues**.

Investigation of enrichment vs. sample loading:

MyOne Streptavidin T1 Dynabeads (3 mg) were washed 3 x 1 mL w/ 10% FBS/1x PBS. Beads were then incubated with biotinylated 12ca5 (0.13 nmol of protein per 1 mg of magnetic beads) for 1 h at 4°C, and washed 3 x 1 mL w/ 10% FBS/1x PBS. For each replicate (3 total): library (200 fmol/member) was incubated with 1 mg protein-immobilized magnetic beads in the presence of 10% FBS/1x PBS (final volume: 1 mL) for 1 h at 4°C. Final screening conditions: 100 nM protein, 1 mg/mL magnetic beads, 200 pM/member library. Beads were washed 3 x 1 mL w/ 1x PBS. Bound peptides were eluted with 2 x 100 μL 6M Guan•HCl, 200 mM phosphate, pH 6.8. Eluates were concentrated via ZipTip and lyophilized. Powders were resuspended in 13.3 μL of water (0.1% FA), 6.3 μL was removed for later analysis (theory: 15 fmol library member/μL). Remaining 7 μL were diluted into 14 μL of water (0.1% FA), and 10 μL was removed for later analysis (theory: 5 fmol library member/μL). Finally, remaining 11 μL were diluted into 22 μL of water (0.1% FA), and 10 μL was removed for later analysis (theory: 1.7 fmol library member/μL). 5 μL of each dilution were submitted for nLC-MS/MS analysis, giving theoretical injection amounts (assuming 100% recovery of peptide) of 8.3 fmol, 25 fmol, and 75 fmol library member, respectively.

Preparation of a 1 x 10^9-member $(X)_9K$-$CONH_2$ library:

Library design: $(X)_9K$-$CONH_2$

SPPS:

5.4 g of 20 µm TentaGel resin (0.26 mmol/g, 1.4 mmol, 1.3 x 10^9 beads) was transferred to a 100 mL peptide synthesis vessel, swollen in DMF, and then washed with DMF (3x). Fmoc-Rink amide linker (3.8 g, 7.0 mmol, 5 eq) was dissolved in HATU solution (0.38 M in DMF, 16.7 mL, 6.4 mmol), activated with DIEA (3.5 mL, 20 mmol) immediately prior to coupling, and added to resin bed. Coupling was performed for 20 min; after this time, resin was washed with DMF (100 mL). Fmoc removal was carried out by treatment of resin with 20% piperidine in DMF (1 x 50 mL flow wash; 2 x 50 mL, 5 min batch treatments). Resin was then washed with DMF (150 mL). Coupling of Fmoc-Lys(Boc)-OH, subsequent Fmoc removal, and DMF washes were performed in the same manner.

At this stage, resin was suspended in DMF (50 mL), and divided evenly among 18 x 10 mL fritted plastic syringes using a 5 mL Eppendorf pipette. Couplings were performed as follows: Fmoc-protected amino acids (0.8 mmol) in HATU solution (0.38M, 1.86 mL, 0.71 mmol) were activated with DIEA (391 µL, 2.3 mmol). Each of the amino acid derivatives listed in **Preparation of 2 x 10^6, 2 x 10^7, and 2 x 10^8-member $(X)_9K$-$CONH_2$ libraries** was added to a single portion of resin (theory: ~330 mg resin, 80 µmol). Couplings were performed for 20 min. Remainder of split-and-pool synthesis (nine rounds total) was completed according to the procedure outlined in **Preparation of 2 x 10^6, 2 x 10^7, and 2 x 10^8-member $(X)_9K$-$CONH_2$ libraries**.

Following removal of the N-terminal Fmoc group, the resin was washed with DMF (150 mL), and a small portion was transferred to a plastic fritted syringe, washed with DCM (~ 10 mL), and dried under reduced pressure. 1.0 mg of resin was weighed into a plastic tube (theory: 1.6 x 10^5 beads) and set aside for later characterization (described in **Characterization of a 1 x 10^9-member $(X)_9K$-$CONH_2$ library).** The remainder was resuspended in DMF and pooled back with the bulk of the library.

Portioning:

Resin was suspended in DMF (~50 mL) and divided evenly among 11 x 10 mL fritted plastic syringes. One of these portions of resin was held aside (theory: 1.2 x 10^8 beads), and the remainder pooled back together (theory: 1.2 x 10^9 beads). These two portions of resin represent approximate 1 x 10^9 and 1 x 10^8-member libraries. Resins were each washed with DCM (~50 mL) in fritted plastic syringes and dried under reduced pressure.

Cleavage from resin and solid phase extraction:

Libraries were globally deprotected and cleaved from resin as described in **Preparation of 2 x 10^6, 2 x 10^7, and 2 x 10^8-member $(X)_9K$-$CONH_2$ libraries**. Crude, lyophilized powders were resuspended in 95/5 water/acetonitrile (0.1% TFA), and

purified over Supelclean™ LC-18 SPE cartridges (2 g bed mass, 45 μm particle size, 12 mL; Millipore Sigma, P/N 57117). Procedure is described in **Preparation of 2 x 10^6, 2 x 10^7, and 2 x 10^8-member $(X)_9K$-$CONH_2$ libraries**.

Preparation of stock solutions:

Lyophilized powder of 10^9-member library (127 mg) was dissolved in DMF (1.3 mL) and then diluted with 1x PBS (11.7 mL) to a final concentration of 8 mM total peptide (~6 pM/member). Lyophilized powder of 10^8-member library (110 mg) was dissolved in DMF (1.2 mL) and diluted with 1x PBS (10.1 mL) to a final concentration of 8 mM total peptide (~60 pM/member). Stock solutions were aliquotted out and stored at -80 °C. Aliquots were thawed on ice prior to use.

Characterization of a 1 x 10^9-member $(X)_9$K-$CONH_2$ library

Sample preparation:

A 1.0 mg aliquot of library resin (from **Preparation of a 1 x 10^9-member $(X)_9$K-$CONH_2$ library**) was suspended in 1.0 mL of Milli-Q water and sonicated to achieve a homogenous suspension (theory: 1.6 x 10^5 beads/mL). A 5 µL aliquot (theory: 805 beads; 1 pmol/peptide) was transferred to a plastic tube, spun down, and supernatant removed. Beads were then subjected to treatment with 94% (v/v) TFA, 2.5% (v/v) ethanedithiol, 2.5% (v/v) water, and 1.0% (v/v) triisopropylsilane, for 10 min in a 60 °C water bath. TFA was then evaporated under a stream of nitrogen, and cleaved peptide was resuspended in Milli-Q water (0.1% TFA). Sample was purified over a C18 ZipTip® (0.6 µL, MilliporeSigma, P/N ZTC18S096), eluted in 30/70 water/acetonitrile (0.1% TFA), and lyophilized. Powder was resuspended in 13 µL of Milli-Q water (0.1% FA), and 1 µL (~100 fmol/peptide) was submitted for nLC-MS/MS analysis.

NanoLC-MS/MS analysis and *de novo* peptide sequencing:

Analysis and *de novo* sequencing was performed as described in **Characterization of 2 x 10^6, 2 x 10^7, and 2 x 10^8-member $(X)_9$K-$CONH_2$ libraries**.

Affinity selections of 10^8 and 10^9-member libraries against 12ca5—effect of library diversity:

Preparation of 12ca5-functionalized magnetic beads:

MyOne Streptavidin T1 Dynabeads were functionalized with biotinylated 12ca5 as described in **Affinity selections of 2 x 10^6, 2 x 10^7, and 2 x 10^8-member libraries against 12ca5.**

Affinity capture:

10^9-member library (2 fmol/member), 10^8-member portion (10 fmol/member), or 10^8-member library from **Preparation of 2 x 10^6, 2 x 10^7, and 2 x 10^8-member $(X)_9K$-$CONH_2$ libraries** (2 fmol/member) was incubated with 100 µL (1 mg) portions of protein-immobilized magnetic beads in the presence of 10% FBS, 1x PBS (final volume: 1 mL) on a rotating mixer for 1 h at 4 °C. Final conditions: 1 mg/mL magnetic beads, 2 pM/member or 10 pM/member library.

Elution and nanoLC-MS/MS:

Bound peptides were eluted as described in **Affinity selections of 2 x 10^6, 2 x 10^7, and 2 x 10^8-member libraries against 12ca5**. NanoLC-MS/MS analysis was performed as described in **Characterization of 2 x 10^6, 2 x 10^7, and 2 x 10^8-member $(X)_9K$-$CONH_2$ libraries** and **Affinity selections of 2 x 10^6, 2 x 10^7, and 2 x 10^8-member libraries against 12ca5**.

Affinity selections of 10^8 and 10^9-member libraries against 12ca5—effect of increased starting material of library:

Preparation of 12ca5-functionalized magnetic beads:

MyOne Streptavidin T1 Dynabeads were functionalized with biotinylated 12ca5 as described in **Affinity selections of 2 x 10^6, 2 x 10^7, and 2 x 10^8-member libraries against 12ca5.**

Affinity capture:

10^9-member library (1 fmol/member, 5 fmol/member, or 3 x 4 fmol/member (= 12 fmol/member total)) was incubated with 100 µL (1 mg) portions of protein-immobilized magnetic beads in the presence of 10% FBS, 1x PBS (final volume: 1 mL) on a rotating mixer for 1 h at 4 °C. Final conditions: 1 mg/mL magnetic beads; variable concentration of library members.

Elution and nanoLC-MS/MS:

Bound peptides were eluted as described in **Affinity selections of 2 x 10^6, 2 x 10^7, and 2 x 10^8-member libraries against 12ca5**. For the 12 fmol/member condition, eluates from each 4 fmol/member selection were combined and concentrated as described in **Affinity capture of 12ca5-binding peptides—effect of ligand concentration, with concentration of eluate**. NanoLC-MS/MS analysis was performed as described in **Characterization of 2 x 10^6, 2 x 10^7, and 2 x 10^8-member $(X)_9K\text{-}CONH_2$ libraries** and **Affinity selections of 2 x 10^6, 2 x 10^7, and 2 x 10^8-member libraries against 12ca5**.

Affinity selections of 10^8 and 10^9-member libraries against 12ca5—effect of increased starting material of selection target:

Preparation of 12ca5-functionalized magnetic beads:

MyOne Streptavidin T1 Dynabeads (1.1 mL of 10 mg/mL stock) were transferred to a 1.7 mL plastic centrifuge tube, and placed in a magnetic separation rack. Beads were washed 3 x 1 mL w/ 10% FBS, 1x PBS, and then treated with 1.0 mL of biotinylated 12ca5 (1.7 µM; 1.7 nmol). The resulting suspensions were transferred to a rotating vertical mixer and allowed to incubate for 1 h at 4°C. After this time, the beads were returned to the separating rack, the supernatant was removed, and the beads were washed 3 x 1 mL w/ 10% FBS, 1x PBS. Beads were resuspended in 300 µL of 10% FBS, 1x PBS.

Affinity capture:

10^9-member library (2 fmol/member) was incubated with either 100 µL (1 mg; 0.13 nmol IgG binding capacity) of 12ca5-immobilized magnetic beads, or 1 mL (10 mg; 1.3 nmol IgG binding capacity) of 12ca5-immobilized magnetic beads, in the presence of 10% FBS, 1x PBS (final volume: 1 mL) on a rotating mixer for 1 h at 4 °C. Final conditions: 1 mg/mL magnetic beads; 2 pM/member library.

Elution and nanoLC-MS/MS:

Bound peptides were eluted as described in **Affinity selections of 2 x 10^6, 2 x 10^7, and 2 x 10^8-member libraries against 12ca5**. NanoLC-MS/MS analysis was performed as described in **Characterization of 2 x 10^6, 2 x 10^7, and 2 x 10^8-member $(X)_9K$-$CONH_2$ libraries** and **Affinity selections of 2 x 10^6, 2 x 10^7, and 2 x 10^8-member libraries against 12ca5**.

Affinity selections of 10^8 and 10^9-member libraries against 12ca5—effect of exogenous competitor on selections from a 10^8-member library:

Preparation of 12ca5-functionalized magnetic beads:

MyOne Streptavidin T1 Dynabeads were functionalized with biotinylated 12ca5 as described in **Affinity selections of 2 x 10^6, 2 x 10^7, and 2 x 10^8-member libraries against 12ca5.**

Affinity capture:

10^8-member library (10 fmol/member) was incubated with 100 μL (1 mg; 0.13 nmol IgG binding capacity) of 12ca5-immobilized magnetic beads, in the presence of either 1 nM, 10 nM, or 100 nM HA epitope, or 100 nM, 1 μM, or 10 μM Gypyeydwe peptide, and 10% FBS, 1x PBS (final volume: 1 mL) on a rotating mixer for 1 h at 4 °C. Final conditions: 1 mg/mL magnetic beads; 10 pM/member library.

Elution and nanoLC-MS/MS:

Bound peptides were eluted as described in **Affinity selections of 2 x 10^6, 2 x 10^7, and 2 x 10^8-member libraries against 12ca5**. NanoLC-MS/MS analysis was performed as described in **Characterization of 2 x 10^6, 2 x 10^7, and 2 x 10^8-member $(X)_9$K-$CONH_2$ libraries** and **Affinity selections of 2 x 10^6, 2 x 10^7, and 2 x 10^8-member libraries against 12ca5**.

Side-by-side selections against 12a5 and human polyclonal IgG_1:

Preparation of 12ca5-functionalized and human IgG_1-functionalized magnetic beads:

MyOne Streptavidin T1 Dynabeads (2 x 300 µL of 10 mg/mL stock) were transferred to 1.7 mL plastic centrifuge tubes, and placed in a magnetic separation rack. Beads were washed 3 x 1 mL w/ 10% FBS, 0.02% Tween 20, 1x PBS, and then treated with 300 µL of biotinylated 12ca5 (1.5 µM; 0.45 nmol) or 120 µL of biotinylated human polyclonal IgG_1 (3.8 µM; 0.45 nmol; diluted to 300 µL with 10% FBS, 0.02% Tween 20, 1x PBS). The resulting suspensions were transferred to a rotating vertical mixer and allowed to incubate for 1 h at 4°C. After this time, the beads were returned to the separating rack, the supernatant was removed, and the beads were washed 3 x 1 mL w/ 10% FBS, 0.02% Tween 20, 1x PBS. Beads were resuspended in 300 µL of 10% FBS, 0.02% Tween 20, 1x PBS.

Affinity capture:

Library (10 fmol/member) was incubated with 100 µL (1 mg) portions of protein-immobilized magnetic beads (prepared above) in the presence of 10% FBS, 1x PBS (final volume: 1 mL) on a rotating mixer for 1 h at 4 °C. Final conditions: 1 mg/mL magnetic beads, 10 pM/member library.

Elution and nanoLC-MS/MS:

Bound peptides were eluted as described in **Affinity selections of 2 x 10^6, 2 x 10^7, and 2 x 10^8-member libraries against 12ca5**. NanoLC-MS/MS analysis was performed as described in **Characterization of 2 x 10^6, 2 x 10^7, and 2 x 10^8-member $(X)_9K$-$CONH_2$ libraries** and **Affinity selections of 2 x 10^6, 2 x 10^7, and 2 x 10^8-member libraries against 12ca5**.

Preparation of a 1 x 10^9-member $(X)_{12}K-CONH_2$ library:

Library design: $(X)_{12}K-CONH_2$

SPPS:

4.2 g of 20 µm TentaGel resin (0.26 mmol/g, 1.1 mmol, 1.0 x 10^9 beads) was transferred to a 100 mL peptide synthesis vessel, swollen in DMF, and then washed with DMF (3x). Fmoc-Rink amide linker (2.9 g, 5.4 mmol, 5 eq) was dissolved in HATU solution (0.38 M in DMF, 12.9 mL, 4.9 mmol), activated with DIEA (2.7 mL, 16 mmol) immediately prior to coupling, and added to resin bed. Coupling was performed for 20 min; after this time, resin was washed with DMF (100 mL). Fmoc removal was carried out by treatment of resin with 20% piperidine in DMF (1 x 50 mL flow wash; 2 x 50 mL, 5 min batch treatments). Resin was then washed with DMF (150 mL). Coupling of Fmoc-Lys(Boc)-OH, subsequent Fmoc removal, and DMF washes were performed in the same manner.

At this stage, resin was suspended in DMF (50 mL), and divided evenly among 18 x 10 mL fritted plastic syringes using a 5 mL Eppendorf pipette. Couplings were performed as follows: Fmoc-protected amino acids (0.6 mmol) in HATU solution (0.38M, 1.4 mL, 0.54 mmol) were activated with DIEA (300 µL, 1.7 mmol). Each of the amino acid derivatives listed in **Preparation of 2 x 10^6, 2 x 10^7, and 2 x 10^8-member $(X)_9K-CONH_2$ libraries** was added to a single portion of resin (theory: ~260 mg resin, 60 µmol). Couplings were performed for 20 min. Remainder of split-and-pool synthesis (twelve rounds total) was completed according to the procedure outlined in **Preparation of 2 x 10^6, 2 x 10^7, and 2 x 10^8-member $(X)_9K-CONH_2$ libraries**.

Portioning:

Following removal of the N-terminal Fmoc group, the resin was washed with DMF (150 mL), then suspended in DMF (~ 50 mL) and divided evenly among 5 x 20 mL fritted plastic syringes. Four of these portions (theory: 2 x 10^8 beads each) were washed with DCM (3x) and dried under reduced pressure. The fifth portion was further divided among 11 x 10 mL fritted plastic syringes. Ten of these portions were recombined. The recombined beads, along with the remaining portion (theory: 1.8 x 10^7 beads), were washed with DCM (3x) and dried under reduced pressure. 1.0 mg of dried resin was weighed into a plastic tube (theory: 1.4 x 10^5 beads) and set aside for later characterization (described in **Characterization of a 1 x 10^9-member $(X)_{12}K-CONH_2$ library**).

Cleavage from resin and solid phase extraction:

Libraries were globally deprotected and cleaved from resin as described in **Preparation of 2 x 10^6, 2 x 10^7, and 2 x 10^8-member $(X)_9K-CONH_2$ libraries**. Crude, lyophilized powders were resuspended in 95/5 water/acetonitrile (0.1% TFA), and purified over Supelclean™ LC-18 SPE cartridges (2 g bed mass, 45 µm particle size, 12

mL; Millipore Sigma, P/N 57117). Procedure is described in **Preparation of 2 x 10^6, 2 x 10^7, and 2 x 10^8-member $(X)_9K$-$CONH_2$ libraries**.

Preparation of stock solutions:

Lyophilized powders of 2 x 10^8-member libraries were each dissolved first in DMF and then diluted with 1x PBS to a final library concentration of 8 mM (~40 pM/member), and a final DMF concentration of 10% (v/v). Lyophilized powder of 2 x 10^7-member library was similarly first dissolved in DMF, and then diluted with 1x PBS to a final library concentration of 7 mM (~400 pM/member), and a final DMF concentration of 10% (v/v). Stock solutions were aliquotted out and stored at -80 °C. Aliquots were thawed on ice prior to use.

Characterization of a 1 x 10^9-member $(X)_{12}$K-$CONH_2$ library:

Sample preparation:

A 1.0 mg aliquot of library resin (from **Preparation of a 1 x 10^9-member $(X)_{12}$K-$CONH_2$ library**) was suspended in 1.0 mL of Milli-Q water and sonicated to achieve a homogenous suspension (theory: 1.4 x 10^5 beads/mL). A 4 µL aliquot (theory: 559 beads; 1 pmol/peptide) was transferred to a plastic tube, spun down, and supernatant removed. Beads were then subjected to treatment with 94% (v/v) TFA, 2.5% (v/v) ethanedithiol, 2.5% (v/v) water, and 1.0% (v/v) triisopropylsilane, for 10 min in a 60 °C water bath. TFA was then evaporated under a stream of nitrogen, and cleaved peptide was resuspended in Milli-Q water (0.1% TFA). Sample was purified over a C18 ZipTip® (0.6 µL, MilliporeSigma, P/N ZTC18S096), eluted in 30/70 water/acetonitrile (0.1% TFA), and lyophilized. Powder was resuspended in 34 µL of Milli-Q water (0.1% FA), and 1 µL (~30 fmol/peptide) was submitted for nLC-MS/MS analysis.

NanoLC-MS/MS analysis and *de novo* peptide sequencing:

Analysis and *de novo* sequencing was performed as described in **Characterization of 2 x 10^6, 2 x 10^7, and 2 x 10^8-member $(X)_9$K-$CONH_2$ libraries**.

Preparation of synthetic (25-109)MDM2 K36(biotin):

The N-terminal domain of MDM2 (residues 25-109; sequence shown in **Supplementary Fig. 28**) was synthesized on a 0.03 mmol scale on H-Rink amide-ChemMatrix resin (0.18 mmol/g) via automated fast flow synthesis[4].

A biotin was site-specifically incorporated as follows: Fmoc-L-Lys(alloc)-OH was used for coupling of Lys36 during SPPS. Following main chain elaboration, the N-terminal amino group was Boc-protected by first washing the resin 3 times with DCM, then adding to the resin a solution of di-tert-butyl dicarbonate (40 eq, 400 mM) and DIEA (40 eq) in DCM. Coupling was allowed to proceed for 30 min. At this time, resin was washed 5 times with DCM and coupling was repeated as described. To remove the Alloc group on Lys36, resin was treated with a solution of tetrakis(triphenylphosphine)palladium(0) (2 eq, 20 mM) and phenylsilane (80 eq) in DCM. After 30 min, reaction mixture was drained and deprotection was repeated as described. At this time, resin was washed 5 times with DCM, then 5 times with DMF. To a solution of biotin-(PEG)4-propionic acid (15 eq, 0.42 M) and HATU (13.5 eq, 0.38 M) in DMF was added DIEA (45 eq), and solution was then added to the resin bed and allowed to react for 3 h. At this time, resin was washed 5 times with DMF, 5 times with DCM, and dried under reduced pressure.

LC-MS characterization of HPLC-purified (25-109)MDM2 K36(biotin) was performed as described in **Solid phase peptide synthesis (SPPS) of anti-hemagglutinin (HA) epitope and analogues**.

Affinity selections against MDM2—multi-pot selections of five (2 x 10^8)-member libraries:

Procedure for each selection (five in total), conducted side by side against 12ca5 as a control, is outlined below:

Preparation of MDM2-functionalized and 12ca5-functionalized magnetic beads:

MyOne Streptavidin T1 Dynabeads (2 x 300 µL of 10 mg/mL stock) were transferred to 1.7 mL plastic centrifuge tubes, and placed in a magnetic separation rack. Beads were washed 3 x 1 mL w/ 10% FBS, 0.02% Tween 20, 1x PBS, and then treated with 115 µL of refolded, biotinylated (25-109)MDM2 (10.8 µM; 1.2 nmol; diluted to 300 µL with 10% FBS, 0.02% Tween 20, 1x PBS) or 300 µL of biotinylated 12ca5 (1.5 µM; 0.45 nmol). The resulting suspensions were transferred to a rotating vertical mixer and allowed to incubate for 1 h at 4°C. After this time, the beads were returned to the separating rack, the supernatant was removed, and the beads were washed 3 x 1 mL w/ 10% FBS, 0.02% Tween 20, 1x PBS. Beads were resuspended in 300 µL of 10% FBS, 0.02% Tween 20, 1x PBS.

Affinity capture:

Library (10 fmol/member) was incubated with 100 µL (1 mg) portions of protein-immobilized magnetic beads (prepared above) in the presence of 10% FBS, 1x PBS (final volume: 1 mL) on a rotating mixer for 1 h at 4 °C. Final conditions: 1 mg/mL magnetic beads, 10 pM/member library.

Elution and nanoLC-MS/MS:

Bound peptides were eluted as described in **Affinity selections of 2 x 10^6, 2 x 10^7, and 2 x 10^8-member libraries against 12ca5**. NanoLC-MS/MS analysis was performed as described in **Characterization of 2 x 10^6, 2 x 10^7, and 2 x 10^8-member $(X)_9K$-$CONH_2$ libraries** and **Affinity selections of 2 x 10^6, 2 x 10^7, and 2 x 10^8-member libraries against 12ca5**.

Affinity selections against MDM2—one-pot selections of 2 x 10^7 and 1 x 10^9-member libraries:

Preparation of MDM2-functionalized and 12ca5-functionalized magnetic beads:

Magnetic beads were prepared as described in **Affinity selections against MDM2—multi-pot selections of five (2 x 10^8)-member libraries**.

Affinity capture:

For 2 x 10^7-member library:

Library (10 fmol/member) was incubated with 100 μL (1 mg) portions of protein-immobilized magnetic beads in the presence of 10% FBS, 1x PBS (final volume: 1 mL) on a rotating mixer for 1 h at 4 °C. Final conditions: 1 mg/mL magnetic beads, 10 pM/member library.

For 1 x 10^9-member library:

Library (10 fmol/member) was incubated with 100 μL (1 mg) portions of protein-immobilized magnetic beads in the presence of 10% FBS, 1x PBS (final volume: 1.5 mL) on a rotating mixer for 1 h at 4 °C. Final conditions: 0.7 mg/mL magnetic beads, 7 pM/member library.

Elution and nanoLC-MS/MS:

Bound peptides were eluted as described in **Affinity selections of 2 x 10^6, 2 x 10^7, and 2 x 10^8-member libraries against 12ca5**. NanoLC-MS/MS analysis was performed as described in **Characterization of 2 x 10^6, 2 x 10^7, and 2 x 10^8-member $(X)_9$K-$CONH_2$ libraries** and **Affinity selections of 2 x 10^6, 2 x 10^7, and 2 x 10^8-member libraries against 12ca5**.

Expression of 14-3-3γ:

Full-length human 14-3-3γ was expressed by transforming pROEX HTb plasmid, containing a His6-tagged 14-3-3γFL gene and ampicillin resistance gene, to Rosetta(DE3) Escherichia coli cells (Novagen). Cells were grown at 37 °C, with 0.1 mg/mL ampicillin, and protein expression was induced using 0.4 µM IPTG and 1 mM $MgCl_2$ and left overnight at 18 °C. Cells were harvested and resuspended in 200 mL of wash buffer (50 mM Tris, 300 mM NaCl, 12.5 mM imidazole, 2 mM β-mercaptoethanol (BME), pH = 8.0). The proteins were isolated by homogenizing the cell pellets at a pressure of 40 psi using Emulsiflex-C3 homogenizer. The homogenized mixture was centrifuged at 40,000 x g for 30 min at 4 °C and the supernatant was loaded onto a nickel-nitrilotriacetic acid affinity column (Qiagen) pre-equilibrated with wash buffer. After washing the column with wash buffer containing 12.5 mM imidazole, the bound protein was eluted with 250 mM imidazole. Fractions containing protein were verified using SDS-PAGE (sodium dodecyl sulfate polyacrylamide gel electrophoresis). The protein containing fractions was dialyzed into dialysis buffer (50 mM Tris pH 8, 300 mM NaCl and 2 mM BME) and in a next step to ITC buffer (25 mM HEPES pH 7.5, 100 mM NaCl, 10 mM $MgCl_2$ and 0.5 mM TCEP). The protein was concentrated (measured using Nanodrop-1000), aliquotted and stored at -80 °C.

Purity and exact mass of the 14-3-3γ protein was determined using a High Resolution LC-MS system consisting of a Waters ACQUITY UPLC I-Class system coupled to a Xevo G2 Quadrupole Time of Flight (Q-ToF). The system was comprised of a Binary Solvent Manager and a Sample Manager with Fixed-Loop (SM-FL). The protein was separated (0.3 mL/min) by the column (Polaris C18A reverse phase column 2.0 x 100 mm, Agilent) using a 15% to 75% acetonitrile gradient in water (0.1% v/v formic acid) before analysis in positive mode in the mass spectrometer. Deconvolution was performed using the MaxENTI algorithm in the Masslynx v4.1 (SCN862) software.

Biotinylation of 14-3-3γ:

To 900 μL of a 0.35 mM solution of 14-3-3γ (10 mg, 0.32 μmol) in reaction buffer (25 mM HEPES, 100 mM NaCl, 10 mM $MgCl_2$, 0.5 mM TCEP, pH = 7.5) was added 100 μL of a 6.3 μM solution (in DMF) of NHS-PEG4-biotin (2 eq, 0.63 μmol). Reaction was allowed to proceed for 1 h at ambient temperature. At this time, reaction was quenched with the addition of 5 mL of quenching buffer (25 mM Tris, 100 mM NaCl, 10 mM $MgCl_2$, 0.5 mM TCEP, pH = 7.5). Sample was then spin washed 5x (10k MW cutoff, 15 mL spin filter), from quenching buffer back into reaction buffer, to remove excess biotin reagent. Protein concentration, measured by absorbance at 280 nm (ε = 37,945 $M^{-1}cm^{-1}$), was determined to be 85 μM. Protein was aliquotted and stored at -80 °C.

Preparation of a non-canonical $(X)_4(pS)(X)_4$-$CONH_2$ library:

Library design: $(X)_4(pS)(X)_4K$-$CONH_2$

SPPS:

2.9 g of 30 μm TentaGel resin (0.26 mmol/g, 0.74 mmol, 2.0 x 10^8 beads) was transferred to a 100 mL peptide synthesis vessel, swollen in DMF, and then washed with DMF (3x). Fmoc-Rink amide linker (2.0 g, 3.7 mmol, 5 eq) was dissolved in HATU solution (0.38 M in DMF, 8.8 mL, 3.3 mmol), activated with DIEA (1.9 mL, 11 mmol) immediately prior to coupling, and added to resin bed. Coupling was performed for 20 min; after this time, resin was washed with DMF (100 mL). Fmoc removal was carried out by treatment of resin with 20% piperidine in DMF (1 x 50 mL flow wash; 2 x 50 mL, 5 min batch treatments). Resin was then washed with DMF (150 mL). Coupling of Fmoc-Lys(Boc)-OH, subsequent Fmoc removal, and DMF washes were performed in the same manner.

At this stage, resin was suspended in DMF (50 mL), and divided evenly among 18 x 10 mL fritted plastic syringes using a 5 mL Eppendorf pipette. Couplings were performed as follows: Fmoc-protected amino acids (0.29 mmol) in HATU solution (0.38M, 683 μL, 0.26 mmol) were activated with DIEA (150 μL, 0.86 mmol). Each of the following amino acid derivatives was added to a single portion of resin (theory: ~190 mg resin, 40 μmol): Fmoc-D-Leu-OH, Fmoc-D-Lys(Boc)-OH, Fmoc-D-Asp(OtBu)-OH, Fmoc-D-Gln(Trt)-OH, Fmoc-β-Ala-OH, Fmoc-L-β-HomoSer(tBu)-OH*,* Fmoc-β-HomoThr(tBu)-OH, Fmoc-Ala(β-cyclopropyl)-OH, Fmoc-L-Cha-OH, Fmoc-Nva-OH, Fmoc-L-Aad(OtBu)-OH, Fmoc-Dab(Boc)-OH, Fmoc-L-Orn(Boc)-OH, Fmoc-Hyp(tBu)-OH, Fmoc-L-Ala*(*4-thiazoyl)-OH, Fmoc-L-Phe(4-NHBoc)-OH, Fmoc-Phe(4-F)-OH, and Fmoc-Phe(4-NO_2)-OH. Couplings were performed for 20 min. Following four cycles of split-and-pool synthesis, Fmoc-Ser(PO(OBzl)OH)-OH (1.9 g, 3.7 mmol, 5 eq) was dissolved in HATU solution (0.38 M in DMF, 8.8 mL, 3.3 mmol), activated with DIEA (1.9 mL, 11 mmol) immediately prior to coupling, and added to resin bed. Coupling was performed for 2.5 h; after this time, resin was washed with DMF (100 mL). Fmoc removal was carried out by treatment of resin with 20% piperidine in DMF (1 x 50 mL flow wash; 2 x 50 mL, 5 min batch treatments). Resin was then washed with DMF (150 mL). Four more cycles of split-and-pool synthesis were then performed as described above.

Following removal of N-terminal Fmoc group, resin was washed with DMF (150 mL) and transferred to fritted plastic syringes (20 mL). Resin was then washed with DCM (3x) and dried under reduced pressure. 1.0 mg of dried resin (theory: 4.8 x 10^4 beads) was weighed into a plastic tube and set aside for later characterization (**Characterization of a non-canonical $(X)_4(pS)(X)_4$-$CONH_2$ library**).

Cleavage from resin and solid phase extraction:

Libraries were globally deprotected and cleaved from resin as described in **Preparation of 2 x 10^6, 2 x 10^7, and 2 x 10^8-member $(X)_9K$-$CONH_2$ libraries**. Crude, lyophilized powders were resuspended in 95/5 water/acetonitrile (0.1% TFA), and

purified over Supelclean™ LC-18 SPE cartridges (2 g bed mass, 45 µm particle size, 12 mL; Millipore Sigma, P/N 57117). Procedure is described in **Preparation of 2 x 10^6, 2 x 10^7, and 2 x 10^8-member $(X)_9K\text{-}CONH_2$ libraries**.

Preparation of stock solution:

Lyophilized powder of library (123 mg) was dissolved in DMF (1.16 mL) and diluted with 1x PBS (10.5 mL) to a final library concentration of 8 mM (~40 pM/member) and final DMF concentration of 10% (v/v). Stock solutions were aliquotted out and stored at -80 °C. Aliquots were thawed on ice prior to use.

Characterization of a non-canonical $(X)_4(pS)(X)_4$-$CONH_2$ library:

Sample preparation:

A 1.0 mg aliquot of library resin (from **Preparation of a non-canonical $(X)_4(pS)(X)_4$-$CONH_2$ library**) was suspended in 1.0 mL of Milli-Q water and sonicated to achieve a homogenous suspension (theory: 4.8 x 10^4 beads/mL). A 10 μL aliquot (theory: 475 beads; 1 pmol/peptide) was transferred to a plastic tube, spun down, and supernatant removed. Beads were then subjected to treatment with 94% (v/v) TFA, 2.5% (v/v) ethanedithiol, 2.5% (v/v) water, and 1.0% (v/v) triisopropylsilane, for 10 min in a 60 °C water bath. TFA was then evaporated under a stream of nitrogen, and cleaved peptide was resuspended in Milli-Q water (0.1% TFA). Sample was purified over a C18 ZipTip® (0.6 μL, MilliporeSigma, P/N ZTC18S096), eluted in 30/70 water/acetonitrile (0.1% TFA), and lyophilized. Powder was resuspended in 90 μL of Milli-Q water (0.1% FA), and 1 μL (~40 fmol/peptide) was submitted for nLC-MS/MS analysis.

NanoLC-MS/MS analysis and *de novo* peptide sequencing:

Analysis and *de novo* sequencing was performed as described in **Characterization of 2 x 10^6, 2 x 10^7, and 2 x 10^8-member $(X)_9K$-$CONH_2$ libraries**. Non-canonical amino acids with masses that differ from natural amino acids were sequenced as fixed modifications on residues that had been excluded from the monomer set. Specifically, β-homothreonine was identified as fixed modification on Asn (+1.0204), aminoadipic acid as a fixed modification on Glu (+14.0156), diaminobutyric acid as a fixed modification on Gly (+43.0421), ornithine as a fixed modification on Cys (+11.0701), hydroxyproline as a fixed modification on Pro (+15.9948), cyclopropyl alanine as a fixed modification on Met (-19.9721), cyclohexyl alanine as a fixed modification on Phe (+6.0469), 4-amino phenylalanine as a fixed modification on Arg (+5.9782), 4-fluoro phenylalanine as a fixed modification on Tyr (+1.9957), 4-nitro phenylalanine as a fixed modification on Trp (+5.9742), thiazolyl alanine as a fixed modification on His (+16.9611), and phosphoserine as a fixed modification on Ser (+79.9663). β-alanine, β-homoserine, and norvaline were identified as Ala, Thr, and Val, respectively. D-Leu, D-Lys, D-Asp, and D-Gln were identified as Leu, Lys, Asp, and Gln, respectively.

Affinity selections against 14-3-3γ:

Preparation of 14-3-3γ-functionalized and 12ca5-functionalized magnetic beads:

MyOne Streptavidin T1 Dynabeads (2 x 300 μL of 10 mg/mL stock) were transferred to 1.7 mL plastic centrifuge tubes, and placed in a magnetic separation rack. Beads were washed 3 x 1 mL w/ blocking buffer (10% FBS, 100 μM tri-tryptophan additive, 1x PBS), and then treated with 9 μL of biotinylated 14-3-3γ (85 μM; 0.79 nmol; diluted to 300 μL with blocking buffer) or 300 μL of biotinylated 12ca5 (1.5 μM; 0.45 nmol). The resulting suspensions were transferred to a rotating vertical mixer and allowed to incubate for 1 h at 4°C. After this time, the beads were returned to the separating rack, the supernatant was removed, and the beads were washed 3 x 1 mL w/ blocking buffer. Beads were then resuspended in 300 μL of blocking buffer.

Affinity capture:

Library (10 fmol/member) was incubated with 100 μL (1 mg) portions of protein-immobilized magnetic beads (prepared above) in the presence of blocking buffer (final volume: 1 mL) on a rotating mixer for 1 h at 4 °C. Final conditions: 1 mg/mL magnetic beads, 10 pM/member library.

Elution and nanoLC-MS/MS:

Bound peptides were eluted as described in **Affinity selections of 2 x 10^6, 2 x 10^7, and 2 x 10^8-member libraries against 12ca5**. NanoLC-MS/MS analysis was performed as described in **Characterization of 2 x 10^6, 2 x 10^7, and 2 x 10^8-member $(X)_9K$-$CONH_2$ libraries** and **Affinity selections of 2 x 10^6, 2 x 10^7, and 2 x 10^8-member libraries against 12ca5**.

SPPS of FITC-labeled putative 14-3-3γ-binding peptides:

Preparation of Lys(boc)–β-Ala–Lys(alloc)-Rink amide peptidyl resin:

Rink amide ChemMatrix resin (1.0 g, 0.45 mmol/g) was transferred to a 20 mL plastic fritted syringe, washed 3 x 20 mL with DMF, and swollen in 20 mL of DMF for 1 h. Fmoc-L-Lys(alloc)-OH (2.25 mmol, 5 eq, 1.0 g) was weighed into a glass vial and dissolved in 0.38 M HATU in DMF (5.34 mL, 2.0 mmol, 0.9 eq HATU). To this solution was added DIEA (1.13 mL, 6.5 mmol, 2.9 eq), and activated amino acid solution was added to the resin bed. Coupling was allowed to proceed for 1 h. At this time, the reaction mixture was drained and the resin was washed 3 x 20 mL with DMF. Fmoc removal was carried out by treatment of the resin with 20% piperidine in DMF (2 x 20 mL, 5 min batch treatments). Resin was then washed 3 x 20 mL with DMF. Couplings of Fmoc-β-Ala-OH and Fmoc-Lys(boc)-OH were performed in the same manner. After removal of the N-terminal Fmoc group, the resin was suspended in DMF (~20 mL) and split out 10 ways into 6 mL plastic fritted syringes. (Note: β-Ala was incorporated as a spacer between the sequences obtained from selection, which all bear a C-terminal lysine, and Lys(alloc), to which a FITC fluorophore will be coupled for fluorescence anisotropy studies.)

Main chain elaboration of select sequences derived from affinity selection:

One portion of peptidyl resin prepared above was used for every construct prepared (four in total). Couplings were performed as follows: Fmoc-protected amino acids (0.23 mmol) in HATU solution (0.38M, 534 μL, 0.2 mmol) were activated with DIEA (113 μL, 0.65 mmol) and added to the resin bed. Couplings were allowed to proceed for 20 min. At this time, reaction mixtures were drained and resins were washed 3 x 5 mL with DMF. Fmoc removal was carried out by treatment of the resin with 20% piperidine in DMF (1 x 5 mL flow wash; 2 x 5 mL, 5 min batch treatments). Following removal of N-terminal Fmoc group, resins were washed 3 x 5 mL with DMF, then 3 x 5 mL with DCM.

Incorporation of FITC:

The free amine on the N-terminus was Boc-protected as follows: to a solution of di-tert-butyl dicarbonate (0.45 mmol, 10 eq, 400 mM) in DCM was added DIEA (10 eq), and solution was added to each portion of resin. Coupling was allowed to proceed for 1 h. At this time, resin was washed 3 x 5 mL with DCM and coupling was repeated as described. Resin was washed 5 x 5 mL with DCM.

Alloc removal was achieved as follows: each portion of resin was treated with a solution of tetrakis(triphenylphosphine)palladium(0) (0.5 eq, 20 mM) and phenylsilane (20 eq) in DCM, 2 x 45 min. Resins were then washed 3 x 5 mL with DCM, then 3 x 5 mL with DMF.

FITC was installed on the free amine on each C-terminal lysine by treating each portion of resin with fluorescein isothiocyanate isomer I (10 eq, 400 mM in 4:1 DMF:DCM) and DIEA (15 eq) for 1.5 h. Reactions were kept under aluminum foil for

the duration of the coupling. Reaction mixtures were then drained, and resins were washed 3 x 5 mL with DMF, 3 x 5 mL with DCM, and dried under reduced pressure.

Cleavage from resin, HPLC purification, and LC-MS characterization:

Detailed procedures can be found in **Solid phase peptide synthesis (SPPS) of anti-hemagglutinin (HA) epitope and analogues**. Sequences, structures, and LC-MS traces are shown below.

Fluorescence anisotropy binding assay of 14-3-3γ-binding peptides:

All fluorescence anisotropy affinity measurements were conducted in FA buffer (10 mM HEPES pH 7.4, 150 mM NaCl, 0.1% (v/v) Tween 20, 0.1% (w/v) BSA). During each assay a 1:1 dilution series (starting at 100 μM) of 14-3-3γ was made in wells containing a fixed concentration FITC-labeled peptide (10 nM or 50 nM). This was done in polystyrene non-binding low-volume Corning Black Round Bottom 384-well plates (Corning 4514). Measurements were performed at ambient temperature using a Tecan Infinite F500 plate reader with the following parameters: λ_{ex}: 485 (20) nm; λ_{em}: 535 (25) nm; mirror: Dichroic 510; flashes: 20; integration time: 50 μs; settle time; 0 μs; gain: manual 90; Z-position: calculated from well. The G-factor was set at 35 mP based on wells containing only the FITC-labeled peptide.

SPPS of unlabeled 14-3-3γ-binding peptides:

SPPS was carried out on Rink amide ChemMatrix resin (0.45 mmol/g). Couplings for main chain elaboration were carried out as described in **SPPS of FITC-labeled putative 14-3-3γ-binding peptides**. Cleavage from resin, HPLC purification, and LC-MS characterization were performed as described in **Solid phase peptide synthesis (SPPS) of anti-hemagglutinin (HA) epitope and analogues**. Sequences, structures, and LC-MS traces are shown below.

Competition fluorescence anisotropy binding assay of 14-3-3γ-binding peptides:

All fluorescence anisotropy affinity measurements were conducted in FA buffer (10 mM HEPES pH 7.4, 150 mM NaCl, 0.1% (v/v) Tween-20, 0.1% (w/v) BSA). During each assay a 1:1 dilution series (starting at 10 μM) of unlabeled peptides were made in wells containing a fixed concentration FITC-labeled biExoS (10 nM; FITC-O1Pen-QGLLDALDLAS(GGSGGGGSGG)QGLLDALDLAS-$CONH_2$)[5], and 14-3-3γ (20 nM). This is done in polystyrene non-binding low-volume Corning Black Round Bottom 384-well plates (Corning 4514). Measurements were performed at ambient temperature using a Tecan Infinite F500 plate reader with the following parameters: λ_{ex} = 485 (20) nm; λ_{em} = 535 (25) nm, mirror: Dichroic = 510, flashes: 20; integration time: 50 μs; settle time: 0 μs; gain: manual 90; and Z-position: calculated from well. The G-factor was set at 35 mP based on wells containing only the FITC-labeled peptide.

Expression of 14-3-3σΔc:

The 14-3-3σ isoform with a truncated C-terminus after T321 (ΔC, to enhance crystallization) was expressed by transforming pROEX HTb plasmid, containing a His6-tagged 14-3-3σΔc gene and ampicillin resistance gene, to BL21(DE3) Escherichia coli cells (Novagen). Cells were grown at 37 °C, with 0.1 mg/mL ampicillin, and protein expression was induced using 0.4 μM IPTG and 1 mM $MgCl_2$ and left overnight at 18 °C. Cells were harvested and resuspended in 200 mL of wash buffer (50 mM Tris, 300 mM NaCl, 12.5 mM imidazole, 2 mM β-mercaptoethanol (BME), pH = 8.0). The proteins were isolated by homogenizing the cell pellets at a pressure of 40 psi using Emulsiflex-C3 homogenizer. The homogenized mixture was centrifuged at 40,000 x g for 30 min at 4 °C and the supernatant was loaded onto a nickel-nitrilotriacetic acid affinity column (Qiagen) pre-equilibrated with wash buffer. After washing the column with wash buffer containing 12.5 mM imidazole, the bound protein was eluted with 250 mM imidazole. Fractions containing protein were verified using SDS-PAGE (sodium dodecyl sulfate polyacrylamide gel electrophoresis). The protein containing fractions were dialyzed overnight in dialysis buffer (50 mM Tris pH 8, 300 mM NaCl and 2 mM BME) containing TEV protease for His-tag cleavage at 4°C. Non-cleaved His-tagged 14-3-3σΔc was than captured using a nickel-nitrilotriacetic acid affinity column (Qiagen) pre-equilibrated with wash buffer after which the flow through was dialyzed into ITC buffer (25 mM HEPES pH 7.5, 100 mM NaCl, 10 mM MgCl2 and 0.5 mM TCEP) at 4°C. The protein was concentrated (measured using Nanodrop-1000), aliquotted and stored at -80 °C. Purity and exact mass of the 14-3-3σΔc protein was determined as described in **Expression of 14-3-3γ**.

Binding validation of 14-3-3.12 and 14-3-3σΔc:

Fluorescence anisotropy measurements were carried according to the general protocol described in **Fluorescence anisotropy binding assay of 14-3-3γ-binding peptides**.

Single crystal X-Ray diffraction analysis of 14-3-3σΔc in complex with synthetic peptide binder 14-3-3.12:

Unlabeled **14-3-3.12** was soaked into preformed crystals of 14-3-3σΔc, which grew in 25% PEG400, 5% Glycerol, 0.2 M CaCl2, 0.1 M HEPES pH 7.5 plus 2 mM BME within two weeks. The soaked crystal was fished after 15 days of incubation and flash-frozen in liquid nitrogen. Diffraction data was collected at 100K on an in-house Rigaku Micromax-003 (Rigaku Europe, Kemsing Sevenoaks, UK) sealed tube X-ray source and Dectris Pilatus 200K detector (DECTRIS Ltd Baden-Daettwil, Switzerland).

Integration, scaling and merging of data was done using DIALS (CCP4i2) after which molecular replacement is done with MOLREP (CCP4i2) using PDB 4JC3 as search model. A three-dimensional structure of **14-3-3.12** was generated using eLBOW (Phenix)[6] after which it was built within this structure based on visual inspection of Fo-Fc and 2FoFc electron density maps in Coot[7]. Several rounds of model building and refinement (based on isotropic b-factors and standard set of stereo-chemical restraints: covalent bonds, angles, dihedrals, planarities, chiralities, non-bonded) were performed using Coot and Phenix.refine[8,9]. See **Supplementary Table 12** for data collection and refinement statistics.

Supplementary Figures

Amino acid sequence: YPYDVPDYA$^{\alpha}$-*CONH₂*

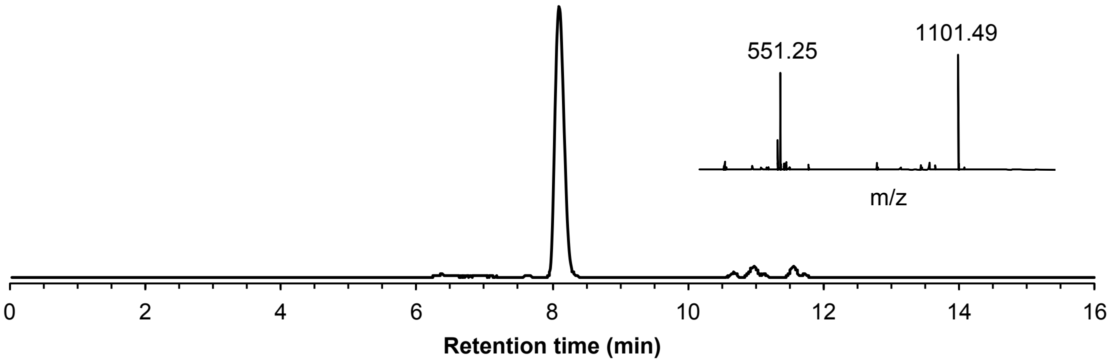

Supplementary Figure 1. LC-MS characterization of the native HA epitope (monoisotopic mass: 1100.47 Da; found: 1100.48 Da).

Amino acid sequence: FDYEDYAEWK$^{\alpha}$-*CONH₂*

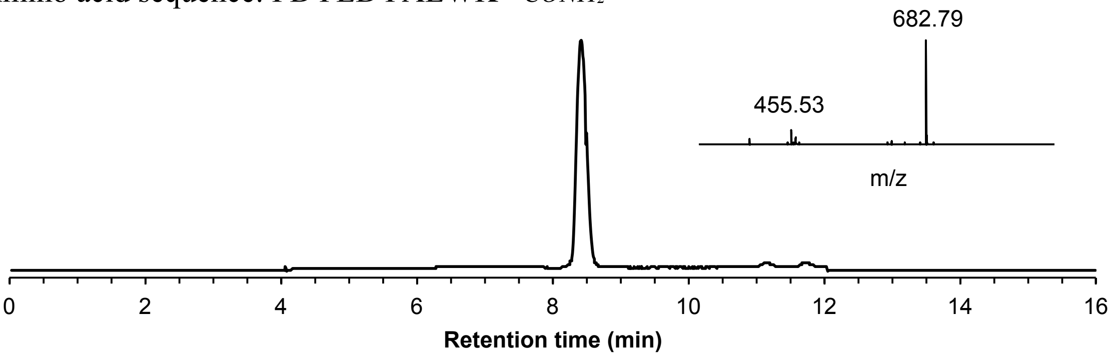

Supplementary Figure 2. LC-MS characterization of a 12ca5-binding peptide identified from early affinity selection experiments (monoisotopic mass: 1363.56 Da; found: 1363.57 Da).

Amino acid sequence: YPYDVPDYG$^{\alpha}$-*CONH₂*

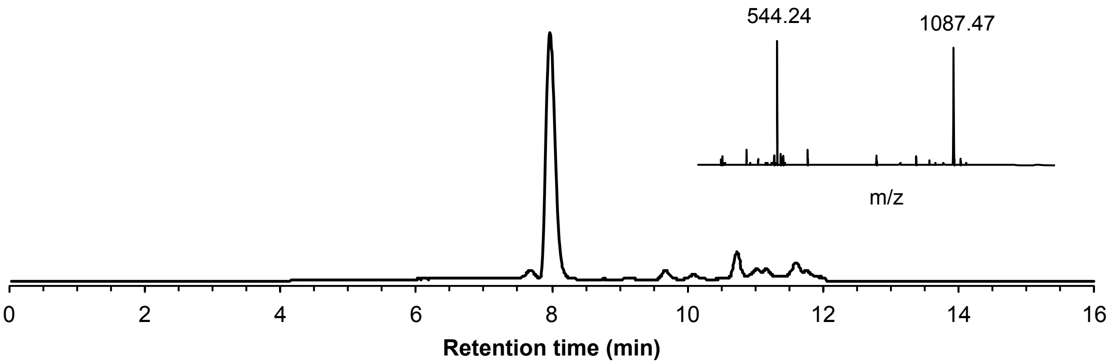

Supplementary Figure 3. LC-MS characterization of HA epitope mutant Ala9Gly (monoisotopic mass: 1086.46 Da; found: 1087.47 Da).

Amino acid sequence: Gypyeydwe$^{\alpha}$-*CONH₂*

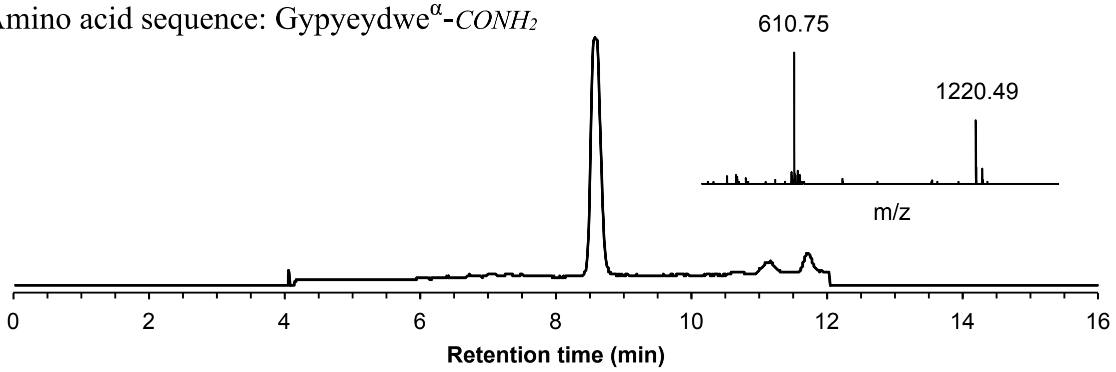

Supplementary Figure 4. LC-MS characterization of a 12ca5-binding peptide, comprised of D-amino acids, derived from the loop of a 12ca5-binding xenoprotein (monoisotopic mass: 1219.47 Da; found: 1219.48 Da).

Amino acid sequence: ypyefdeph$^{\alpha}$-*CONH₂*

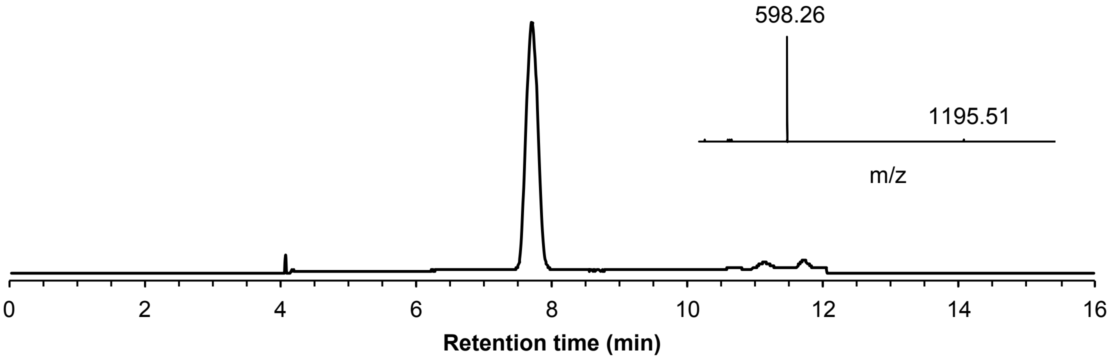

Supplementary Figure 5. LC-MS characterization of a 12ca5-binding peptide, comprised of D-amino acids, derived from the loop of a 12ca5-binding xenoprotein (monoisotopic mass: 1194.49 Da; found: 1194.50 Da).

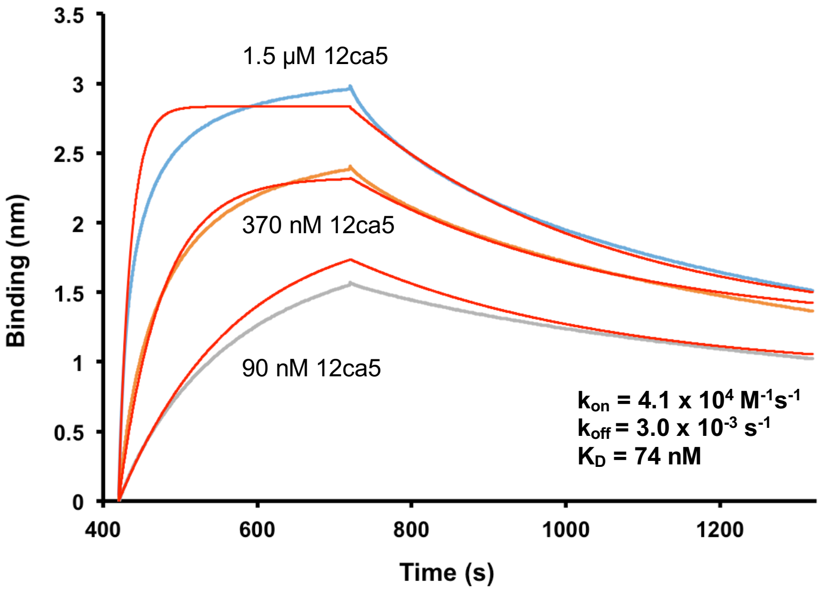

Supplementary Figure 6. 12ca5 ligand 'FDYEDYAEWKK(biotin)' binds to 12ca5 with a k_{on} of 4.1 x 10^4 $M^{-1}s^{-1}$ and a k_{off} of 3.0 x 10^{-3} s^{-1}, as measured by BioLayer Interferometry.

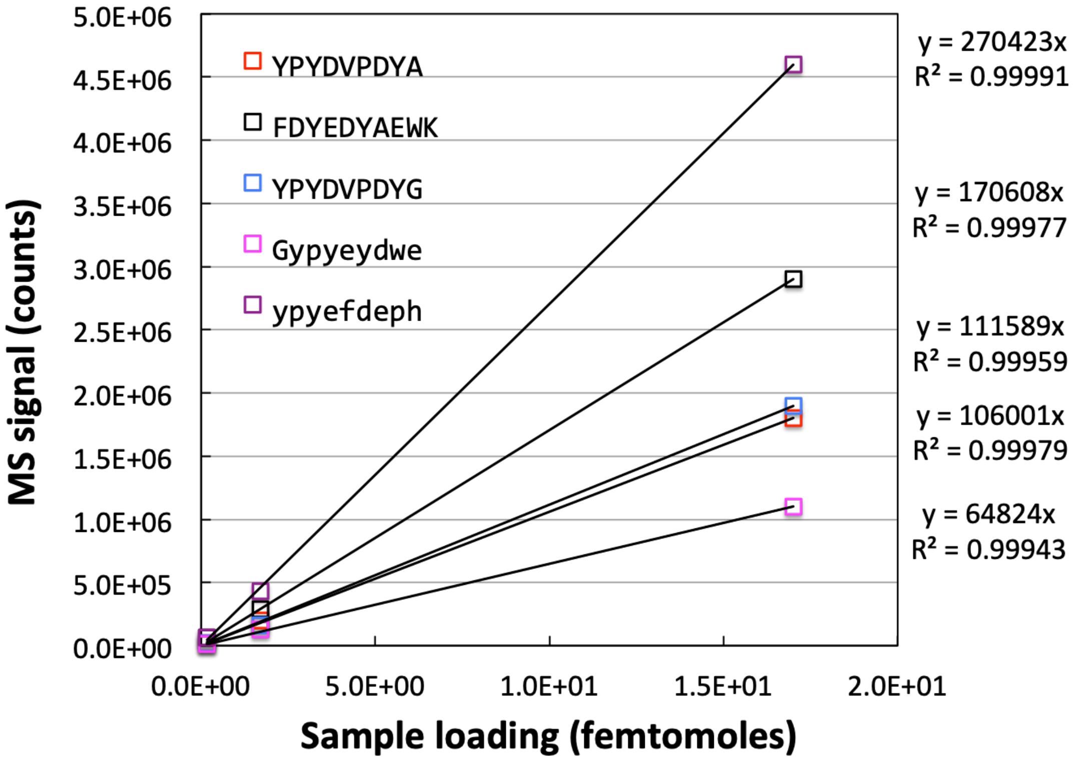

Supplementary Figure 7. A plot of MS signal as a function of sample loading illustrates the ~5-fold variation in signal magnitude between 5 different anti-HA binders. For each peptide, a 10-fold change in sample loading corresponded to a 10-fold change in signal. Therefore, for a given peptide, the ratio of signals in two samples corresponds to their relative concentrations.

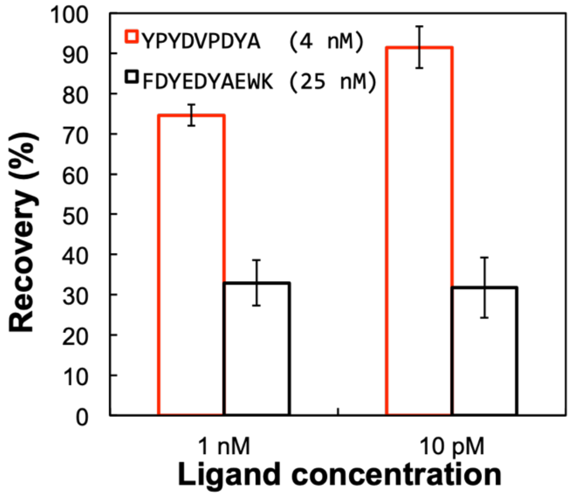

Supplementary Figure 8. Recoveries of 12ca5-binding peptides obtained by affinity selection from solutions containing either 1 nM or 10 pM/peptide of starting mixture. Of the 5 peptides examined (**Supplementary Table 1**), only the 2 highest-affinity binders were significantly retained. Error bars correspond to the standard deviations in recovery obtained by 3 technical replicates.

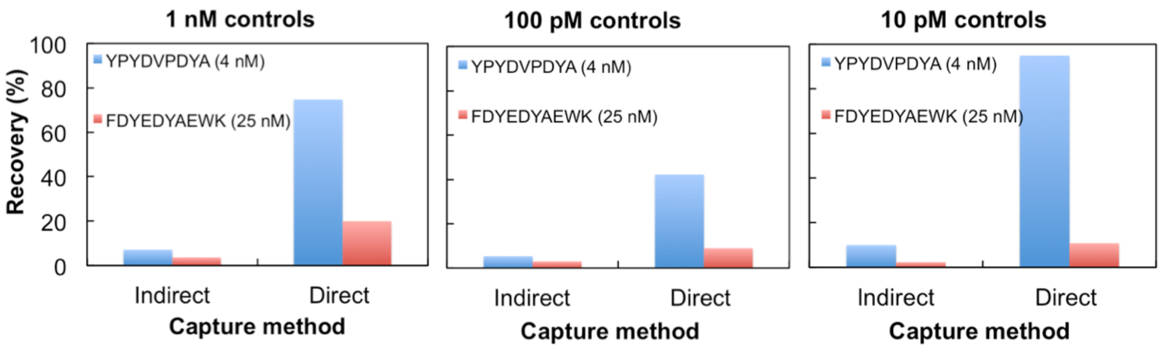

Supplementary Figure 9. High-affinity 12ca5-binding peptides are efficiently recovered with 'direct' capture at high dilution. Pulldowns were performed using 100 nM 12ca5 and the indicated ligand concentrations. Recoveries were determined by nLC-MS analysis of the peptide mixtures obtained by 'direct' or 'indirect' capture, relative to a reference analysis (the amount of material corresponding to 100% retention). Raw ion counts were normalized to an internal standard to account for run-to-run variability in MS response.

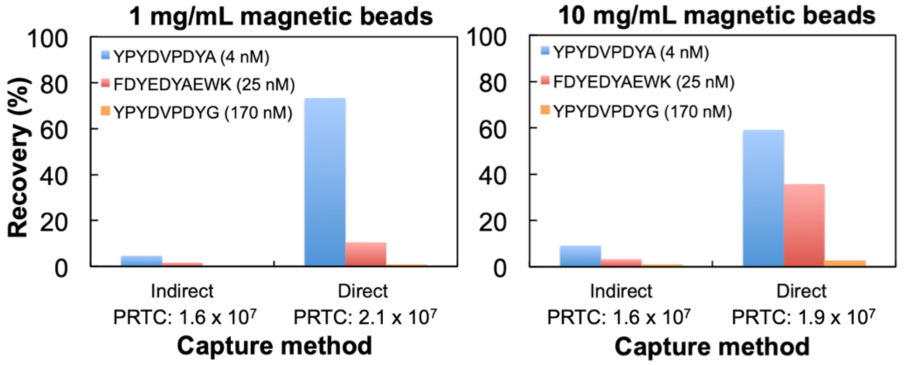

Supplementary Figure 10. Magnetic bead concentration does not significantly improve recovery for high affinity binders. For lower affinity binders, recovery is only slightly improved. Counts of reference PRTC ion (m/z = 422.74) are indicated below each condition.

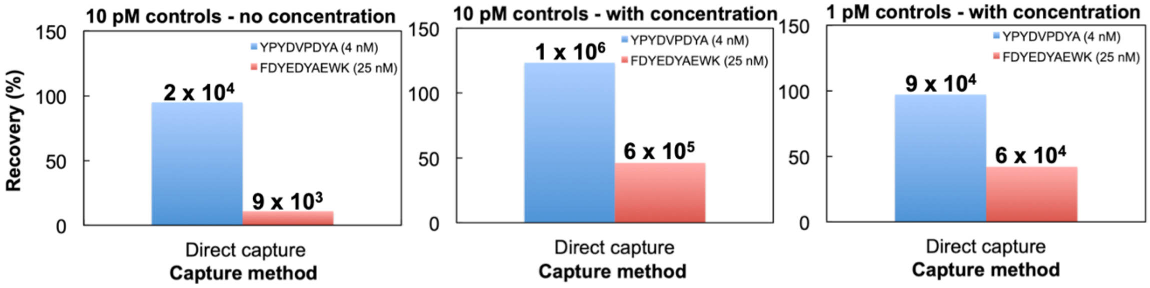

Supplementary Figure 11. Detection of recovered 12ca5 binders from 1 pM concentration is enabled with post-pulldown concentration of eluate. Efficient recovery and robust MS signals were obtained from as little as 1 pM of 12CA5 binders in 1 mL volume.

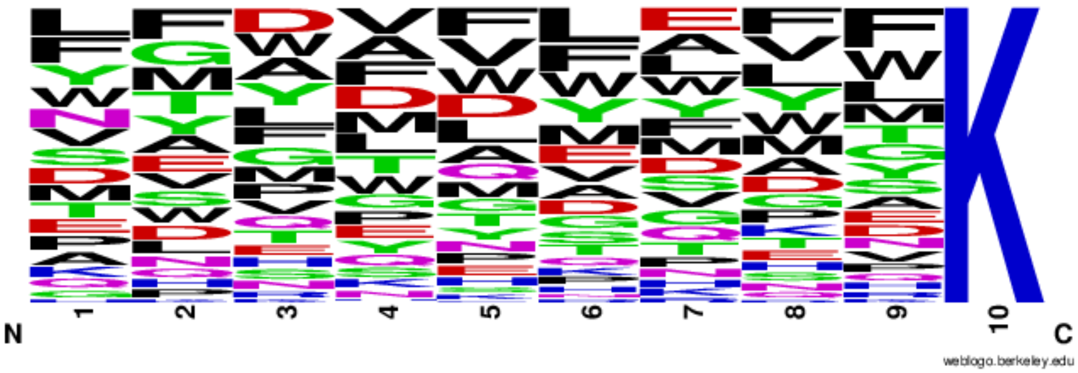

Supplementary Figure 12. nLC-MS/MS characterization of a $(X)_9K$-$CONH_2$ library, synthesized on 2 x 10^8 beads of 30 μM TentaGel resin, identifies 406 individual peptide sequences with an average local confidence (ALC) score ≥ 80 from a theory of 940 beads cleaved. A positional frequency plot based on these 406 sequences is shown.

Sequence: APYDVPDYA$^{\alpha}$-*CONH₂*

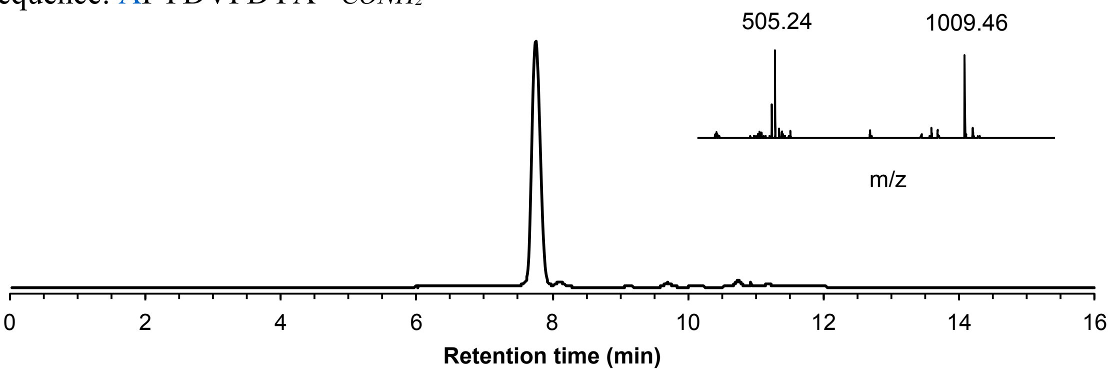

Supplementary Figure 13. LC-MS characterization of HA epitope Tyr1Ala mutant (monoisotopic mass: 1008.44 Da; found: 1008.46 Da).

Sequence: YAYDVPDYA$^{\alpha}$-*CONH₂*

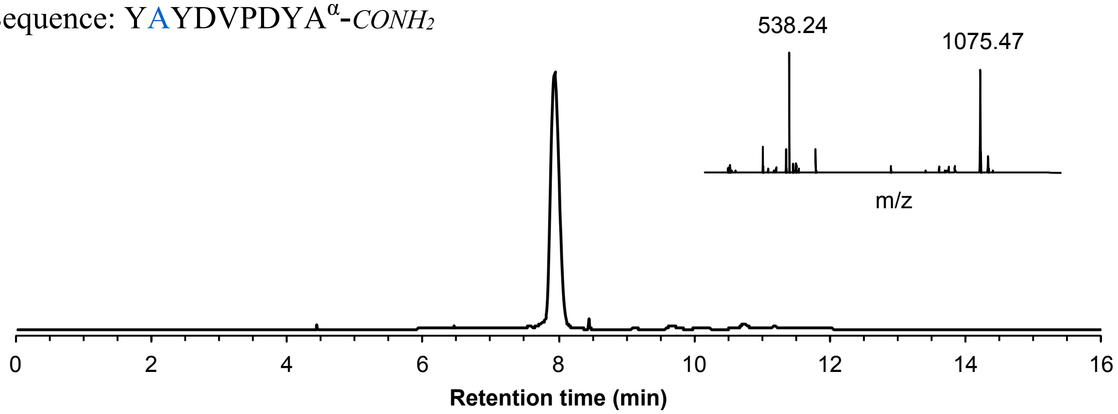

Supplementary Figure 14. LC-MS characterization of HA epitope Pro2Ala mutant (monoisotopic mass: 1074.46 Da; found: 1075.47 Da).

Sequence: YPADVPDYA$^{\alpha}$-*CONH₂*

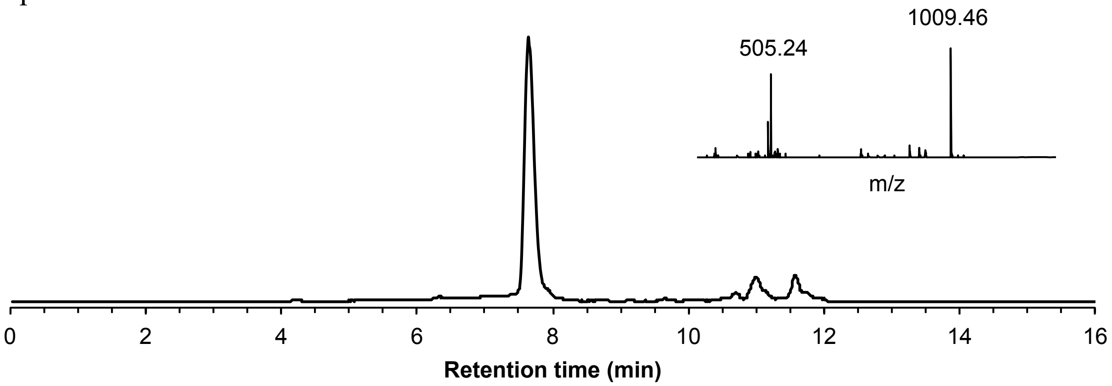

Supplementary Figure 15. LC-MS characterization of HA epitope Tyr3Ala mutant (monoisotopic mass: 1008.44 Da; found: 1008.46 Da).

Sequence: YPYAVPDYA$^{\alpha}$-*CONH₂*

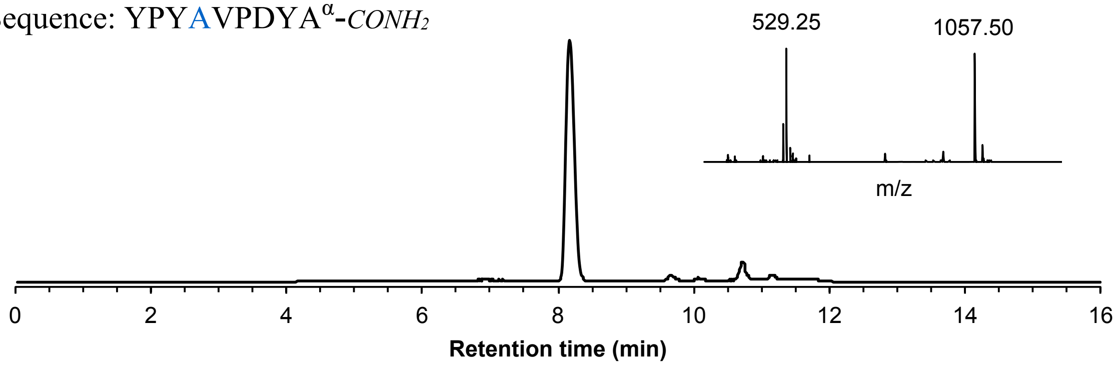

Supplementary Figure 16. LC-MS characterization of HA epitope Asp4Ala mutant (monoisotopic mass: 1056.48 Da; found: 1056.49 Da).

Sequence: YPYDAPDYA$^{\alpha}$-*CONH₂*

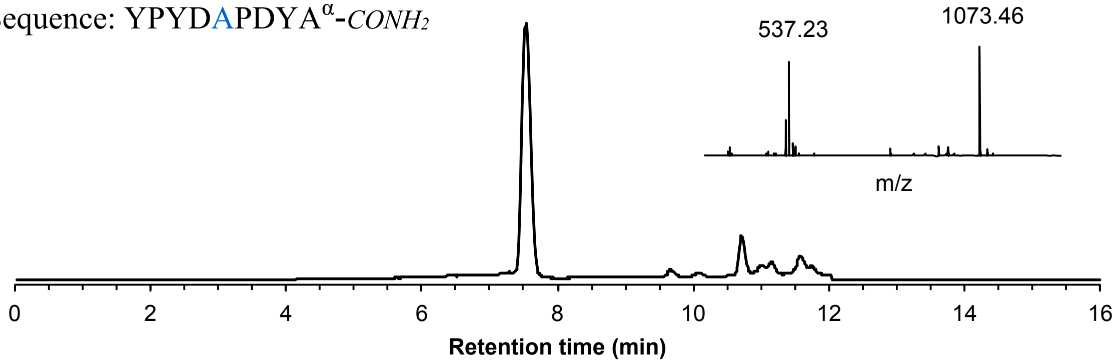

Supplementary Figure 17. LC-MS characterization of HA epitope Val5Ala mutant (monoisotopic mass: 1072.44 Da; found: 1072.45 Da).

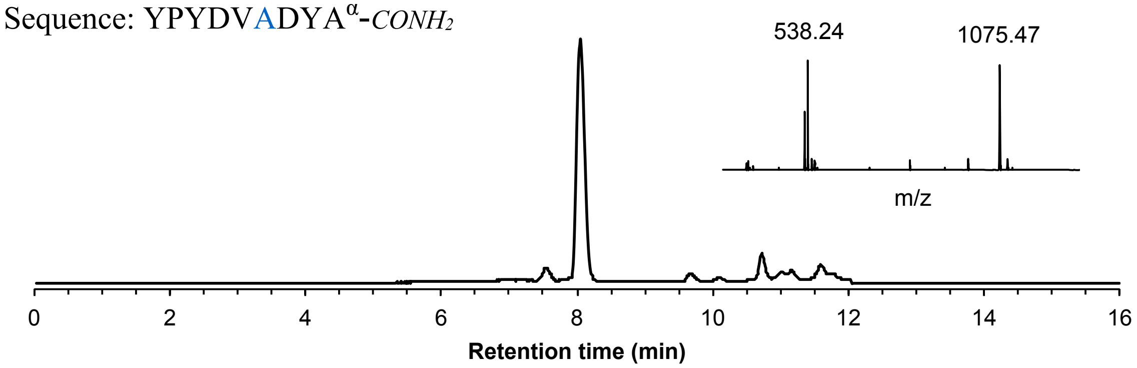

Supplementary Figure 18. LC-MS characterization of HA epitope Pro6Ala mutant (monoisotopic mass: 1074.46 Da; found: 1074.47 Da).

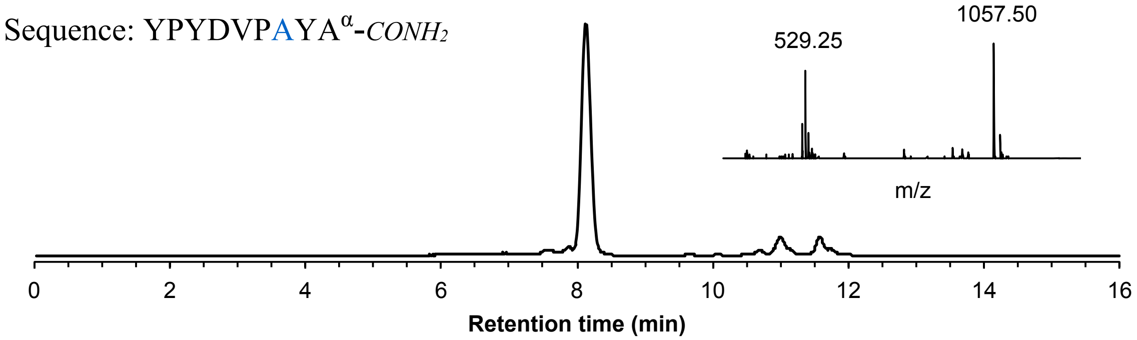

Supplementary Figure 19. LC-MS characterization of HA epitope Asp7Ala mutant (monoisotopic mass: 1056.48 Da; found: 1056.49 Da).

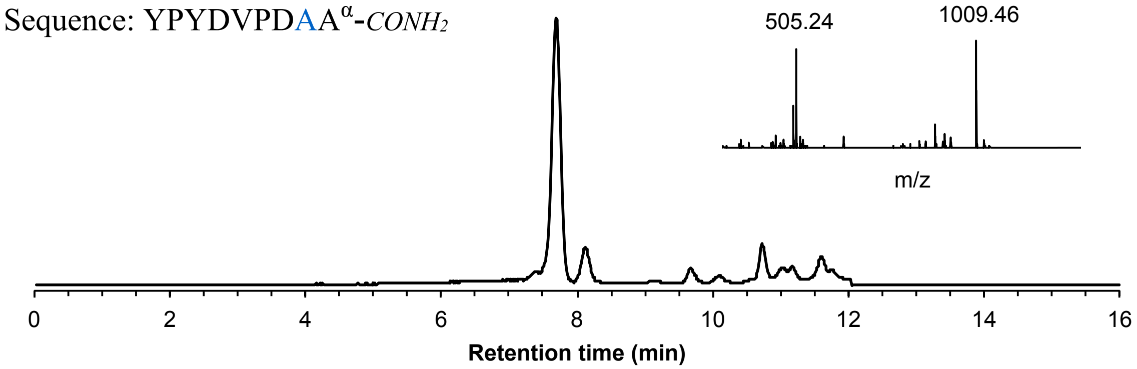

Supplementary Figure 20. LC-MS characterization of HA epitope Tyr8Ala mutant (monoisotopic mass: 1008.44 Da; found: 1008.46 Da).

LC-MS characterization of HA epitope mutant Ala9Gly is provided in **Supplementary Figure 3.**

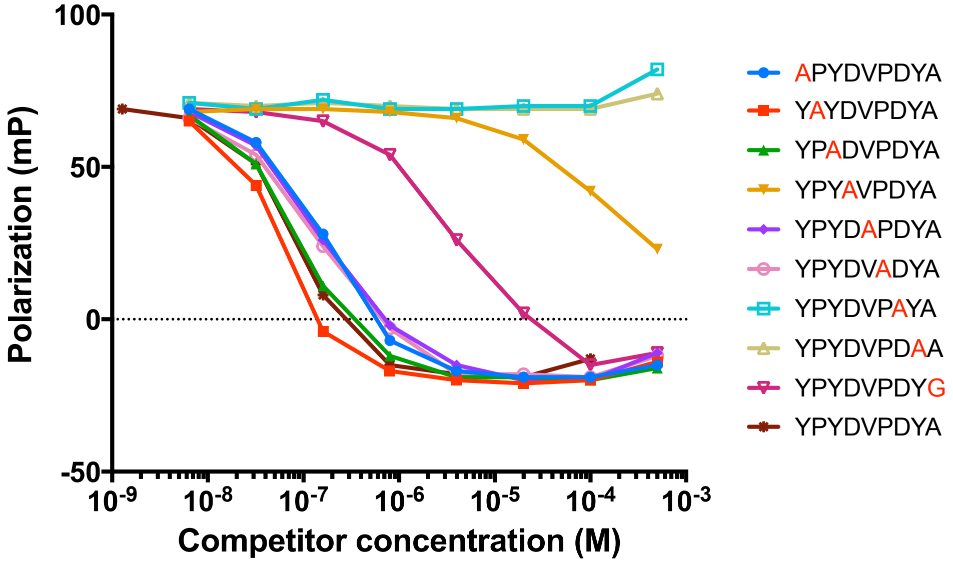

Supplementary Figure 21. Competition fluorescence polarization of HA epitope Ala scan mutants reveals Asp4, Asp7, Tyr8, and Ala9 as 'hot spot' residues. Mutations to Asp7 and Tyr8 completed abrogated affinity towards 12ca5, while mutations to Asp4 and Ala9 were deleterious but did not completely abolish binding. Mutations elsewhere had a lesser effect on affinity.

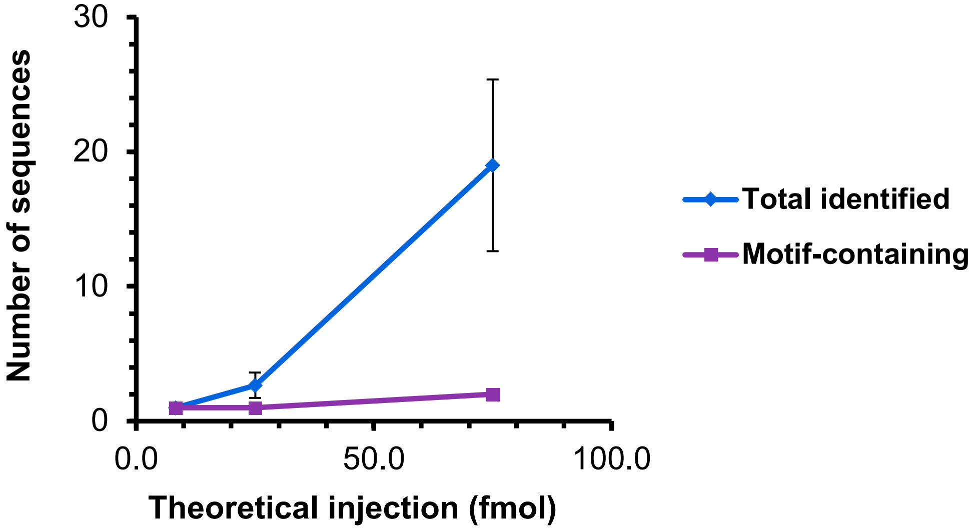

Supplementary Figure 22. Enrichment decreases at increased sample loadings in a selection for 12ca5-binding performed at 200 pM/member. At the highest injection amount analyzed, two additional DxxDY(A/S)-containing sequences were identified, along with an average of 18 additional background sequences. Error bars correspond to one standard deviation of three replicate experiments.

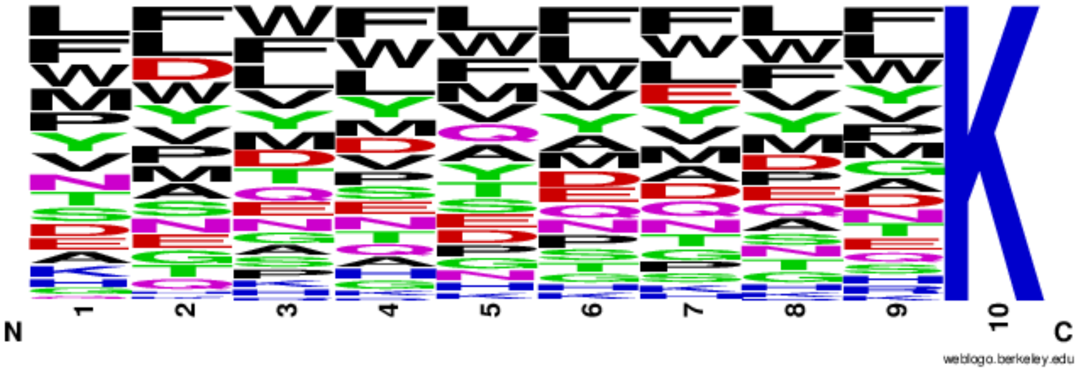

Supplementary Figure 23. nLC-MS/MS characterization of a $(X)_9K$-$CONH_2$ library, synthesized on 1.3 x 10^9 beads of 20 μM TentaGel resin, identifies 1471 individual peptide sequences with an ALC score ≥ 80 from a theory of 805 beads cleaved. A positional frequency plot based on these 1470 sequences is shown.

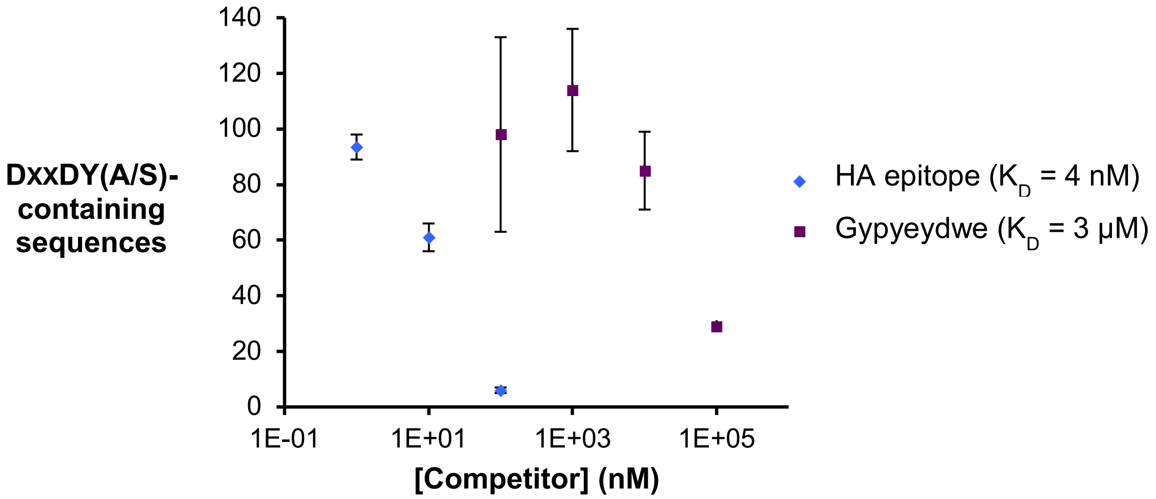

Supplementary Figure 24. Exogenous HA epitope (K_D = 4 nM) could impede recovery of DXXDY(A/S)-containing sequences when included at 1:1 stoichiometry relative to 12ca5, while the peptide 'Gypyeydwe' (K_D = 3 µM) inhibited recovery only when present at 1000:1 stoichiometry relative to 12ca5. Error bars correspond to standard deviations from two experimental replicates.

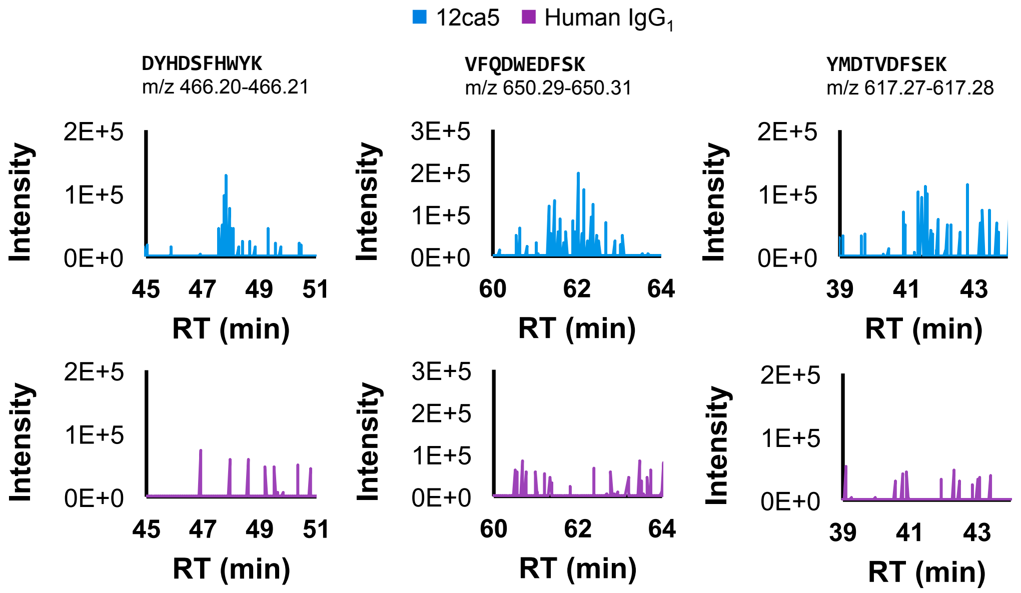

Supplementary Figure 25. Extracted ion chromatograms (EICs) for peptides that were sequenced in 12ca5 selections, but not IgG_1 selections, and which may be 12ca5 binders but were possibly mis-sequenced. All peptides shown contain either DXXDFS or DXXDSF. Because the dipeptide masses of ‘FS’ and ‘YA’ are identical, incomplete fragmentation could have led to erroneous sequence assignments in these cases. The EICs indicate that these peptides were enriched in 12ca5 selections, suggesting that they could in fact be 12ca5-binding peptides.

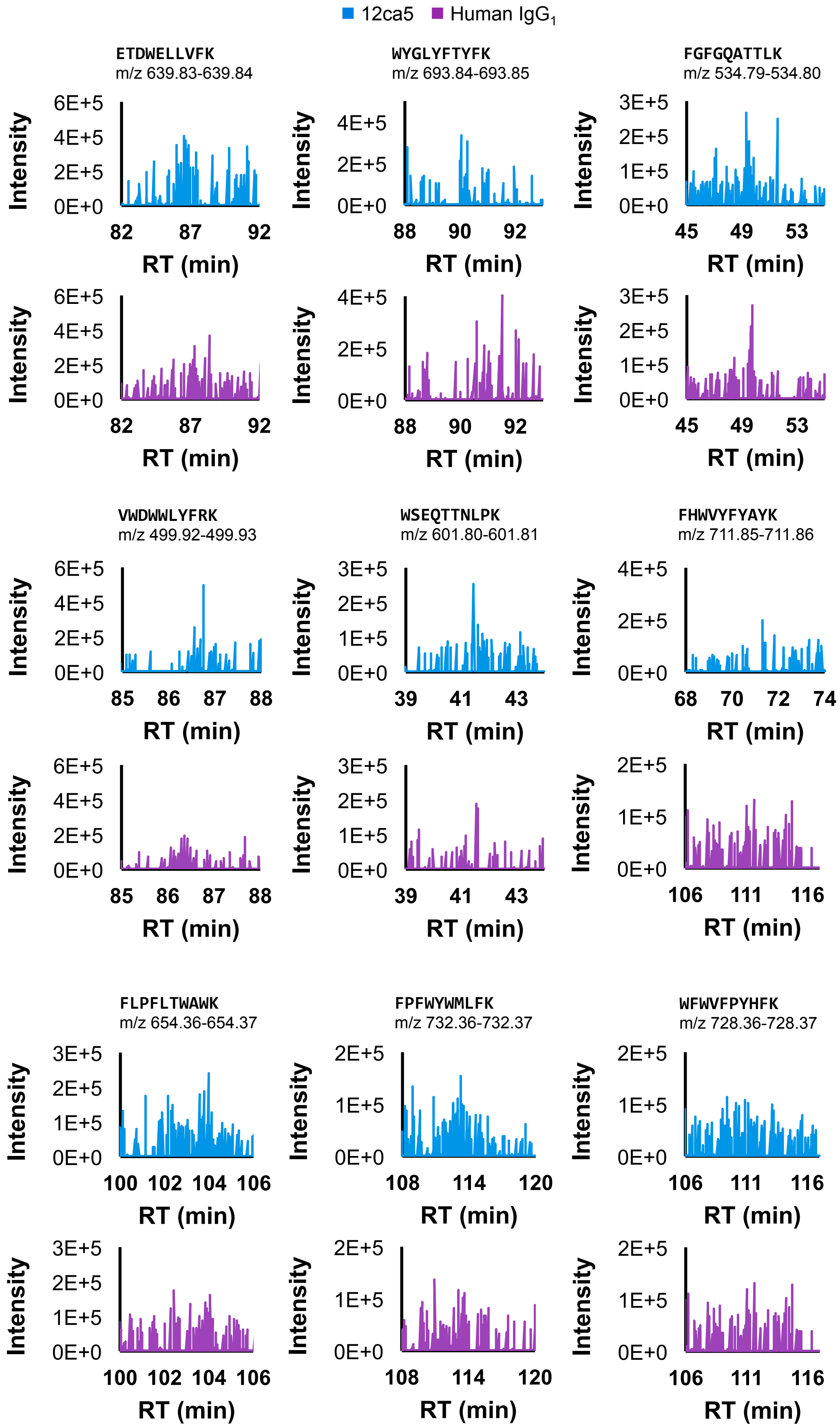
12ca5
Human IgG1
ETDWELLVFK
m/z 639.83-639.84
WYGLYFTYFK
m/z 693.84-693.85
FGFGQATTLK
m/z 534.79-534.80
VWDWWLYFRK
m/z 499.92-499.93
WSEQTTNLPK
m/z 601.80-601.81
FHWVYFYAYK
m/z 711.85-711.86
FLPFLTWAWK
m/z 654.36-654.37
FPFWYWMLFK
m/z 732.36-732.37
WFWVFPYHFK
m/z 728.36-728.37
Intensity
RT (min)

Supplementary Figure 26. EICs for peptides that were sequenced in 12ca5 selections, but not IgG_1 selections, and which do not contain the HA epitope. The presence of these peptides is detected in both 12ca5 and IgG_1 selections, indicating that these binders are non-specific, despite only having been sequenced from 12ca5 selections.

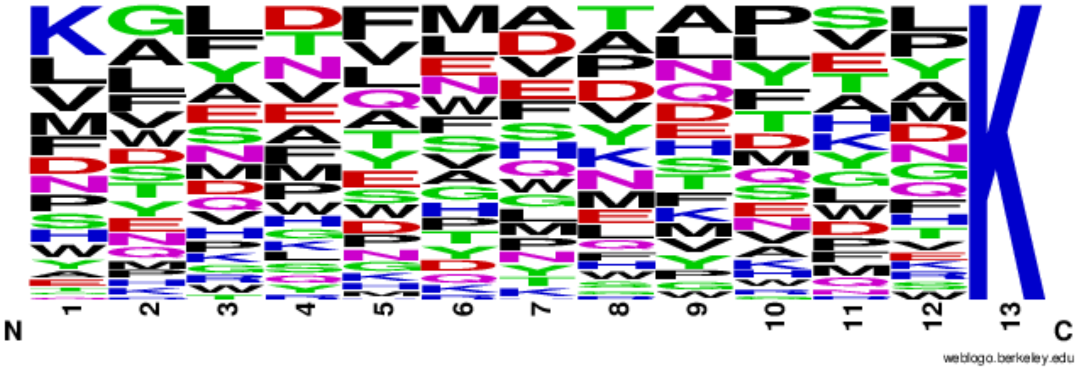

Supplementary Figure 27. nLC-MS/MS characterization of a $(X)_{12}K\text{-}CONH_2$ library, synthesized on 1.0 x 10^9 beads of 20 μM TentaGel resin, identifies 208 individual peptide sequences with an ALC score ≥ 80 from a theory of 559 beads cleaved. A positional frequency plot based on these 208 sequences is shown.

a)

Sequence: ETLVRPKPLL LKLLKSVGAQ KDTYTMKEVL FYLGQYIMTK RLYDEKQQHI VYCSNDLLGD LFGVPSFSVK EHRKIYTMIY RNLVV$^{\alpha}$-$\mathit{CONH_2}$

b)

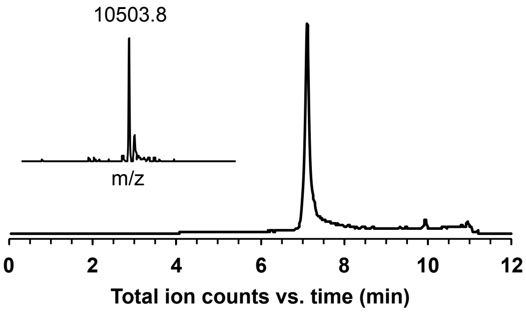

c)

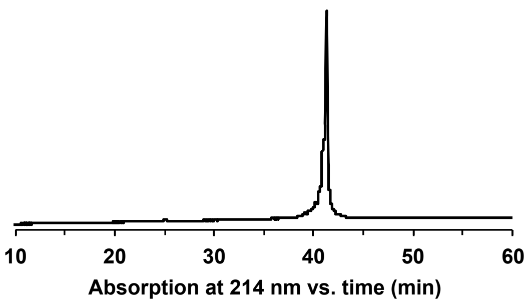

Supplementary Figure 28. a) Sequence of (25-109)MDM2. K36 is indicated in red. b) LC-MS characterization of (25-109)MDM2 K36(biotin) (monoisotopic mass: 10502.5 Da; found (by deconvolution): 10503.8 Da). c) Analytical HPLC characterization of (25-109)MDM2 K36(biotin).

a)

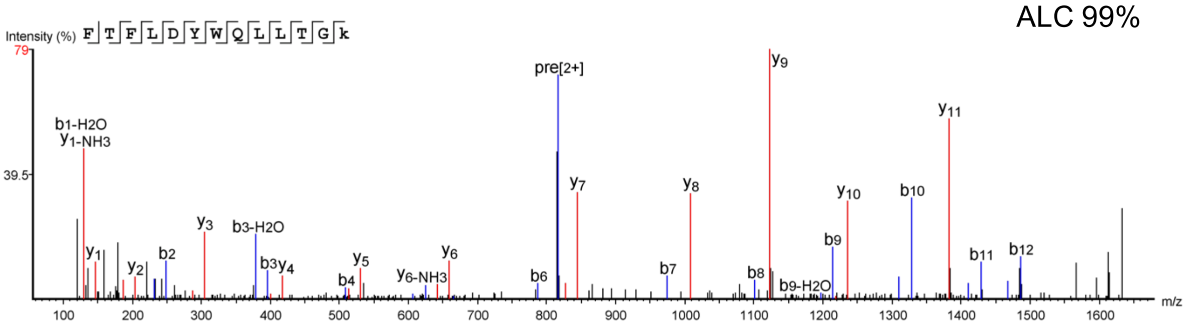

b)

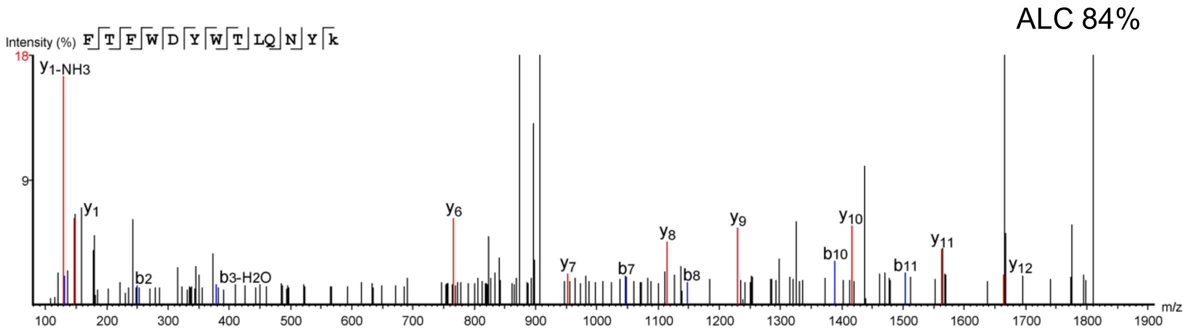

Supplementary Figure 29. Incomplete peptide backbone fragmentation during MS/MS results in potentially inaccurate sequence assignments. a) A complete ladder of y and b ions from MS/MS analysis enables the high-confidence sequence assignment of FT**F**LDY**W**QL**L**TGK, which contains the MDM2-binding, FXXXWXXL motif. b) Missing y4 and b9 ions prevent a high-confidence assignment of 'LQ' vs. 'QL' in the sequence FT**F**WDY**W**TLQNYK, which would contain the FXXXWXXL motif if the relative positions of Leu and Gln were inverted.

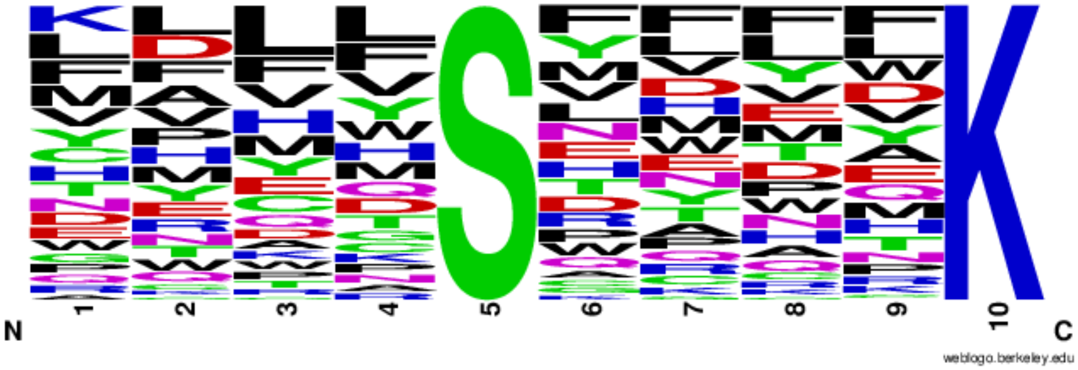

Supplementary Figure 30. nLC-MS/MS characterization of a $(X)_4(pS)(X)_4K\text{-}CONH_2$ library, synthesized on 2 x 10^8 beads of 30 µM TentaGel resin, identifies 617 individual peptide sequences with an average local confidence (ALC) score ≥ 80 from a theory of 475 beads cleaved. A positional frequency plot based on these 617 sequences is shown. Abbreviations: A = β-alanine; C = ornithine; D = D-aspartate; E = aminoadipic acid; F = cyclohexyl alanine; G = diaminobutyric acid; H = thiazolyl alanine; K = D-lysine (positions 1-9) or L-lysine (position 10); L = D-leucine; N = β-homothreonine; P = hydroxyproline; M = cyclopropylalanine; Q = D-glutamine; R = 4-aminophenylalanine; S = phosphoserine; T = β-homoserine; V = norvaline; W = 4-nitrophenylalanine; Y = 4-fluorophenylalanine.

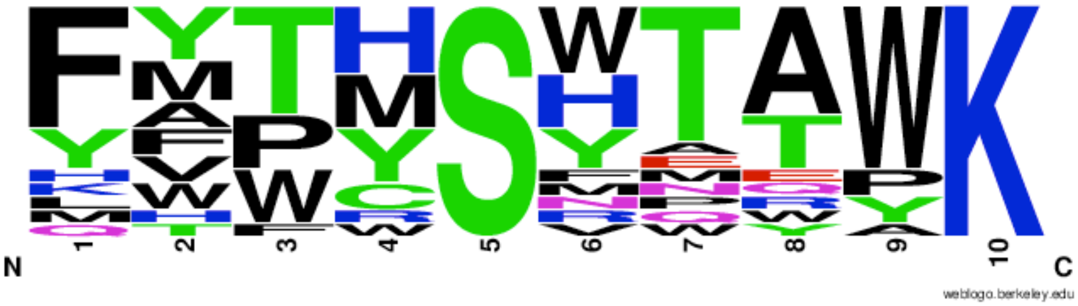

Supplementary Figure 31. Selections of a library comprised of non-canonical amino acids against 14-3-3γ identify sequences with prominent N-term and C-term motifs. A positional frequency analysis of all identified 14-3-3γ-unique peptides (17 in total) reveals a somewhat conserved FXT motif at the N-term, and a more prominent T(A/T)W motif near the N-term. Abbreviations: A = β-alanine; C = ornithine; D = D-aspartate; E = aminoadipic acid; F = cyclohexyl alanine; G = diaminobutyric acid; H = thiazolyl alanine; K = D-lysine (positions 1-9) or L-lysine (position 10); L = D-leucine; M = cyclopropylalanine; N = β-homothreonine; P = hydroxyproline; Q = D-glutamine; R = 4-aminophenylalanine; S = phosphoserine; T = β-homoserine; V = norvaline; W = 4-nitrophenylalanine, Y = 4-fluorophenylalanine.

a) 14.3.3.1 (EIC: m/z 734.28 – 734.29)

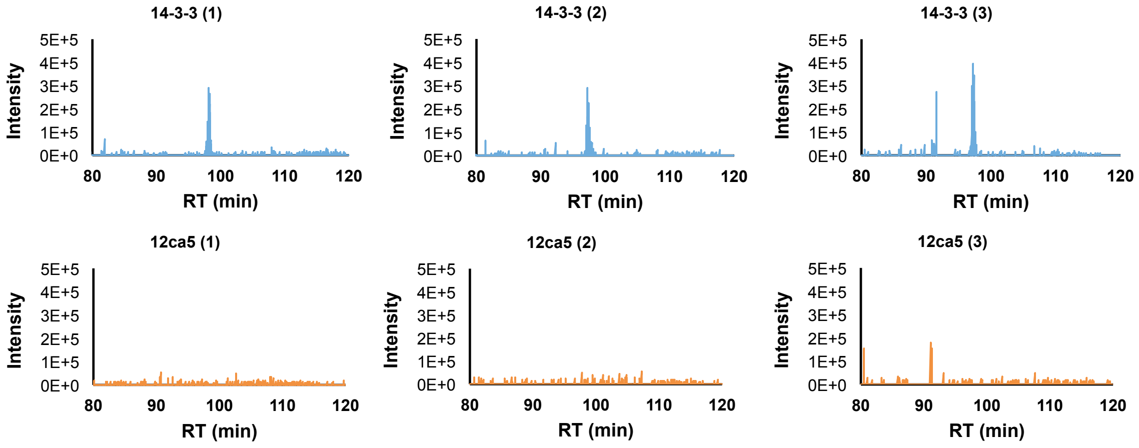

b) 14.3.3.6 (EIC: m/z 710.84 – 710.85)

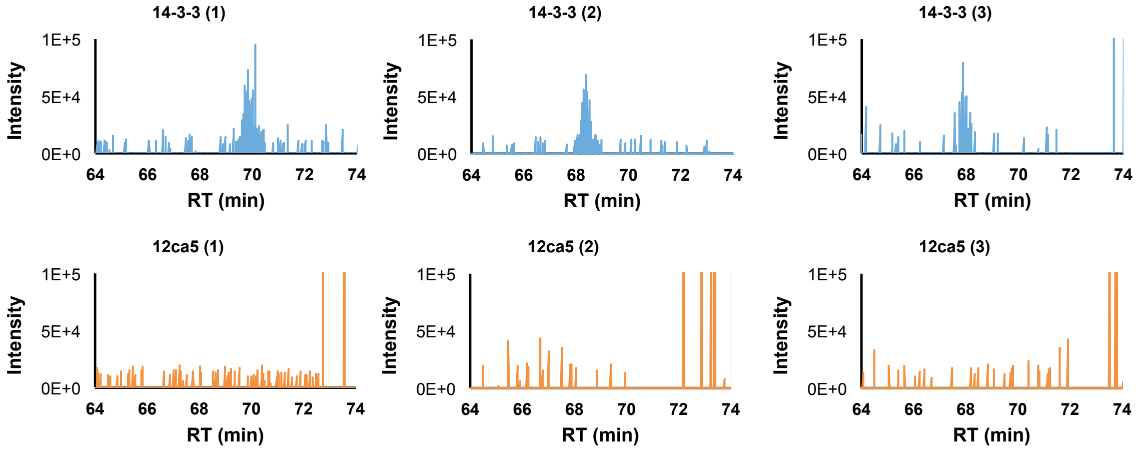

c) 14.3.3.12 (EIC: m/z 721.78 – 721.79)

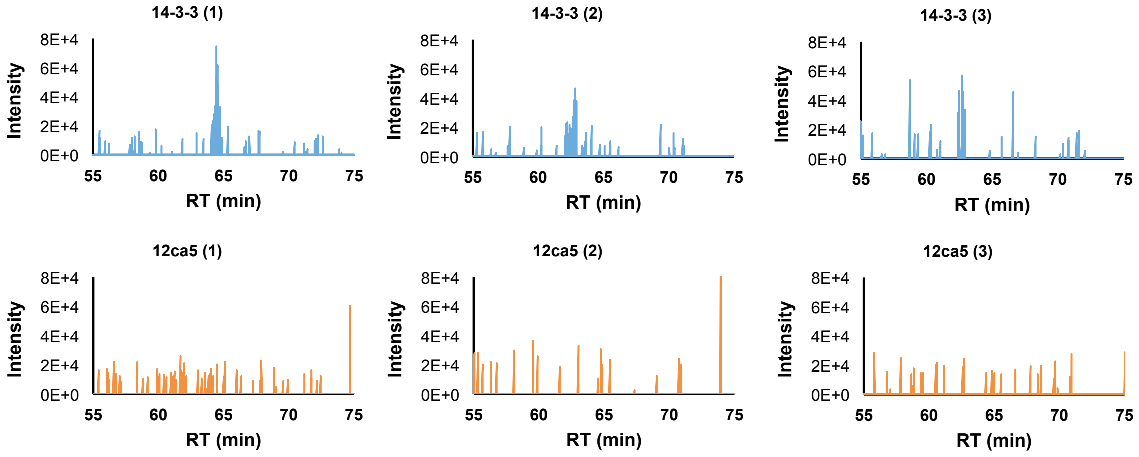

Supplementary Figure 32. Select putative 14-3-3γ binders are reproducibly pulled down in the presence of 14-3-3. Extracted ion chromatograms (EICs) of a subset of 14-3-3γ-unique sequences reveal these peptides were retained in each selection replicate against 14-3-3γ (blue traces). Signals for these peptides are also absent in the chromatograms from 12ca5 selections (orange traces), suggesting they were pulled down to the presence of 14-3-3. Three peptides are examined here: a) **14.3.3.1**, b) **14-3-3.6**, and c) **14-3-3.12** (**Supplementary Table 11**).

a)

Construct	Sequence										ALC (%)
**NB.1**	D-Lys	β-Ser	D-Gln	Thz	pSer	Aad	Thz	Nph	β-Thr	Lys	92
**NB.2**	Hyp	Nph	Thz	D-Asp	pSer	β-Ala	Cpa	Fph	Thz	Lys	86

b) NB.1 (EIC: m/z 476.85-476.86)

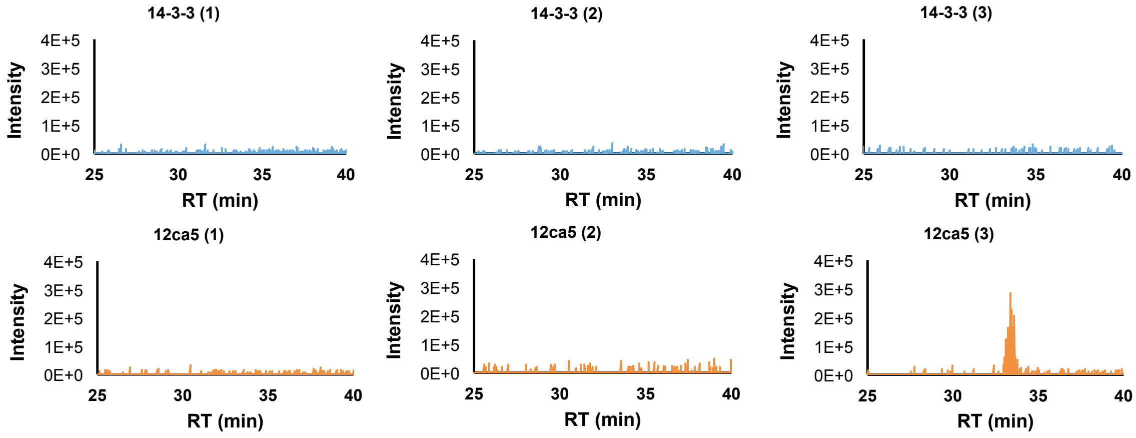

c) NB.2 (EIC 694.73 – 694.74)

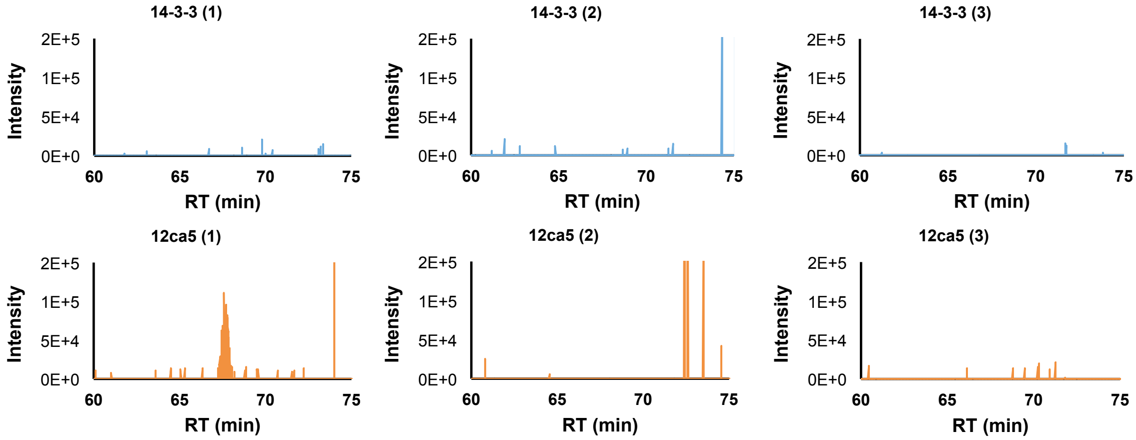

Supplementary Figure 33. 12ca5-unique sequences are not reproducibly pulled down and are likely selection artifacts. a) Peptide sequences identified from selections against 12ca5. b) EICs of putative 12ca5-binding peptide **NB.1** and c) **NB.2** from each selection replicate. In both cases, the putative 12ca5-binding peptide was only identified in one of three replicates, indicating it is likely an artifact from the selection rather than a true 12ca5 binder.

Construct: FITC-labeled **14-3-3.1**

Sequence: D-Leu–Nph–β-Ser–Nph–pSer–Nph–β-Ser–β-Ala–Nph–Lys–β-Ala–Lys(FITC)

Structure:

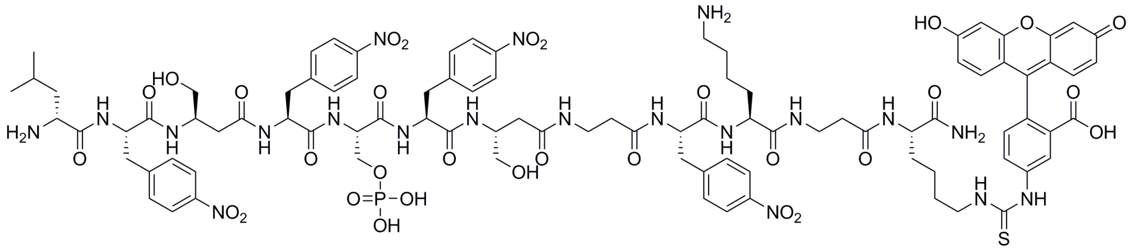

LC-MS trace:

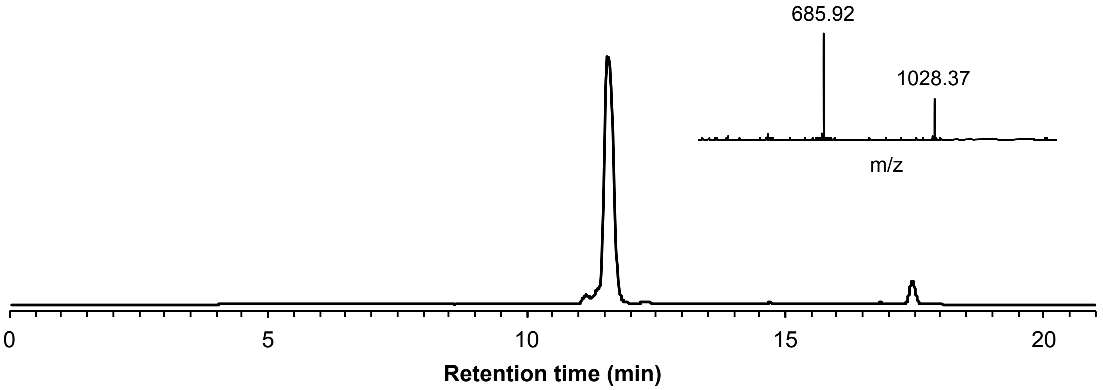

Supplementary Figure 34. LC-MS characterization of FITC-labeled **14-3-3.1** (monoisotopic mass: 2054.72 Da; found: 2054.72 Da). β-Ala spacer and FITC label are indicated in the sequence in blue.

Construct: FITC-labeled **14-3-3.6**

Sequence: Cha–Cha–β-Ser–Orn–pSer–Nph–β-Ser–β-Ser–Nph–Lys–β-Ala–Lys(FITC)

Structure:

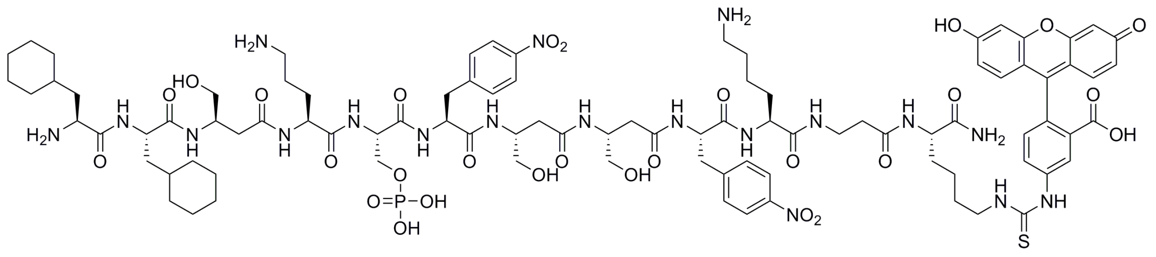

LC-MS trace:

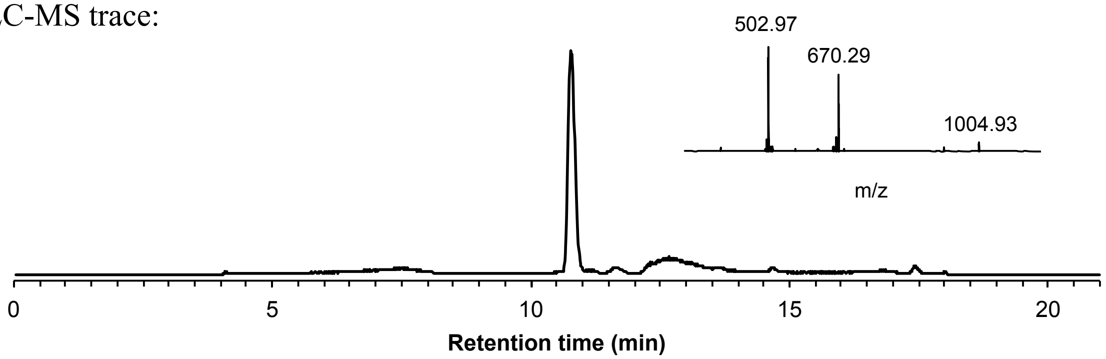

Supplementary Figure 35. LC-MS characterization of FITC-labeled **14-3-3.6** (monoisotopic mass: 2007.85 Da; found: 2007.85 Da). β-Ala spacer and FITC label are indicated in the sequence in blue.

Construct: FITC-labeled **14-3-3.12**

Sequence: D-Lys–Nva–Nph–Thz–pSer–Nph–β-Ser–β-Ala–Nph–Lys–β-Ala–Lys(FITC)

Structure:

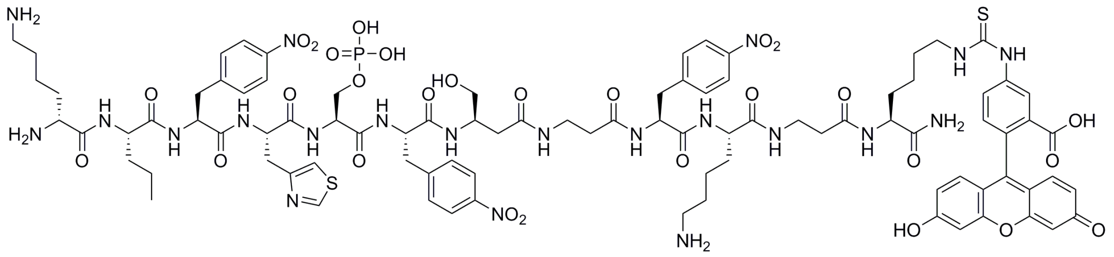

LC-MS trace:

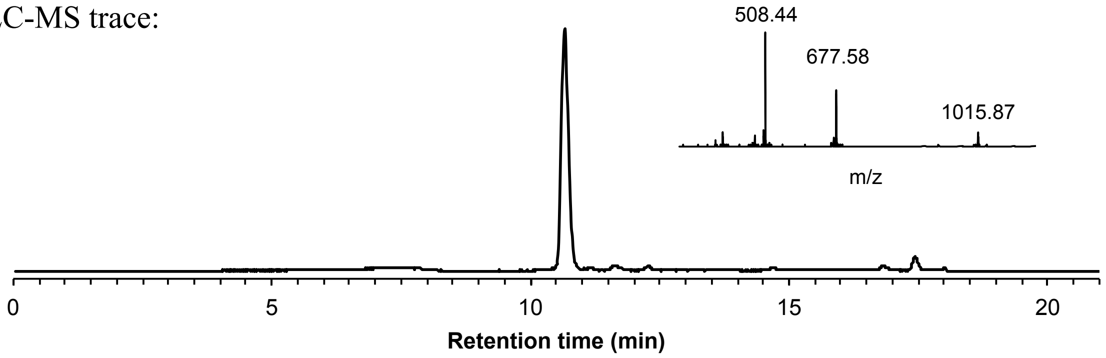

Supplementary Figure 36. LC-MS characterization of FITC-labeled **14-3-3.12** (monoisotopic mass: 2029.72 Da; found: 2029.72 Da). β-Ala spacer and FITC label are indicated in the sequence in blue.

Construct: FITC-labeled **NB.1**

Sequence: D-Lys–β-Ser–D-Gln–Thz–pSer–Aad–Thz–Nph–β-Thr–Lys– β-Ala–Lys(FITC)

Structure:

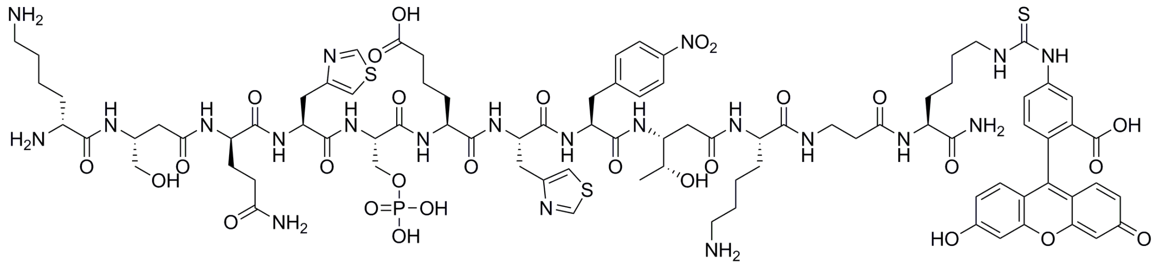

LC-MS trace:

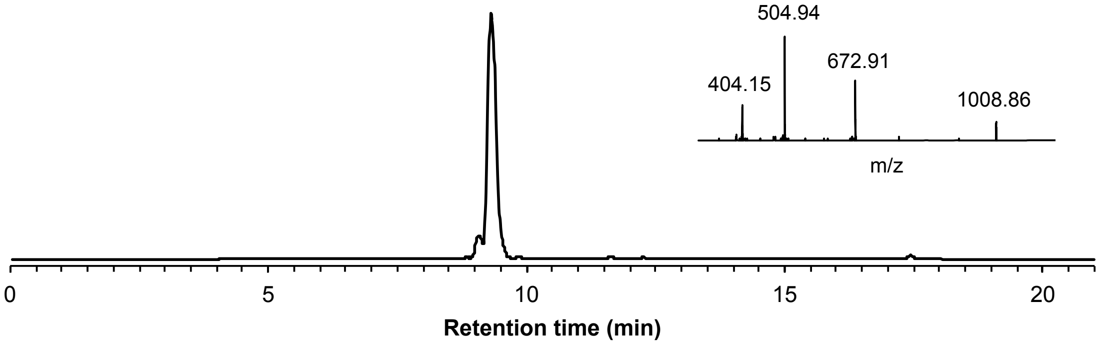

Supplementary Figure 37. LC-MS characterization of FITC-labeled **NB.1** (monoisotopic mass: 2015.70 Da; found: 2015.71 Da). β-Ala spacer and FITC label are indicated in the sequence in blue.

Construct: **14-3-3.1**

Sequence: D-Leu–Nph–β-Ser–Nph–pSer–Nph–β-Ser–β-Ala–Nph–Lys

Structure:

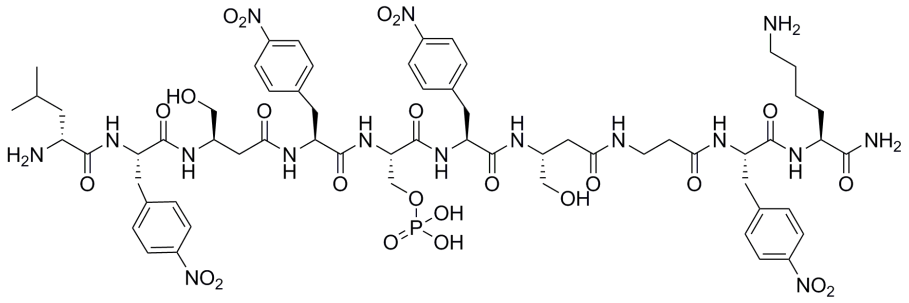

LC-MS trace:

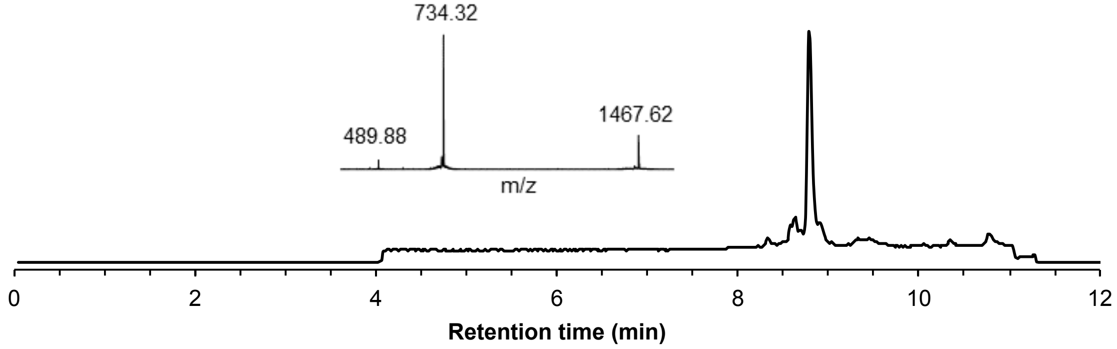

Supplementary Figure 38. LC-MS characterization of unlabeled **14-3-3.1** (monoisotopic mass: 1466.55 Da; found: 1466.61 Da).

Construct: **14-3-3.6**

Sequence: Cha–Cha–β-Ser–Orn–pSer–Nph–β-Ser–β-Ser–Nph–Lys

Structure:

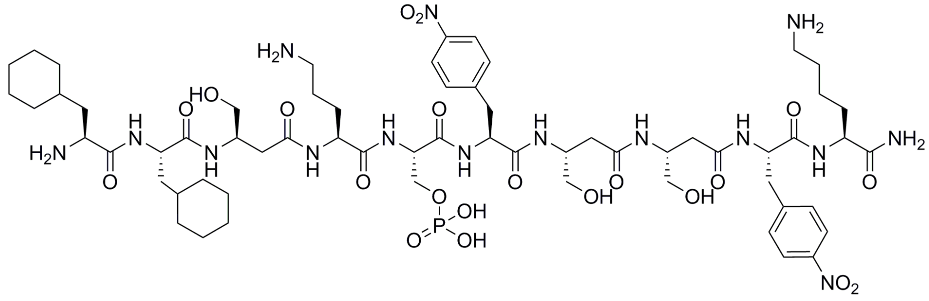

LC-MS trace:

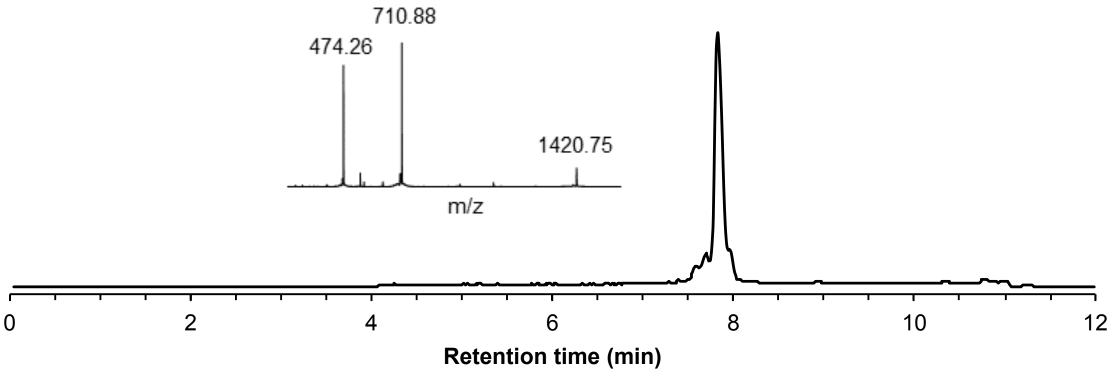

Supplementary Figure 39. LC-MS characterization of unlabeled **14-3-3.6** (monoisotopic mass: 1419.68 Da; found: 1419.74 Da).

Construct: **14-3-3.12**

Sequence: D-Lys–Nva–Nph–Thz–pSer–Nph–β-Ser–β-Ala–Nph–Lys

Structure:

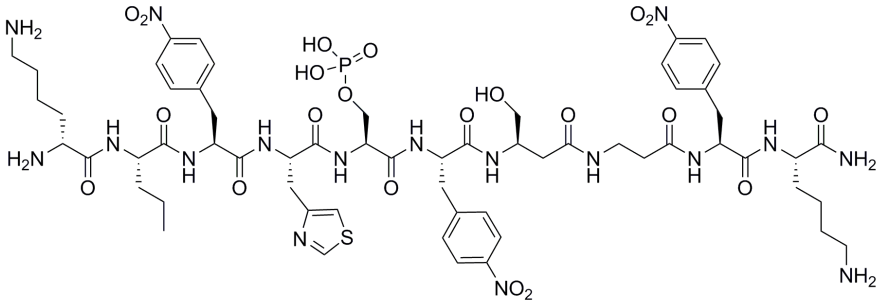

LC-MS trace:

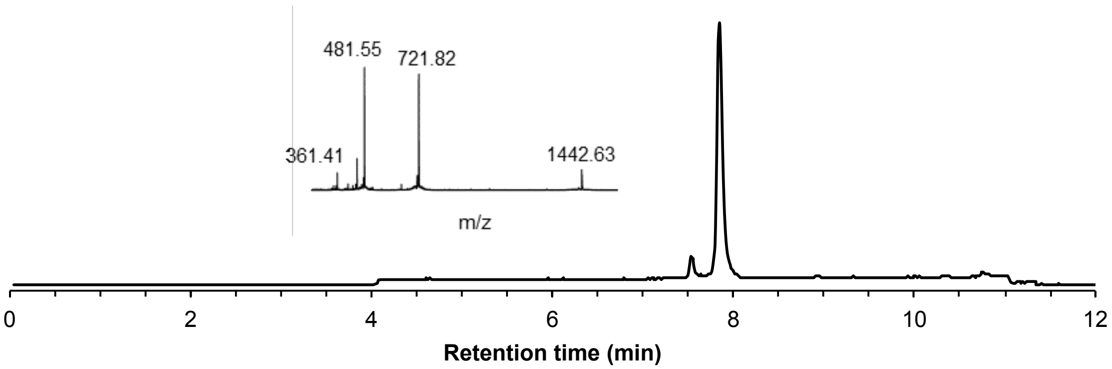

Supplementary Figure 40. LC-MS characterization of unlabeled **14-3-3.12** (monoisotopic mass: 1441.55 Da; found: 1441.62 Da).

Construct: **NB.1**

Sequence: D-Lys–β-Ser–D-Gln–Thz–pSer–Aad–Thz–Nph–β-Thr–Lys

Structure:

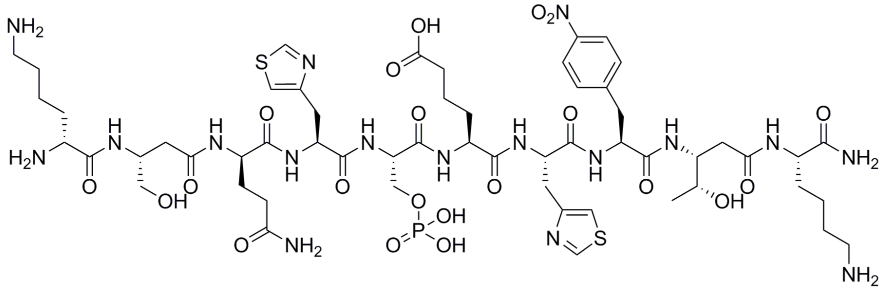

LC-MS trace:

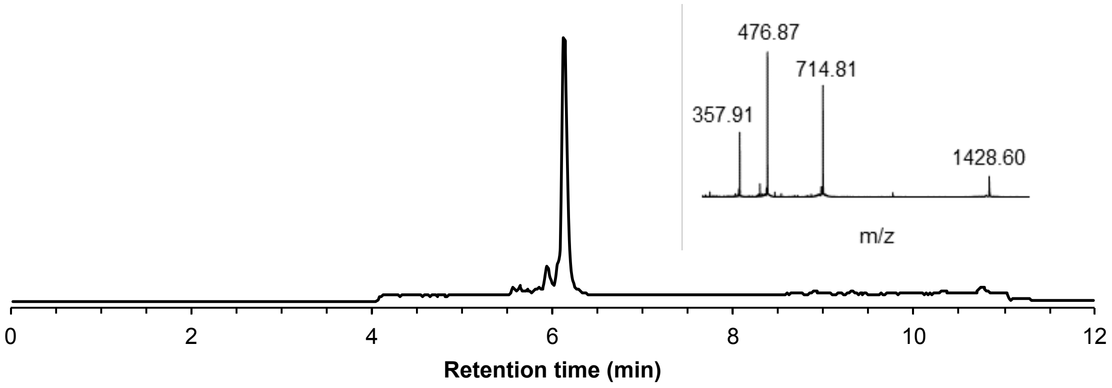

Supplementary Figure 41. LC-MS characterization of unlabeled **NB.1** (monoisotopic mass: 1427.54 Da; found: 1427.60 Da).

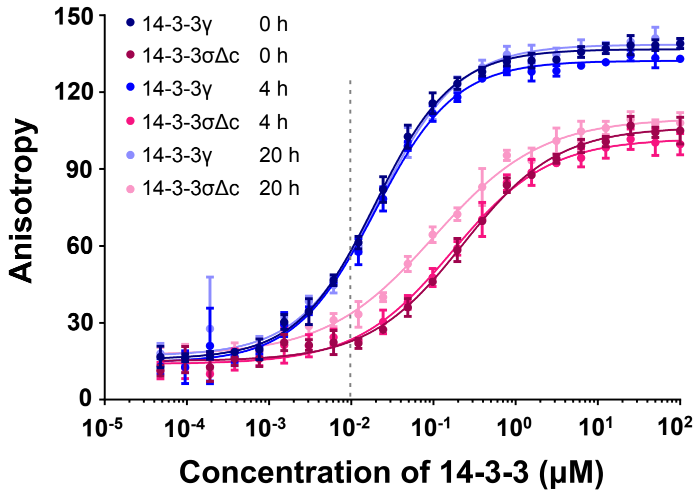

Time (h)	Isoform	K_d (nM)
**0**	**14-3-3γ**	19.5
**0**	**14-3-3σ**	228.9
**4**	**14-3-3γ**	19.4
**4**	**14-3-3σ**	169.4
**20**	**14-3-3γ**	21.9
**20**	**14-3-3σ**	101.5

Supplementary Figure 42. FITC-labeled **14-3-3.12** retains binding activity for 14-3-3σ as measured by fluorescence anistropy, with 5 to 12-fold reduced affinity relative to that for 14-3-3γ. Measurements were taken either immediately after, 4 h after, or 20 h after incubation of **14-3-2.12** with 14-3-3. Error bars correspond to standard error among three technical replicates.

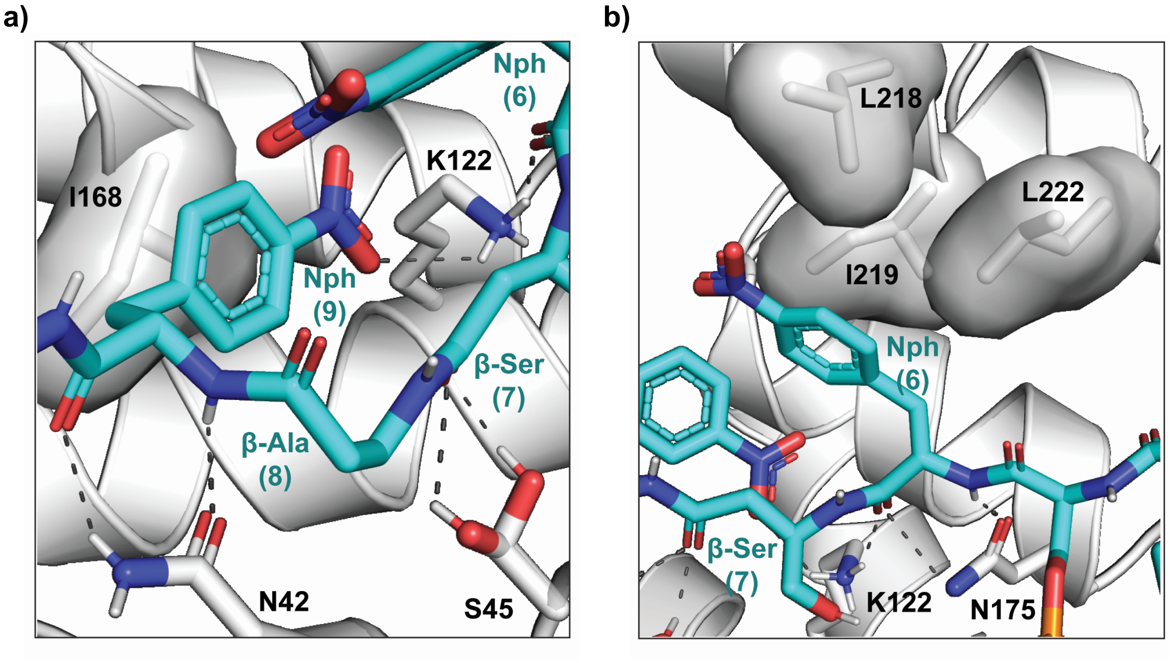

Supplementary Figure 43. a) 4-Nitrophenylalanine9 engages in an electrostatic interaction and/or H-bond with the $^{\varepsilon}NH_3$ group of Lys122 (N–O distance=3.2 Å), and makes a hydrophobic contact with Ile168. b) 4-Nitrophenylalanine6 interacts with the hydrophobic roof of the 14-3-3 binding groove, making hydrophobic contacts with Leu218, Ile219, and Leu222 of 14-3-3σ.

Supplementary Tables

Supplementary Table 1. Characterization of 12ca5-binding peptides by competition fluorescence polarization.

Binder	Source	IC_{50}	K_D
YPYDVPDYA	HA epitope	85 ± 7 nM	**3.9 ± 1 nM**
FDYEDYAEWK	Affinity selection	590 ± 190 nM	**25 ± 13 nM**
YPYDVPDYG	Ala9Gly mutant	3.6 ± 1 μM	**170 ± 70 nM**
Gypyeydwe	On-bead screen	62 ± 8 μM	**3.0 ± 0.85 μM**
ypyefdeph	On-bead screen	380 ± 25 μM	**18 ± 4 μM**

Model binders include the native HA epitope, a peptide identified from early affinity selection experiments, an Ala9Gly mutant of the native HA epitope, and sequences derived from the loop of 12ca5 xenoprotein binders identified in Gates, Z. P. *et al.* Xenoprotein engineering via synthetic libraries. *Proc. Natl. Acad. Sci. U.S.A.* **115**, E5298-E5306 (2018).[10]

Supplementary Table 2. Affinity-capture mass spectrometry identifies 12ca5-binding sequences in proportion with library size.

Library size	Peptide	ALC (%)	m/z	z	RT	Mass	ppm
2 x 10^6	MNDLVDYADK	99	599.7725	2	43.53	1197.5337	-2.7
2 x 10^7	YGMGFVVRMK	99	406.8743	3	27.04	1217.605	-3.2
	SFDKHDYAFK	99	419.539	3	27.38	1255.5986	-2.7
	PVDLKDYAEK	99	392.8788	3	37.51	1175.6187	-3.6
	PDLMDYAHFK	99	417.8641	3	48.87	1250.5754	-4.0
	EDLQDYAEFK	99	628.7915	2	57.68	1255.572	-2.8
	FDLADYATAK	99	557.2808	2	58.35	1112.5503	-3.0
	SFAMFSFFMK	99	637.289	2	74.14	1272.5671	-2.9
	PGMFWMWLWK	99	706.8334	2	95.67	1411.657	-3.3
	WLSFYTWLMK	99	695.3535	2	96.36	1388.6951	-2.0
	WPWGYLYFFK	99	703.3566	2	102.96	1404.7019	-2.3
	FYSFPWYWWK	98	754.8597	2	102.38	1507.7078	-1.9
	FFLDVMDFWK	97	681.8289	2	100.71	1361.6477	-3.2
	FDLEDYANEK	96	621.7835	2	57.45	1241.5564	-3.2
	WYPFFFYMWK	96	765.3535	2	101.75	1528.7002	-5.1
	TGDLADYAVK	94	526.275	2	42.13	1050.5347	0.8
	WWFYYLLNNK	94	723.3702	2	97.58	1444.7292	-2.3
	YFQPLMLWWK	93	713.8692	2	97.4	1425.7268	-2.1
	FFNVWWFRLK	92	481.2654	3	92.39	1440.782	-5.3
	FSDLEDYSTK	91	602.278	2	44.9	1202.5454	-3.4
	MGNWYFYWLK	89	711.8342	2	89.89	1421.6592	-3.8
	WMMFYWNLFK	89	748.8447	2	96	1495.678	-2.1
	PVDWPDYAAK	88	580.7887	2	52.13	1159.5662	-2.9
	PVDWDPAYAK	88	580.7891	2	52.75	1159.5662	-2.2
	WWFQYLNMFK	87	739.3563	2	95.78	1476.7012	-2.1
	NLWVFYSYWK	86	702.8549	2	94.76	1403.7026	-5.2
	MMDWPDYAVK	84	643.7786	2	51.32	1285.5471	-3.4
	SDLQDYSFMK	83	624.7806	2	46.82	1247.5493	-2.2
	FWQWWLQQFK	83	748.381	2	96.67	1494.7561	-5.7
	DYDRYADTFK	82	431.5335	3	51.22	1291.5833	-3.5
	WFLLWNDWWK	82	498.2538	3	88.27	1491.7451	-3.8
	DHWTFADLWK	81	439.8813	3	45.57	1316.6301	-6.1
2 x 10^8	MDMEDYAAMK	99	626.2372	2	21.55	1250.4617	-1.5
	MADLMDYAGK	99	573.2504	2	33.26	1144.4893	-2.6
	GMDVVDYAAK	99	542.2585	2	35.34	1082.5066	-3.7
	PDLEDYAVKK	99	392.8791	3	35.98	1175.6187	-2.8
	LEDVGDYAAK	99	540.2714	2	36.97	1078.5295	-1.3
	PADLPDYAQK	99	558.7861	2	37.23	1115.561	-3.0
	FDMQDYAYMK	99	671.7743	2	38.65	1341.5369	-2.1
	TSDLEDYAMK	99	594.2643	2	40.24	1186.5176	-3.0
	ATDMVDYAFK	99	588.2723	2	41.38	1174.533	-2.4
	LGDLMDYSEK	99	593.2747	2	41.38	1184.5383	-3.0
	ADLQDYAAVK	99	546.7863	2	43.23	1091.561	-2.8
	VFDLHDYASK	99	398.5351	3	45.44	1192.5876	-3.4
	WSDVEDYAAK	99	591.7742	2	46.92	1181.5352	-1.2
	MEDFVDYADK	99	624.2651	2	49	1246.5176	-1.6
	YEDLEDYAAK	99	608.279	2	49.59	1214.5454	-1.6
	SPDLEDYAYK	99	600.2814	2	50.67	1198.5505	-1.9

ALQDLQDYAK	99	582.3048	2	51.28	1162.5981	-2.6
FMMDLEDYAK	99	647.2767	2	52.3	1292.5417	-2.2
LDLPDYAFHK	99	406.5477	3	53.29	1216.624	-2.3
ADWRDYADLK	99	417.8743	3	54.23	1250.6042	-2.6
WFDTTDYADK	99	630.7792	2	54.68	1259.5459	-1.7
LTDLQDYSFK	99	614.8128	2	55.26	1227.6135	-2.0
YEDVLDYADK	99	615.2884	2	56.05	1228.561	1.0
LNDLPDYSFK	99	605.8074	2	56.54	1209.603	-2.2
YFDVEDYAAK	99	610.2838	2	57.97	1218.5557	-2.1
PWDVQDYAYK	99	642.3043	2	58.06	1282.5981	-3.2
FADWEDYANK	99	629.2789	2	58.03	1256.5461	-2.4
LMDLEDYAFK	99	630.3009	2	62.3	1258.5903	-2.4
VDLEDYANFK	99	606.7968	2	62.97	1211.5823	-2.6
FFDVQDYAYK	99	647.8066	2	65.85	1293.603	-3.3
FFDLEDYSVK	99	631.3069	2	71.36	1260.6025	-2.5
YFWWQYMMYK	99	788.84	2	75.7	1575.668	-1.6
WFDAQLLVFK	99	633.3539	2	87.77	1264.6968	-2.8
YADAHDYAFK	98	400.5195	3	32.45	1198.5408	-3.4
PLVDVHDYAK	98	385.8754	3	40.45	1154.6084	-3.6
LDKVDYADWK	98	417.8819	3	45.44	1250.6294	-4.3
EFDLHDYATK	98	413.1983	3	45.91	1236.5774	-3.4
WDLEDYSEHK	98	440.8651	3	47.13	1319.5781	-3.5
QADVWDYAAK	98	583.2838	2	52.01	1164.5564	-3.0
YFDVADYASK	98	589.2786	2	56.58	1176.5452	-2.2
QDFPDYADFK	98	622.7811	2	61.08	1243.551	-2.7
EVDLVDYAAK	98	561.2945	2	65.07	1120.5764	-1.8
EFDLADYSWK	98	636.7969	2	74.66	1271.5823	-2.4
KGDLHDYAFK	97	398.2073	3	25.99	1191.6035	-2.9
LGDMPDYAAK	97	548.26	2	27.34	1094.5066	-1.0
MDEDYAAFQK	97	616.764	2	41.17	1231.5178	-3.6
ALDVPDYAGK	97	524.2769	2	44.54	1046.5396	-0.3
DVHDYAHFWK	97	439.5427	3	50.09	1315.6099	-2.7
VDVTDYAEFK	97	593.2922	2	56.81	1184.5713	-1.2
LFDVEDYADK	97	607.2896	2	60.86	1212.5662	-1.3
YTDLWDYAEK	97	651.8024	2	67.33	1301.5928	-1.9
YWPFLWYAMK	97	710.3473	2	90.39	1418.6846	-3.2
LMDFQDYAHK	96	428.1992	3	37.77	1281.5813	-4.3
LDKPDYAQFK	96	408.5505	3	38.33	1222.6345	-3.9
LTDVPDYASK	96	554.2863	2	38.55	1106.5608	-2.6
EVEDYASTMK	96	594.2637	2	39.58	1186.5176	-3.9
ALDLKDAYAK	96	553.8118	2	40.57	1105.613	-3.6
WTDLPDYSHK	96	420.8703	3	40.62	1259.5935	-3.4
KLENDYAELK	96	407.8857	3	42.41	1220.6401	-4.0
MEDAADYAWK	96	607.759	2	43.56	1213.5073	-3.2
WDDFKDYADK	96	434.5298	3	48.05	1300.5723	-3.6
EDLPDYAFPK	96	597.2949	2	58.3	1192.5764	-1.0
WFWYTHPQFK	96	719.8552	2	63.54	1437.6982	-1.7
DLHDYAHTLK	95	404.5423	3	32.42	1210.6094	-3.5
EVDVKDYAYK	95	410.2103	3	37.81	1227.6135	-3.6
LGDMFDYAAK	95	573.2719	2	44.15	1144.5222	6.1
WQDMDPYAFK	95	658.2911	2	52.13	1314.5703	-2.0
DGVMDYAAPK	94	541.2529	2	27.33	1080.491	0.2

	LVDMDMYAMK	94	632.2737	2	28.45	1262.5344	-1.2
	TMLDMDPYAK	94	608.2714	2	32.6	1214.5312	-2.5
	YVDKVDYAFK	94	416.2185	3	40.85	1245.6394	-4.5
	EDMPDYAVWK	94	634.7835	2	52.89	1267.5542	-1.4
	FDVWDYAQVK	94	635.3154	2	73.66	1268.6189	-2.1
	VMLDKVDYAK	93	399.5481	3	30.86	1195.6272	-4.0
	VEGDLPDYAK	93	553.2777	2	40.46	1104.5452	-3.9
	FDYEDYARAK	93	426.2018	3	41.61	1275.5884	-3.8
	EVADYADTVK	93	555.2762	2	53.65	1108.54	-1.9
	MLDVYDSFWK	93	659.8082	2	67.18	1317.6064	-3.5
	ELDMHDYAVK	92	412.5279	3	25.75	1234.5652	-2.6
	DLADYADKAK	92	554.784	2	37.8	1107.5559	-2.2
	WDVMDYAKAK	92	414.5361	3	39.32	1240.5911	-3.7
	WSDLKDYAAK	92	399.2063	3	40.91	1194.6033	-5.3
	YMDLVDYESK	92	639.2874	2	47.14	1276.5645	-3.3
	EGVQDYASWK	92	591.2805	2	49.56	1180.5513	-4.2
	VVDLADYSYK	92	586.3027	2	53.58	1170.592	-1.0
	FDVPDYAYYK	92	640.3033	2	64.01	1278.592	0.1
	MPDVDMYAAK	91	586.2582	2	33.01	1170.5049	-2.6
	HVDWKDYAEK	91	430.5456	3	36.34	1288.6201	-3.9
	FADADPYAYK	91	580.2725	2	44.86	1158.5344	-3.5
	DKQDYAALFK	91	599.3159	2	49.57	1196.6189	-1.3
	VYDVEDYSYK	91	640.2941	2	49.97	1278.5769	-2.6
	WDQHDYAFLK	91	441.2174	3	55.99	1320.625	4.1
	EVADYADMFK	91	602.2693	2	60.99	1202.5278	-3.2
	WFWFGWWMPK	91	743.3563	2	93.08	1484.6853	8.5
	YHDLPDYAHK	90	419.8707	3	26.02	1256.5938	-2.7
	VDKEDYAMLK	90	409.5404	3	26.83	1225.6013	-1.6
	LDDMENYAVK	90	606.7806	2	30.88	1211.5493	-2.1
	MNDLPDYATK	90	591.7748	2	36.49	1181.5386	-2.9
	YPYDVDPYAK	90	615.2946	2	53.21	1228.5764	-1.4
	YYDLPDYAFK	90	647.3102	2	68.1	1292.6077	-1.3
	PVDEMDYAEK	89	606.2648	2	24.97	1210.5176	-2.1
	LMRDVPDYSK	89	413.5437	3	26.25	1237.6125	-2.7
	FVQDMDEYAK	89	630.7797	2	32.16	1259.5493	-3.5
	YFDKVDYAEK	89	426.2108	3	36.71	1275.6135	-2.4
	GPDMDEYAFK	89	594.255	2	36.97	1186.4963	-0.7
	HDLPDYAYYK	89	642.3045	2	44.78	1282.5981	-2.9
	DYHDSFHWYK	89	466.2057	3	48.2	1395.5996	-3.1
	MFDLRDYAAK	89	415.5401	3	49.53	1243.6018	-2.6
	FWDLMDYANK	89	659.2991	2	66.1	1316.5859	-1.7
	MLKDVDYSYK	88	426.2114	3	32.74	1275.6169	-3.6
	GPDVEDYAVK	88	546.2699	2	40.24	1090.5295	-3.9
	FDFDLPDYSK	88	623.2931	2	73.14	1244.5713	0.3
	YDVVWYSESK	87	425.5369	3	36.22	1273.5979	-7.1
	TMTDLQDYAK	87	600.7798	2	38.25	1199.5493	-3.5
	YLDTHDAYFK	87	424.5388	3	42.88	1270.5981	-2.8
	WMDLPDAYTK	87	627.7938	2	54.45	1253.575	-1.5
	YFMDWPDYAK	87	675.7944	2	63.97	1349.575	-0.5
	FADLHDYESK	86	408.5265	3	35.5	1222.5618	-3.3
	NDLHDYSYLK	86	422.8735	3	40.98	1265.604	-4.2
	LPDHHDYFSK	85	419.8711	3	27.22	1256.5938	-1.8

	PTDLQDFSNK	85	582.2871	2	35.6	1162.5618	-1.8
	FFDMKDYAEK	85	436.8674	3	36.41	1307.5857	-4.0
	TQDLKDYAFK	85	409.8821	3	40.3	1226.6294	-4.1
	LQDVEDVYSK	85	597.8018	2	40.86	1193.5928	-3.2
	PVDWKDYEAK	85	417.2105	3	46.16	1248.6138	-3.2
	TTWDVEDAYK	85	613.7865	2	56.7	1225.5615	-2.5
	EVWDYADPDK	85	618.7792	2	69	1235.5459	-1.7
	FFWYYYGPWK	85	728.3517	2	89.06	1454.6812	5.3
	WLMAFWPTWK	85	690.8472	2	91.59	1379.6848	-3.6
	KDVHDYAYLK	84	417.5548	3	27.41	1249.6455	-2.3
	YPDVHDAYVK	84	402.5348	3	40	1204.5876	-4.2
	PWKDNEYSFK	84	438.2135	3	40.62	1311.6248	-4.6
	KAQNLQDYAK	84	393.2144	3	43.78	1176.625	-3.2
	KSWDPADYAK	84	393.863	3	45.06	1178.572	-4.0
	WPDKADYAWK	84	426.8791	3	50.01	1277.6194	-3.2
	FWNDAEDYAK	84	629.2795	2	53.37	1256.5461	-1.3
	FWYYTPWWSK	84	731.8497	2	88.74	1461.687	-1.5
	KDVYDRMQQK	83	442.558	3	21.9	1324.6558	-2.7
	MMDVADYSSK	83	589.2455	2	25.05	1176.479	-2.1
	PVPDMDPYAK	83	574.2748	2	31.35	1146.5378	-2.4
	DLPDYAVSRK	83	581.8119	2	37.9	1161.6143	-4.3
	DMDVDRYAWK	83	438.5354	3	44.33	1312.5869	-1.9
	VVDVNGYEYK	83	592.7996	2	44.74	1183.5874	-2.4
	MDLHDYADFK	83	423.8558	3	45.75	1268.5496	-3.1
	WNDFKDYADK	83	434.2006	3	47.94	1299.5884	-6.5
	YLDWADFSEK	83	636.7968	2	66.25	1271.5823	-2.6
	WMWNAWYWPK	83	741.8414	2	91.77	1481.6704	-1.4
	DLHDYADLSK	82	588.2863	2	43.31	1174.5618	-3.2
	LMDMPDYANK	81	614.7694	2	28.9	1227.5264	-1.7
	HDWNEYAAAK	81	602.2785	2	36.11	1202.5469	-3.7
	VFDVHDEYSK	81	413.1984	3	37.4	1236.5774	-3.3
	SMDLEDYAMK	81	617.257	2	40.8	1232.5054	-4.9
	ADHSEYSFFK	81	410.523	3	43.39	1228.5513	-3.4

List of all sequences (ALC score ≥ 80) identified from selections performed with 2 x 10^6, 2 x 10^7, and 2 x 10^8-member libraries against 12ca5 (one replicate shown). Sequences are separated by the library they were discovered from and listed by decreasing ALC score.

Supplementary Table 3. Replicate selections from a 2 x 10^8-member library identify similar populations of 12ca5-binding peptides.

Number of replicates in which a DxxDY(A/S)-containing sequence was identified	Count	Percent
3	46	30.7
2	43	28.7
1	61	40.7

Out of three replicate selections from a 2 x 10^8-member library for 12ca5 binding, approximately 60% of DXXDY(A/S)-containing sequences are identified in multiple replicates, suggesting that similar (although not identical) populations of 12ca5-binding peptides are reproducibly identified. In total, 150 DXXDY(A/S)-containing sequences were obtained.

Supplementary Table 4. Use of a precursor selection threshold modulates enrichment based on sample loading.

Peptide	ALC (%)	RT	Mass	ppm	MS signal (8 fmol)	Max NL (75 fmol)
MNDLVDYADK	99	41.83	1197.5337	-7.7	2.40E+05	2.45E+06
WDFWYFYGYK	99	92.25	1472.6553	-8.3	2.02E+04	2.07E+05
WFNFWGWEMK	99	85.67	1444.6387	-6.4	2.50E+04	1.61E+05
PDVHDYTWGK	99	35.24	1215.5671	-7.5	1.67E+04	9.83E+04
YLMYPWWWLK	99	88.72	1499.7424	-6	1.34E+04	9.65E+04
FYQWYYSWFK	99	85.74	1515.6975	-5.9	1.46E+04	8.16E+04
FLWYDYWFYK	99	94.22	1528.718	-6.9	1.44E+04	7.82E+04
WYWMYYWPSK	99	79.77	1523.6697	-7.2	1.33E+04	6.10E+04
YTGFTWFWWK	99	91.77	1419.6765	-6.4	9.30E+03	5.75E+04
NFQWWMMWFK	98	93.56	1533.6687	-7.6	1.89E+04	7.75E+04
FFWWNMLWPK	98	91.98	1468.7114	-5.8	7.54E+03	6.84E+04
FWNFFYSWYK	98	94.75	1485.687	-5.2	6.71E+03	6.46E+04
WDFWYFYYGK	97	89.85	1472.6553	-6.5	2.54E+04	1.11E+05
DMWPWWWMWK	96	92.51	1581.6687	-6.3	9.28E+03	7.25E+04
WWEMWPWDYK	96	86.16	1540.6599	-6.5	1.10E+04	5.98E+04
FTPWWLWGFK	95	94.93	1365.7021	-6.5	1.47E+04	7.94E+44
AFQGWWWYYK	95	79.68	1432.6716	-6.5	9.50E+03	6.03E+04
PYYYPVWWWK	94	84.9	1485.7234	-6.4	1.21E+04	6.32E+04
MWAYWFSWPK	94	84.61	1415.6484	-6.2	1.04E+04	5.87E+04
WWFDWAYLAK	94	97.23	1383.6765	-6.5	5.26E+03	5.62E+04
YFTWPAYWWK	91	87.5	1445.6921	-8	1.26E+04	6.34E+04
HPWFWWLTYK	90	77.3	1461.7346	-5.7	1.53E+04	1.01E+05
FWWQYATMWK	90	81.90	1460.6699	-9.0	1.42E+04	5.45E+04
WPYPFWWLHK	89	74.77	1457.7397	-6.5	8.48E+03	8.07E+04
WWQWWMAGYK	89	77.07	1455.6545	-6.5	7.60E+03	5.27E+04
FWWLMYPWGK	88	92.44	1427.6848	-8.3	1.77E+04	5.60E+04
FWYFPWAWQK	88	91.3	1456.708	-7.1	8.46E+03	5.54E+04
NYWWFYMFPK	87	87.32	1495.6748	-7.3	9.70E+03	8.02E+04
ENDWQDYSHK	87	28.73	1319.553	-7.5	1.10E+04	6.48E+04
WMQYWMWEFK	86	81.78	1564.6631	-8.3	8.94E+03	5.27E+04
YYWVWFWNGK	85	88.53	1446.6873	-4.2	1.05E+04	6.64E+04
PYFWMYYDWK	85	80.32	1512.6536	-6.4	9.56E+03	6.03E+04
WGWWFEEMFK	84	86.53	1459.6384	-7.4	8.70E+03	5.11E+04
FYWPMWFFPK	81	94.77	1462.6897	-6.5	8.76E+03	7.39E+04
LVVFAWMYNK	81	63.84	1284.6689	-6.7	1.02E+04	5.86E+04

At low sample loading, use of a precursor selection threshold of 5 x 10^4 yielded only one identified sequence (MNDLVDYADK). At high sample loading, two additional DxxDY-containing sequences were identified, along with 32 non-motif-containing sequences. MS signal is reported as the apex of extracted ion chromatograms.

Supplementary Table 5. Selections against 12ca5 identify a decreasing number of motif-containing sequences as library size is increased from 10^8 to 10^9.

Library size	[Library member]	ALC score cutoff	Total sequences identified	Motif-containing sequences	Percent
10^8	10 pM	≥ 80	257	183	71.2
10^8	2 pM	≥ 80	242	131	54.1
10^9	2 pM	≥ 80	34	21	61.8

An approximate 9-fold drop in the number of 12ca5-binding sequences is observed from one-pot selections of a 10^9-member library relative to a 10^8-member library. Selections were performed near the solubility limit of the libraries (1-2 mM), and variable member concentration.

Supplementary Table 6. Increasing scale of 10^9-member library selections does not restore recovery of 12ca5-binding peptides.

Library size	Starting material per member	Total sequences identified	Motif-containing sequences	Percent
	1 fmol	7	4	57.1
10^9	5 fmol	15	10	66.7
	12 fmol	8	7	87.5

An increase in the amount of each library member present in the selection did not yield an increase in the number of sequences bearing the characteristic DXXDY(A/S) motif, suggesting that the decreased number of 12ca5-binding sequences identified is not due to material limitation.

Supplementary Table 7. Ten-fold increase in 12ca5 in selections from a 10^9-member library does not restore recovery of 12ca5-binding peptides.

	0.1 μM 12ca5	1 μM 12ca5
**Amount of 12ca5 (nmol):**	0.13	1.3
**DXXY(A/S)-containing sequences:**	26	0
**Total sequences:**	52	18
**Percent motif-containing:**	50%	0%

Increasing the amount and concentration of 12ca5 10x (from 0.1 nmol/0.1 μM to 1 nmol/1μM) abrogated identification of DXXDY(A/S)-containing peptides.

Supplementary Table 8. Peptides identified in presence of high exogenous competitor exhibit generally stronger signal intensities than those identified only in the presence of low exogenous competitor.

Peptide	ALC (%)	RT	Mass (Da)	Ppm	MS signal (1 nM HA)	Identified in presence of 100 nM HA?	Average Max NL (± Std. Dev.)
MDMEDYAAMK	99	20.93	1250.4617	-4.5	7.25E+05	Yes	1.17E+06 ± 4.6E+05
PVDMEDYAEK	99	23.17	1210.5176	-3.1	1.17E+06		
TWDRPDYADK	99	27.89	1264.5837	-4.9	2.17E+06		
YFDVEDYAAK	99	47.82	1218.5557	-4.8	9.64E+05		
LMDLEDYAFK	99	51.71	1258.5903	-5.5	1.30E+06		
YEDLEDYAAK	98	40.25	1214.5454	-5.3	6.85E+05		
LTDVPDYASK	93	32.75	1106.5608	-2.9	1.20E+06		
PTDVMDYASK	99	25.9	1140.5122	-2.7	2.94E+05	No	5.69E+05 ± 2.6E+05
HDWQDYAAAK	99	31.7	1202.5469	-4.1	6.90E+05		
TMTDLQDYAK	99	33.39	1199.5493	-3.9	3.70E+05		
MSDLEDYAMK	99	34.75	1232.5054	-2.8	2.52E+05		
HDLPDYAYYK	99	37.85	1282.5981	-5	8.43E+05		
SPDLEDYAYK	99	42.37	1198.5505	-4.7	7.16E+05		
YVDWQDYADK	98	48.38	1300.5725	-6	5.42E+05		
LGDYADYAAK	96	35.36	1084.5188	-5.5	1.96E+05		
GPDVEDYAVK	95	34.96	1090.5295	-6.1	9.65E+05		
LNDLPDYAYK	91	48	1209.603	-5.7	7.00E+05		
HADMWDYADK	88	33.55	1265.5134	-4.6	3.76E+05		
TDVRDYADYK	81	32	1243.5833	-5.2	8.85E+05		

Seven DXXDYA-containing peptides were identified in the presence of 100 nM exogenous HA epitope, compared to 81 identified in the presence of 1 nM exogenous HA epitope (12 were randomly selected for analysis here). On average, peptides identified in the presence of greater exogenous competitor exhibited higher signal intensities than those only identified under less stringent conditions. MS signal is reported as the apex of extracted ion chromatograms.

Supplementary Table 9. Sequence subtraction from side-by-side selections yields modestly improved enrichments.

Processing method	Total sequences identified (12ca5)	Motif-containing sequences	Percent
ALC ≥ 80 (no subtraction)	198	133	67.2
ALC ≥ 80 (with subtraction)	181	133	73.5

Shown are the number of DXXDY(A/S)-containing sequences and total sequences identified from side-by-side selections of a 2 x 10^8-member library against 12ca5 and polyclonal human IgG_1. Selections were performed in triplicate, and unique sequences from the sum of these technical replicates are indicated. Subtracting out non-specific sequences (those identified in both conditions) improves identification of motif-containing sequences as a fraction of the total.

Supplementary Table 10. Multi-pot selections against MDM2 identify p53-like peptides from a 10^9-member library.

Name	Scan	Peptide	ALC (%)	m/z	z	RT	Mass	ppm
**1.1**	17209	PSFHAVMWLKSFK	99	394.9648	4	53.04	1575.8384	-5.1
**1.2**	38696	KADPWWLETFWWK	80	597.9682	3	85.91	1790.8933	-5.9
**2.1**	43700	LLFDFTYAKWLYK	95	569.6469	3	89.84	1705.9231	-2.5
**2.2**	18425	HRPEYAVSVLAAK	86	480.6077	3	45.79	1438.8044	-2.3
**2.3**	31504	YSFHYWWTQLNEK	80	900.9277	2	68.67	1799.842	-0.6
**3.1**	25254	VSHYWTAYVQWYK	92	865.4241	2	65.52	1728.8413	-4.5
**3.2**	41789	PSFLDYWKVVFYK	90	564.3014	3	94.44	1689.8918	-5.6
**3.3**	46448	WWLWAYSYFQPWK	88	930.4522	2	99.85	1858.8984	-4.6
**3.4**	47164	FAWQPFQWWYNWK	87	943.4470	2	101.15	1884.8889	-5.0
**3.5**	26639	KTFVEYWNELRVK	80	570.9803	3	67.94	1709.9253	-3.7
**4.1**	49230	FTFLDYWQLLTGK	99	815.9340	2	110.13	1629.8555	-1.2
**4.2**	37222	LTTFFDYWNELYK	97	869.9229	2	101.54	1737.8403	-5.2
**4.3**	43167	FYALEYWQNFTDK	82	862.4064	2	97.87	1722.8042	-3.4
**4.4**	8578	ESMMYSHPQLFDK	80	548.5768	3	45.54	1642.7119	-2.0
**5.1**	28399	FGGYWWSQLYTYK	95	849.4065	2	93.05	1696.8037	-3.1
**5.2**	32707	FTFWDYWTLQNYK	84	905.9298	2	103.55	1809.8516	-3.6

List of all 16 sequences uniquely identified in the presence of MDM2. Selections were performed side-by-side with 12ca5 as a negative control. Sequences bearing the FxxxWxx(L/V) motif characteristic of MDM2-binding are indicated in purple. Sequences bearing the FxxxW motif, but may be mis-sequenced due to incomplete backbone fragmentation during MS/MS, are indicated in blue. Hot spot residues are underlined for clarity.

Supplementary Table 11. Side-by-side selections against 14-3-3γ and 12ca5 identify 17 sequences pulled down uniquely in the presence of 14-3-3.

Construct	Sequence										ALC (%)
**14-3-3.1**	D-Leu	Nph	β-Ser	Nph	pSer	Nph	β-Ser	β-Ala	Nph	Lys	99
**14-3-3.2**	Fph	Nva	Nph	Fph	pSer	Cpa	D-Gln	Fph	Fph	Lys	99
**14-3-3.3**	Fph	Fph	Hyp	Fph	pSer	Thz	β-Ser	β-Ala	Nph	Lys	97
**14-3-3.4**	Cha	Cpa	β-Ser	Thz	pSer	Cha	β-Ser	Nph	β-Ala	Lys	91
**14-3-3.5**	Cha	Cha	β-Ser	Fph	pSer	Thz	β-Ser	β-Ala	Hyp	Lys	91
**14-3-3.6**	Cha	Cha	β-Ser	Orn	pSer	Nph	β-Ser	β-Ser	Nph	Lys	90
**14-3-3.7**	Cha	Nph	Hyp	Fph	pSer	Thz	β-Thr	β-Ala	Nph	Lys	85
**14-3-3.8**	Cha	Fph	β-Ser	Cpa	pSer	β-Thr	Nph	β-Ala	Nph	Lys	85
**14-3-3.9**	Thz	Thz	Hyp	Thz	pSer	Nph	β-Ser	β-Ser	Nph	Lys	85
**14-3-3.10**	Cha	β-Ala	Nph	Aph	pSer	Nva	Aad	D-Gln	Nph	Lys	85
**14-3-3.11**	D-Gln	Cpa	Nph	Cpa	pSer	Fph	β-Ser	β-Ala	Nph	Lys	84
**14-3-3.12**	D-Lys	Nva	Nph	Thz	pSer	Nph	β-Ser	β-Ala	Nph	Lys	84
**14-3-3.13**	Fph	β-Ser	Hyp	Thz	pSer	Nph	β-Ser	β-Ser	Nph	Lys	83
**14-3-3.14**	Cha	Cpa	β-Ser	Orn	pSer	Fph	Cpa	β-Ser	Nph	Lys	82
**14-3-3.15**	Cha	β-Ala	β-Ser	Cpa	pSer	Fph	β-Ala	β-Ala	Nph	Lys	81
**14-3-3.16**	Cpa	Fph	Cha	Thz	pSer	Thz	Hyp	Aph	Hyp	Lys	81
**14-3-3.17**	Cha	Fph	β-Ser	Cpa	pSer	Aph	β-Ser	Aad	Fph	Lys	81

List of all sequences identified in selections against 14-3-3γ that matched the library design with ALC ≥ 80. Residues held constant in the library are indicated in blue. Prominent amino acid motifs were identified, including a Cha-X-β-Ser motif at the N-term, and an a β-Ser-(β-Ala/β-Ser)-Nph motif at the C-term. Abbreviations: β-Ala = β-alanine; β-Ser = β-homoserine; β-Thr = β-homothreonine; Aad = aminoadipic acid; Aph = 4-aminophenylalanine; Cha = cyclohexylalanine; Cpa = cyclopropylalanine; Fph = 4-fluorophenylalanine; Hyp = hydroxyproline; Nph = 4-nitrophenylalanine; Orn = ornithine; pSer = phosphoserine; Thz = thiazolylalanine.

Supplementary Table 12. Data collection and refinement statistics for the 14-3-3σΔC/14-3-3.12 complex.

PDB Code	6TCH
**Data Collection**	
Wavelength	1.54
Resolution range	33.24 - 1.80 (1.87 - 1.80)
Space group	C 2 2 21
Unit cell *a*, *b*, *c* (Å)	82.4751<br>112.282<br>62.7582
Unit cell *α*, *β*, *γ* (°)	90<br>90<br>90
Total reflections	167691 (6320)
Unique reflections	27297 (2662)
Multiplicity	6.1 (4.8)
Completeness (%)	99.89 (99.22)
Mean I/sigma(I)	18.7 (5.5)
Wilson B-factor	11.26
R-merge	0.069 (0.258)
R-meas	0.075 (0.289)
R-pim	0.030 (0.129)
CC1/2	0.998 (0.952)
**Refinement**	
Reflections used in refinement	27296 (2662)
Reflections used for R-free	1377 (133)
R-work	0.1480 (0.1675)
R-free	0.1838 (0.2365)
Number of non-hydrogen atoms	2375
macromolecules	1930
ligands	94
solvent	351
Protein residues	236
RMS(bonds)	0.009
RMS(angles)	1.11
Ramachandran favored (%)	98.29
Ramachandran allowed (%)	1.71
Ramachandran outliers (%)	0.00
Rotamer outliers (%)	0.50
Clashscore	1.28
Average B-factor	15.84
macromolecules	13.00
ligands	33.40
solvent	26.79

Supplementary References:

1. Mijalis, A. J. *et al.* A fully automated flow-based approach for accelerated peptide synthesis. *Nature Chemical Biology* **13**, 464–466 (2017).
2. Simon, M. D. *et al.* Rapid Flow-Based Peptide Synthesis. *ChemBioChem* **15**, 713–720 (2014).
3. Vinogradov, A. A. *et al.* Library Design-Facilitated High-Throughput Sequencing of Synthetic Peptide Libraries. *ACS Comb. Sci.* **19**, 694–701 (2017).
4. Hartrampf, N. *et al.* Synthesis of proteins by automated flow chemistry. *Science* **368**, 980–987 (2020).
5. de Vink, P. J. *et al.* A Binary Bivalent Supramolecular Assembly Platform Based on Cucurbit[8]uril and Dimeric Adapter Protein 14-3-3. *Angew Chem Int Ed Engl* **56**, 8998–9002 (2017).
6. Moriarty, N. W., Grosse-Kunstleve, R. W. & Adams, P. D. electronic Ligand Builder and Optimization Workbench (eLBOW): a tool for ligand coordinate and restraint generation. *Acta Crystallogr. D Biol. Crystallogr.* **65**, 1074–1080 (2009).
7. Emsley, P. & Cowtan, K. Coot: model-building tools for molecular graphics. *Acta Crystallogr. D Biol. Crystallogr.* **60**, 2126–2132 (2004).
8. Adams, P. D. *et al.* PHENIX: a comprehensive Python-based system for macromolecular structure solution. *Acta Crystallogr. D Biol. Crystallogr.* **66**, 213–221 (2010).
9. Afonine, P. V. *et al.* Towards automated crystallographic structure refinement with phenix.refine. *Acta Crystallogr. D Biol. Crystallogr.* **68**, 352–367 (2012).

10. Gates, Z. P. *et al.* Xenoprotein engineering via synthetic libraries. *PNAS* 201722633 (2018) doi:10.1073/pnas.1722633115.
